# Supplementary material for: Structural characterization of a soil viral auxiliary metabolic gene product – a functional chitosanase
Source: Nat Commun. 2022 Sep 19;13:5485. doi: 10.1038/s41467-022-32993-8 (PMC9485262; doi:10.1038/s41467-022-32993-8)
Supplement: Supplementary file 1 — Supplementary Information [file 41467_2022_32993_MOESM1_ESM.pdf]

Structural characterization of a soil viral auxiliary metabolic gene product – a functional chitosanase

Ruonan Wu[1*], Clyde A. Smith[2*], Garry W. Buchko[1,3], Ian K. Blaby[4], David Paez-Espino[5], Nikos C. Kyrpides[4], Yasuo Yoshikuni[4], Jason E. McDermott[1,6], Kirsten S. Hofmockel[1], John R. Cort[1,7], Janet K. Jansson[1**]

[1]Earth and Biological Sciences Directorate, Pacific Northwest National Laboratory, Richland, WA 99354, USA; [2]Stanford Synchrotron Radiation Lightsource, Stanford University, Menlo Park, CA 94025, USA; [3]School of Molecular Biosciences, Washington State University, Pullman, WA 99164, USA; [4]US Department of Energy Joint Genome Institute, Lawrence Berkeley National Laboratory, Berkeley, CA 94720, USA; [5]Mammoth Biosciences, Brisbane, CA 94005, USA; [6]Department of Molecular Biology and Immunology, Oregon Health & Science University, Portland, OR, 97239, USA; [7]Institute of Biological Chemistry, Washington State University, Pullman, Washington, USA

*Co-first authors

**Senior, corresponding author, janet.jansson@pnnl.gov

a

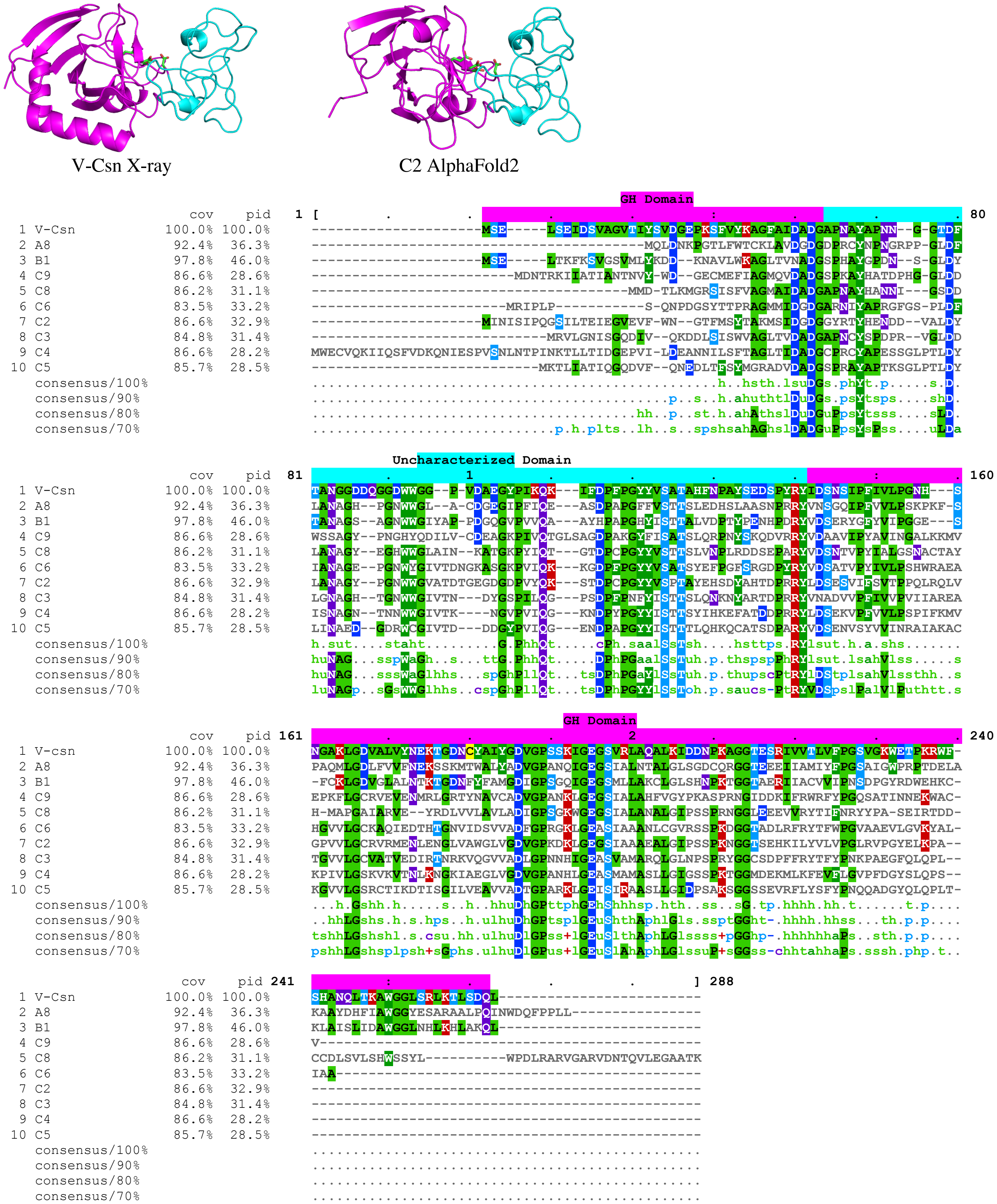

V-Csn X-ray
C2 AlphaFold2
GH Domain
Uncharacterized Domain
GH Domain
cov
pid
1 V-Csn 100.0% 100.0%
2 A8 92.4% 36.3%
3 B1 97.8% 46.0%
4 C9 86.6% 28.6%
5 C8 86.2% 31.1%
6 C6 83.5% 33.2%
7 C2 86.6% 32.9%
8 C3 84.8% 31.4%
9 C4 86.6% 28.2%
10 C5 85.7% 28.5%
consensus/100%
consensus/90%
consensus/80%
consensus/70%
1
80
81
160
161
240
241
288

b

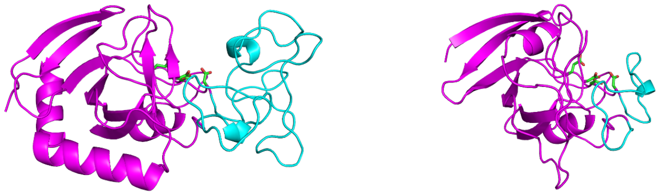


```
                                              GH Domain                  Uncharacterized Domain
                    cov     pid   1 [         .         .         .         :         .         .         . 80
1 V-Csn          100.0% 100.0%    ---------------MSELSEIDSVAGVTIYSVDGEPKSFVYKAGFAIDADGAPNAYAPNN--GGTDFTANGGDDQGGDW
2 A8              92.4%  36.3%    -----------------------------MQLDNKPGTLFWTCKLAVDGDGDPRCYNPNGRPPGLDFLANAGH--PGNW
3 B1              97.8%  46.0%    ---------------MSELTKFKSVGSVMLYKDD--KNAVLWKAGLTVNADGSPHAYGPDN--SGLDYTANAGS--AGNW
4 A2              64.7%  18.9%    -MKKLLTIYSK---GEHYSADLI------D--N-EDGS-GQFLADADIDIDGSPDWQRDPYGQADT-------------
5 A3              67.0%  15.6%    ----MATERKQQHIRAHNLEAILKIGGVEIYAD-PTGSYVCFVSDLDICNDGSGPAHGDPHHIKAT-------------
6 A4              68.3%  14.3%    MT--TRAKKKAQHIKKHQLSELITIGGVIIYAS-PKGDYVCFVSDLDVCTDGTGNHHGDNTPQDET-------------
7 A5              68.3%  15.5%    -MKLIYTHRSAENAQYVYYANIIRDDSRML--N-GKPC-YFWFGDADNDVDGAPYWNKDKYGQPGT-------------
8 A6              67.9%  11.7%    MTKRKITAKGQAHIKAHNLEVLLEIGGAIIYTA-PKGDYVCFVSDLDVCTDGSGPSHGDPSYQPQT-------------
  consensus/100%                  .....................................t.h.ahsths.s.DGss...ts.....th.............
  consensus/90%                   .....................................t.h.ahsths.s.DGss...ts.....th.............
  consensus/80%                   ....................t.h..........s..pss.hhahushslssDGss.h.tss...sth.............
  consensus/70%                   ...............hp.httlhp.suh.lhts.scss.hhahuchslssDGus.tassst..ssT.............

                                        Uncharacterized Domain               GH Domain
                    cov     pid  81         .         1         .         .         .         .         :         .
160
1 V-Csn          100.0% 100.0%    WGG--PVDAEGYPIKQKIFDPFPGYYVSATAHFNPAYSEDSPYRYIDSNSIPFIVLPGNH---SNGAKLGDVALVYNEKT
2 A8              92.4%  36.3%    WGL--ACDGEGIPFIQEASDPAPGFFVSTTSLEDHSLAASNPRRYVNSGQIPFVVLPSKP-KFSPAQMLGDLFVVFNEKS
3 B1              97.8%  46.0%    WGIYAPPDGQGVPVVQAAYHPAPGHYISTTALVDPTYPENHPDRYVDSERYGFYVIPGGE---S-FCKLGDVGLALNTKT
4 A2              64.7%  18.9%    -------------------------------------TLHHNGKPIDSESVPFFVLPPEVIKAFKGVILGCHGTIHYE--
5 A3              67.0%  15.6%    -------------------------------------AYKHNRKYLNADHDYYIVIPPQVRKLSRLKVMGCKSRLTNLTT
6 A4              68.3%  14.3%    -------------------------------------AYYNGGKFLNADVDKYIVIPPQIRSMVPPVVMGCQARLTQLDT
7 A5              68.3%  15.5%    -------------------------------------RWTYKGKPINGDIIPYIVVPPQMIKMTAEIVGGCVGTAEYE--
8 A6              67.9%  11.7%    -------------------------------------AYYNGGKFLNADVDKYIVIPPQVRSMVAPVVMGCQGKLTRLDS
  consensus/100%                  ............................................+.lsut...ahVlPst........hhGs..hh.....
  consensus/90%                   ............................................+.lsut...ahVlPst........hhGs..hh.....
  consensus/80%                   .....................................s..pst+.lsup...ahVlPsp....s..hhhGs.uhhh....
  consensus/70%                   .....................................shppst+alsu-...alVlPsph.phs..hhhGshuhlhp.po

                                                             GH Domain
                    cov     pid 161         .         .         .         2         .         .         .         .
240
1 V-Csn          100.0% 100.0%    GDNCYAIYGDVGPSSKIGEGSVRLAQALKIDDNP-KAGGTESRIVVTLVFPGSVGKWETPKRWF-SHANQLTKAWGGLSR
2 A8              92.4%  36.3%    SKMTWALYADVGPANQIGEGSIALNTALGLSGDC-QRGGTEEEIIAMIYFPGSAIGWPRPTDELAKAAYDHFIAWGGYES
3 B1              97.8%  46.0%    GDNFYFAMGDIGPSGQIGEGSMLLAKCLGLSHNP-KTGGTAERIIACVVIPNSDPGYRDWEHKC-KLAISLIDAWGGLNH
4 A2              64.7%  18.9%    GKSIDAVCADVGPHSKLGEISPEAARRLGINDDP-NIGGEDEQRVLYRFWPGKPAIVD--GIAY-----QLQPFTK----
5 A3              67.0%  15.6%    GGSHDAVCGEIGPPNKTGEASYCLARKVNAS--VTHNSGDKRRHYLYELWPGVPAAVN--GKHY-----NLV--------
6 A4              68.3%  14.3%    QQASPAVTGEIGPDDKTGEAAYCLCKLMNKA--LTANNGDTRRIYFYELWPGKAAVVD--GKKY-----KLEAAG-----
7 A5              68.3%  15.5%    DKIVGCVVGDSGPSSKIGELSPAALRELGLPAPENGNGGLDTQEILYRIWPGVAARIVRNGDVY-----QFELQHS----
8 A6              67.9%  11.7%    ALQHAAVVGEIGPKDKTGEAAICLAKIMNPK--VTPKAGDERLIYFYELWPGKPAVVD--GKRY-----KLEPA------
  consensus/100%                  t....hhhu-.GP.sphGEhu..h.p.ht........sG.tp..hhh.hhPs.s.hh...t..h.....ph.........
  consensus/90%                   t....hhhu-.GP.sphGEhu..h.p.ht........sG.tp..hhh.hhPs.s.hh...t..h.....ph.........
  consensus/80%                   st.h.slhu-lGPtsphGEhu.thsphhshs....ttuGppp phhhh.haPG.sshh...tchh.....ph.........
  consensus/70%                   upth.AlhG-lGPssKhGEuShtLs+tlshs..s.tpuGsccphhhh.laPGpsuhhs..scta.....pL..th.....

                    cov     pid 241         :       ] 257
1 V-Csn          100.0% 100.0%    LKTLSDQL---------
2 A8              92.4%  36.3%    ARAALPQINWDQFPPLL
3 B1              97.8%  46.0%    LKHLAKQL---------
4 A2              64.7%  18.9%    -----------------
5 A3              67.0%  15.6%    -----------------
6 A4              68.3%  14.3%    -----------------
7 A5              68.3%  15.5%    -----------------
8 A6              67.9%  11.7%    -----------------
  consensus/100%                  .................
  consensus/90%                   .................
  consensus/80%                   .................
  consensus/70%                   .................
```

Supplementary Figure 1 Viral chitosanase protein structure comparisons and partial sequence alignments. **a** Viral AMG chitosanase sequences predicted to have the uncharacterized domain (cyan) and GH domain (magenta) that are similar to those of the V-Csn X-Ray structure. Alignment made with MView 1.63. Conserved active site residues shown as sticks. **b** Viral AMG chitosanase sequences with and without the uncharacterized domain

structure seen in V-Csn (cyan). GH domain in magenta. Sequences lacking the novel domain may also lack the two prominent helices in the GH domain at the C-terminus of the sequence, or a shorter C-terminal helix may be poorly predicted in the structure shown. Conserved active site residues shown as sticks.

Supplementary Table 1 Metadata of viral contigs detected with Glycoside Hydrolase Family 75 chitosanase

The metadata includes 1) information of habitat (‘Ecosystem’ and ‘Habitat’) and IMG/VR (v3) dataset (‘Sample ID’ and ‘JGI Proposal Award DOI’ if any) where the viral contigs were detected; 2) quality of the viral contigs (‘Quality’ and ‘Completeness’); 3) bioinformatically predicted hosts (‘Host assignment’); 4) statistics of chitosanase annotations (‘E-value’ and ‘Bit score’); 5) gene arrangement of each viral contigs (‘Upstream’ and ‘Downstream’); 6) assigned AMG category (‘AMG category’) and 7) viral chitosanase IDs used in this study (‘Short name’).

Viral Contig	Length	Sample ID	UViG	Habitat	Ecosystem	Quality (checkV)	Completeness	Host assignment	Putative viral chitosanase ID	E-value	Bit score	Annotation	Upstream (H:hallmark, O:bact;X:NA; V:viral protein)	Downstream (H:hallmark, O:bact;X:NA; V:viral protein)	AMG category	Short Name	JGI Proposal Award DOI
3300000364_____INPhiseqgaiiFebDRAFT_101854920	22176	3300000364	IMGVR_UViG_3300000364_000020	Terrestrial	Grassland	Low-quality	13.69	Actinobacteria	3300000364_____INPhiseqgaiiFebDRAFT_101854920_13	2.39E-19	94.7	Chitosanase	VVXVVVXXVXVH	XVXXXXXXXXXXXXV	cat1	A1	
3300012349_____Ga0137387_10000020	56247	3300012349	IMGVR_UViG_3300012349_000002	Terrestrial	Unclassified	Medium-quality	62.57	Proteobacteria	3300012349_____Ga0137387_10000020_34	1.40E-07	25.1	Chitosanase	XXXXXXXXXXXXVXVXVVXVVVXXXXXXXXXXX	XOXXXVXOXVXHVVXXXXXXVVVVXVXVVXXXXXHVOXXXXX	cat1	A2	10.46936/10.25585/60000754
3300005937_____Ga0081455_10000703	43484	3300005937	IMGVR_UViG_3300005937_000048	Terrestrial	Rhizosphere	Medium-quality	62.52	Unclassified	3300005937_____Ga0081455_10000703_29	3.50E-07	23.8	Chitosanase	XXOXXXVXXXXVXXVXXXXXXXXXXXVXV	XXXXXXVXXXXXHXXVXOVXV	cat1	A3	
3300027874_____Ga0209465_10001056	12459	3300027874	IMGVR_UViG_3300027874_000074	Terrestrial	Forest	Low-quality	20.34	Proteobacteria	3300027874_____Ga0209465_10001056_2	2.40E-08	27.6	Chitosanase	H	XXXVVVOVHOXHXXXHX	cat0	A4	10.46936/10.25585/60000627
3300012971_____Ga0126369_10000332	30760	3300012971	IMGVR_UViG_3300012971_000062	Terrestrial	Forest	Low-quality	49.72	Proteobacteria	3300012971_____Ga0126369_10000332_32	2.00E-09	31.1	Chitosanase	XXXXXVXOXXXVXVXXXXXVXXXXXXXVXXXV	XXXXVVXXXXXXXXXHOVX	cat1	A6	10.46936/10.25585/60000627
3300005937_____Ga0081455_10000687	43973	3300005937	IMGVR_UViG_3300005937_000047	Terrestrial	Rhizosphere	Medium-quality	55.26	Proteobacteria	3300005937_____Ga0081455_10000687_48	5.00E-09	29.8	Chitosanase	XXOXXXXXXOXXXXVXXXXXXXOHXXXVOXXXXXXXVXXXXXXVXXX	XHXXXXXVVXXXXXXXXH	cat0	A7	
3300010043_____Ga0126380_10000006	46305	3300010043	IMGVR_UViG_3300010043_000005	Terrestrial	Forest	Low-quality	46.25	Unclassified	3300010043_____Ga0126380_10000006_37	2.97E-52	180	Chitosanase	VVXVVXVXXOVXVXXXVXVVXXVXXXXVVXXVXVXX	XXXVXXVVXVXXVXXOXXOXXX	cat2	A8	10.46936/10.25585/60000627
3300012582_____Ga0137358_10000037	40490	3300012582	IMGVR_UViG_3300012582_000003	Terrestrial	Unclassified	Medium-quality	55.32	Proteobacteria	3300012582_____Ga0137358_10000037_18	2.90E-07	24.1	Chitosanase	XOXXXXVXXXXXVXXHO	XXXXXVXXXXVXXXXXXVXXXXXXVXVVXVXXXXH	cat0	B1	10.46936/10.25585/60000754
3300012926_____Ga0137401_10000034	40321	3300012926	IMGVR_UViG_3300012926_000010	Terrestrial	Unclassified	Medium-quality	54.44	Proteobacteria	3300012926_____Ga0137401_10000034_25	2.90E-07	24.1	Chitosanase	VXVXXXXHOXXXXVXXXXXVXXHO	XXXXXVXXXXVXXXXXXVXXXXXXVXVV	cat1	B1	10.46936/10.25585/60000754
3300032893_____Ga0335069_10000845	49235	3300032893	IMGVR_UViG_3300032893_000075	Terrestrial	Wetland	Medium-quality	55.42	Proteobacteria	3300032893_____Ga0335069_10000845_57	6.20E-15	49	Chitosanase	XXVXXXVXVHXHVXXXVXXXXOXXXXVXVVXOOXXXXVXXXXXXXXXXXXVVXXXXXV	XVXXXOXXXXXXXXXXXVXXX	cat1	B2	
3300010043_____Ga0126380_10000125	19766	3300010043	IMGVR_UViG_3300010043_000020	Terrestrial	Forest	Low-quality	28.66	Proteobacteria	3300010043_____Ga0126380_10000125_30	1.30E-14	48	Chitosanase	VXXVXXXXXXXHXXXXXXVXXXXVXXXXXX	XHV	cat0	B3/V-Csn	10.46936/10.25585/60000627
2088090014_____GPIPI_17305223	19386	2088090014	IMGVR_UViG_2088090014_000012	Terrestrial	Grassland	Low-quality	21.56	Proteobacteria	2088090014_____GPIPI_17305223_22	2.17E-12	74.7	Chitosanase	XVXXXXXXXXXXXXXXXXXVXX	VXXXXXXXVXXXVH	cat1		
3300000439_____TBL_comb48_EPIDRAFT_1003500	10425	3300000439	IMGVR_UViG_3300000439_000610	Aquatic	Freshwater	Low-quality	21.53	Proteobacteria	3300000439_____TBL_comb48_EPIDRAFT_1003500_8	1.41E-19	95.1	Chitosanase	XVXHHXX	HXXVV	cat0		
3300000553_____TBL_comb47_HYPODRAFT_10001047	36469	3300000553	IMGVR_UViG_3300000553_000131	Aquatic	Freshwater	Low-quality	36.05	Actinobacteria	3300000553_____TBL_comb47_HYPODRAFT_10001047_26	9.70E-15	82	Chitosanase	XXXXXXXXOOXXVVXXXXXXXVXHH	XXXXXXXXXX	cat2		
3300000730_____fpDRAFT_1008667	22351	3300000730	IMGVR_UViG_3300000730_000091	Engineered	Wastewater	Low-quality	27.32	Proteobacteria	3300000730_____fpDRAFT_1008667_26	1.62E-12	77	Chitosanase	XXXHXXXXHXHXXVXXXXXXXXXXV	XXXXXXVXVX	cat1		
3300002408_____B570J29032_109959582	34894	3300002408	IMGVR_UViG_3300002408_000907	Aquatic	Freshwater	Low-quality	15.22	Unclassified	3300002408_____B570J29032_109959582_20	2.45E-07	65.9	Chitosanase	XXXXXXXVXVXOHVXVVXX	HVXVVVXHHHVXXV	cat0		
3300002835_____B570J40625_100000081	121143	3300002835	IMGVR_UViG_3300002835_000034	Aquatic	Freshwater	Medium-quality	52.44	Firmicutes	3300002835_____B570J40625_100000081_20	2.45E-07	65.9	Chitosanase	XXXXXXXVXVXOHVXVVXX	[illegible]	cat0		10.46936/10.25585/60000533
3300003813_____Ga0007879_1000018	69197	3300003813	IMGVR_UViG_3300003813_000008	Aquatic	Freshwater	Complete	100	Proteobacteria	3300003813_____Ga0007879_1000018_47	1.67E-28	118	Chitosanase	XVXXHXXOXVXXXXXXXXVXXXXXVVXXVVVVVHXXVOXXXXXVVX	XXXXVXVVXXXXXVXXHXXX	cat0		10.46936/10.25585/60000543
3300003814_____Ga0007877_1000002	57807	3300003814	IMGVR_UViG_3300003814_000001	Aquatic	Freshwater	Medium-quality	77.19	Proteobacteria	3300003814_____Ga0007877_1000002_55	1.67E-28	118	Chitosanase	VXVVXXXXXVXXHXXOXVXXXXXXXXXVXXXXXVVXXVVVVVHXXVOXXXXXVVX	XXXXX	cat2		10.46936/10.25585/60000543
3300003814_____Ga0007877_1000132	14284	3300003814	IMGVR_UViG_3300003814_000054	Aquatic	Freshwater	Low-quality	12.43	Proteobacteria	3300003814_____Ga0007877_1000132_8	6.70E-11	35.9	Chitosanase	XOXXHVV	XXXXXXXXVHVXVXVHXX	cat0		10.46936/10.25585/60000543
3300003911_____JGI25405J52794_10000041	13884	3300003911	IMGVR_UViG_3300003911_000009	Terrestrial	Rhizosphere	Low-quality	20.9	Proteobacteria	3300003911_____JGI25405J52794_10000041_18	1.00E-08	28.8	Chitosanase	XVXXXXXXXXVVVHVXX	VVXXXX	cat1		10.46936/10.25585/60007461
3300004633_____Ga0066395_10000709	12344	3300004633	IMGVR_UViG_3300004460_000380	Terrestrial	Forest	Low-quality	20.14	Proteobacteria	3300004633_____Ga0066395_10000709_14	2.40E-08	27.6	Chitosanase	XHXOHVOVVVXXX	HHX	cat0		10.46936/10.25585/60000627
3300004806_____Ga0007854_10000095	70136	3300004806	IMGVR_UViG_3300004461_000248	Aquatic	Freshwater	Low-quality	32.47	Firmicutes	3300004806_____Ga0007854_10000095_18	3.45E-41	152	Chitosanase	VXXXVOVXXVXOXVXVX	XXHVXXVXXVXXXXXXXXVVXXVVVVXVXVXXVVXVXXVOOVVVXOVXXXVXXXXVXVHHVHHVVXXV	cat1		10.46936/10.25585/60000543
3300005526_____Ga0073909_10000042	47262	3300005526	IMGVR_UViG_3300005526_000014	Terrestrial	Unclassified	Low-quality	44.58	Proteobacteria	3300005526_____Ga0073909_10000042_8	2.30E-05	50	Chitosanase	XXXVVXX	XHXHXXVXXXXXXXXXXXXVXXXXXVXXXXVXVXXXXXXXHXXXXXXXXXXXVXXXXXVXXXXXX	cat1	C1	10.46936/10.25585/60007659
3300005526_____Ga0073909_10000096	32586	3300005526	IMGVR_UViG_3300005526_000019	Terrestrial	Unclassified	Low-quality	29.43	Acidobacteria	3300005526_____Ga0073909_10000096_22	5.14E-32	127	Chitosanase	VXVXXXXXXXXXXXVXXHHXX	VXXVXVXXXXXXX	cat1		10.46936/10.25585/60007659
3300005526_____Ga0073909_10000172	23796	3300005526	IMGVR_UViG_3300005526_000031	Terrestrial	Unclassified	Low-quality	22.09	Unclassified	3300005526_____Ga0073909_10000172_25	9.80E-08	25.6	Chitosanase	XXXXXXXXHXXXXXXXVVVVVVXX	VHXXVXV	cat0		10.46936/10.25585/60007659
3300005764_____Ga0066903_100000166	34364	3300005764	IMGVR_UViG_3300005764_000047	Terrestrial	Forest	Low-quality	48.36	Proteobacteria	3300005764_____Ga0066903_100000166_28	2.00E-09	31.1	Chitosanase	XVVXHHVVXXVVOHXXXXXXXXXVVXXX	VXXXVXXXXXXVXXXXXVXVXXX	cat1		10.46936/10.25585/60000627
3300005937_____Ga0081455_10000183	78949	3300005937	IMGVR_UViG_3300005937_000004	Terrestrial	Rhizosphere	Medium-quality	69.56	Proteobacteria	3300005937_____Ga0081455_10000183_26	1.00E-08	28.8	Chitosanase	XVXXXXXXVXXXXXXXXOXXXXXXV	[illegible]	cat1		
3300006101_____Ga0007810_1000012	57820	3300006101	IMGVR_UViG_3300006101_000003	Aquatic	Freshwater	Medium-quality	77.2	Proteobacteria	3300006101_____Ga0007810_1000012_55	1.67E-28	118	Chitosanase	VXVVXXXXXVXXHXXOXVXXXXXXXXXVXXXXXVVXXVVVVVHXXVOXXXXXVVX	XXXXV	cat1		10.46936/10.25585/60000543
3300006104_____Ga0007882_10000071	57826	3300006104	IMGVR_UViG_3300006104_000001	Aquatic	Freshwater	Medium-quality	77.21	Proteobacteria	3300006104_____Ga0007882_10000071_14	1.67E-28	118	Chitosanase	HXXVOXXXXXVVX	XXXXVXVVXXXXXVXXHXXOXVXXXXXXXXXVXXXXXVVXXVVVVVX	cat0		10.46936/10.25585/60000543
3300006104_____Ga0007882_10001047	16350	3300006104	IMGVR_UViG_3300006104_000135	Aquatic	Freshwater	Low-quality	12.92	Proteobacteria	3300006104_____Ga0007882_10001047_17	6.70E-11	35.9	Chitosanase	XXHVXVXVHVXXXXXX	V	cat1		10.46936/10.25585/60000543
3300006105_____Ga0007819_1000232	19003	3300006105	IMGVR_UViG_3300006105_000159	Aquatic	Freshwater	Low-quality	19.94	Proteobacteria	3300006105_____Ga0007819_1000232_18	6.70E-11	35.9	Chitosanase	XHVVOVHXVXVOXXHVV	XXXXXXXXVHVXVXVHXX	cat0		10.46936/10.25585/60000543
3300006109_____Ga0007870_1000022	70904	3300006109	IMGVR_UViG_3300006109_000008	Aquatic	Freshwater	Low-quality	32.82	Firmicutes	3300006109_____Ga0007870_1000022_19	3.45E-41	152	Chitosanase	OVXXXVOVXXVXOXVXVX	XXHVXXVXXVXXXXXXXXVVXXVVVVXVXVXXVVXVXXVOOVVVXOVXXXVXXXXVXVHHVHHVVXXV	cat1		10.46936/10.25585/60000543
3300006110_____Ga0007871_1000025	57806	3300006110	IMGVR_UViG_3300006110_000001	Aquatic	Freshwater	Medium-quality	77.19	Proteobacteria	3300006110_____Ga0007871_1000025_23	1.67E-28	118	Chitosanase	VXOXXHXXVXXXXXVVXVXXXX	XVVXXXXXOVXXHVVVVVXXVVXXXXXVXXXXXXXX	cat0		10.46936/10.25585/60000543
3300006111_____Ga0007848_1000027	70136	3300006111	IMGVR_UViG_3300006111_000006	Aquatic	Freshwater	Low-quality	32.47	Firmicutes	3300006111_____Ga0007848_1000027_18	3.45E-41	152	Chitosanase	VXXXVOVXXVXOXVXVX	XXHVXXVXXVXXXXXXXXVVXXVVVVXVXVXXVVXVXXVOOVVVXOVXXXVXXXXVXVHHVHHVVXXV	cat1		10.46936/10.25585/60000543
3300006117_____Ga0007818_1000028	57786	3300006117	IMGVR_UViG_3300006113_000052	Aquatic	Freshwater	Medium-quality	77.18	Proteobacteria	3300006117_____Ga0007818_1000028_21	1.67E-28	118	Chitosanase	OXXHXXVXXXXXVVXVXXXX	XVVXXXXXOVXXHVVVVVXXVVXXXXXVXXXXXXXXVXV	cat0		10.46936/10.25585/60000543
3300006120_____Ga0007867_1000028	73640	3300006120	IMGVR_UViG_3300006120_000018	Aquatic	Freshwater	Low-quality	34.28	Firmicutes	3300006120_____Ga0007867_1000028_67	3.45E-41	152	Chitosanase	[illegible]	XVXVXOXVXXVOVXXXVOOVV	cat1		10.46936/10.25585/60000543
3300006854_____Ga0075425_100000201	53778	3300006854	IMGVR_UViG_3300006854_000008	Terrestrial	Rhizosphere	Medium-quality	64.82	Proteobacteria	3300006854_____Ga0075425_100000201_58	7.68E-16	84.3	Chitosanase	XXOVOXXXOXXXXXVXXXXXOXVXXVXXVXXVXXXXXVXVOXVVXXVVXXXXXXXXVXX	XXXXXXXXXXV	cat2		10.46936/10.25585/60000910
3300007076_____Ga0075435_100000075	51297	3300007076	IMGVR_UViG_3300007076_000010	Terrestrial	Rhizosphere	Medium-quality	55.71	Proteobacteria	3300007076_____Ga0075435_100000075_12	7.68E-16	84.3	Chitosanase	VXXXXXXXXXX	XXVXXXXXXXVVXXVVXOVXVXXXXXVXXVXXVXXVXOXXXXXXVXXXXXOXXXOX	cat2		10.46936/10.25585/60000910
3300009154_____Ga0114963_10000394	29301	3300009154	IMGVR_UViG_3300009154_000202	Aquatic	Freshwater	Low-quality	27.06	Proteobacteria	3300009154_____Ga0114963_10000394_11	2.83E-15	82	Chitosanase	XXXXXVXXXX	VVXXXXXXXOVXXXXXXXXXVXOVXXVXXXXXXXXVXXX	cat2	C10	10.46936/10.25585/60000587
3300009158_____Ga0114977_10000687	22394	3300009158	IMGVR_UViG_3300009158_000221	Aquatic	Freshwater	Low-quality	31.76	Proteobacteria	3300009158_____Ga0114977_10000687_27	3.40E-09	30.4	Chitosanase	XHXHVHVXXXXXXXXVXXXXXVXXXX	XXXXV	cat1		10.46936/10.25585/60000587
3300009159_____Ga0114978_10000954	24227	3300009159	IMGVR_UViG_3300009159_000310	Aquatic	Freshwater	Low-quality	30.48	Unclassified	3300009159_____Ga0114978_10000954_18	2.70E-10	33.9	Chitosanase	VVVVXXXOVXXXXXXVX	XXVVOXXHVV	cat1		10.46936/10.25585/60000587
3300009164_____Ga0114975_10000755	23970	3300009164	IMGVR_UViG_3300009164_000356	Aquatic	Freshwater	Low-quality	37.28	Proteobacteria	3300009164_____Ga0114975_10000755_9	2.80E-09	30.6	Chitosanase	XXXVXXXX	XXXXXXXXXHXXXXXVXXOHOHXHX	cat1		10.46936/10.25585/60000587
3300009181_____Ga0114969_10000130	62046	3300009181	IMGVR_UViG_3300009181_000079	Aquatic	Freshwater	Medium-quality	70.56	Acidobacteria	3300009181_____Ga0114969_10000130_90	2.50E-07	24.3	Chitosanase	[illegible]	XXXXX	cat2	C6	10.46936/10.25585/60000587
3300009184_____Ga0114976_10000585	22844	3300009184	IMGVR_UViG_3300009184_000231	Aquatic	Freshwater	Low-quality	35.53	Proteobacteria	3300009184_____Ga0114976_10000585_26	2.80E-09	30.6	Chitosanase	XHXHOHOXXVXXXXXHXXXXXXXXX	XXXXV	cat1		10.46936/10.25585/60000587
3300009184_____Ga0114976_10000610	22394	3300009184	IMGVR_UViG_3300009184_000240	Aquatic	Freshwater	Low-quality	31.76	Proteobacteria	3300009184_____Ga0114976_10000610_27	3.40E-09	30.4	Chitosanase	XHXHVHVXXXXXXXXVXXXXXVXXXX	XXXXV	cat1		10.46936/10.25585/60000587
3300009185_____Ga0114971_10000002	202880	3300009185	IMGVR_UViG_3300027746_000013	Aquatic	Freshwater	Medium-quality	78.05	Firmicutes	3300009185_____Ga0114971_10000002_127	2.10E-07	24.5	Chitosanase	[illegible]	[illegible]	cat0	C12	10.46936/10.25585/60000587
3300009502_____Ga0114951_10000439	58118	3300009502	IMGVR_UViG_3300009502_000059	Aquatic	Freshwater	Medium-quality	65.81	Proteobacteria	3300009502_____Ga0114951_10000439_63	2.70E-10	34	Chitosanase	XXVVXXOXXXXXOOXXXXXXXXVXVXOVXVOXVXXXXXVVXXXXXXXXXXXXXXXXXXXXXVO	XXXXXXHV	cat1		10.46936/10.25585/60000876
3300009692_____Ga0116171_10006394	10189	3300009692	IMGVR_UViG_3300009692_000215	Engineered	Wastewater	Low-quality	11.37	Proteobacteria	3300009692_____Ga0116171_10006394_12	1.25E-38	145	Chitosanase	XOXVXXXVOXV	VXXVXVXVV	cat2	C8	10.46936/10.25585/60000776
3300010048_____Ga0126373_10000281	33938	3300010048	IMGVR_UViG_3300010048_000028	Terrestrial	Forest	Low-quality	34.63	Proteobacteria	3300010048_____Ga0126373_10000281_42	7.90E-10	32.4	Chitosanase	XXXXVXXVVXVXXXXXXXXXXVXXXXXXVVXVVXVVXVXVXX	OVXXXXXXV	cat2		10.46936/10.25585/60000627
3300010233_____Ga0136235_1000158	34005	3300010233	IMGVR_UViG_3300010233_000129	Aquatic	Freshwater	Low-quality	39.54	Unclassified	3300010233_____Ga0136235_1000158_39	2.10E-19	94.2	Chitosanase	XXXVVXVXXXXXVXXXOXVVXXXXVVXVXVXXXXXXXXXX	XXXXXVX	cat2		
3300010233_____Ga0136235_1000189	31809	3300010233	IMGVR_UViG_3300010233_000155	Aquatic	Freshwater	Medium-quality	51.89	Proteobacteria	3300010233_____Ga0136235_1000189_28	8.55E-16	82.4	Chitosanase	XOVVXXXXVVXXXXXXXXXXVVXXXXXX	XXXXXXXXVVVXXXXVXVVOXXVXXXX	cat2		
3300010233_____Ga0136235_1000369	24098	3300010233	IMGVR_UViG_3300010233_000271	Aquatic	Freshwater	Low-quality	12.09	Unclassified	3300010233_____Ga0136235_1000369_4	2.71E-13	75.5	Chitosanase	XVV	XXXXXXXXVXXXXXXXXXVHXXVXXX	cat1		
3300010361_____Ga0126378_10000419	31668	3300010361	IMGVR_UViG_3300010361_000026	Terrestrial	Forest	Low-quality	42.46	Proteobacteria	3300010361_____Ga0126378_10000419_32	1.60E-08	28.2	Chitosanase	XXVXHHVVXXXXOHXXXXXXXXXXVXXVXXX	XXXXXXXXXXXVV	cat1		10.46936/10.25585/60000627
3300010362_____Ga0126377_10000082	45317	3300010362	IMGVR_UViG_3300010362_000034	Terrestrial	Forest	Medium-quality	57.44	Proteobacteria	3300010362_____Ga0126377_10000082_45	1.49E-06	57.4	Chitosanase	XXXXOXHXVVXXXXXXXVXXXXXHXXXVVXXXXXXXXXXXXXVOX	XXXXXXXXXXHXXXXXOXXXXXX	cat0		10.46936/10.25585/60000627
3300010362_____Ga0126377_10000413	25645	3300010362	IMGVR_UViG_3300010362_000097	Terrestrial	Forest	Low-quality	25.12	Verrucomicrobia	3300010362_____Ga0126377_10000413_12	2.00E-07	24.6	Chitosanase	XXXXXVXXHVV	VVHXVXXVXXXXXXXXXXXXXVXXVXXXXXOX	cat0		10.46936/10.25585/60000627
3300010366_____Ga0126379_10000347	25214	3300010366	IMGVR_UViG_3300010366_000063	Terrestrial	Forest	Low-quality	41.26	Proteobacteria	3300010366_____Ga0126379_10000347_19	2.90E-11	37.1	Chitosanase	XXHXXXHHXXHOHVHXXV	HHVOVXXX	cat0		10.46936/10.25585/60000627
3300010366_____Ga0126379_10000390	23968	3300010366	IMGVR_UViG_3300010366_000067	Terrestrial	Forest	Low-quality	30.74	Unclassified	3300010366_____Ga0126379_10000390_18	2.90E-08	27.3	Chitosanase	XXVXXXXXXXOOOVOXV	VVXVHXVXXXHVVVX	cat1		10.46936/10.25585/60000627
3300010376_____Ga0126381_100000274	57131	3300010376	IMGVR_UViG_3300010376_000011	Terrestrial	Forest	Complete	100	Proteobacteria	3300010376_____Ga0126381_100000274_36	1.22E-05	54.7	Chitosanase	XXXXXXXVXXXHHXHVXHXHXXXHHXXHOVVHXXV	HHXVXVVOOOXXXXXVXXXVXVXXVXVXXVXXXXXXXVXXXXV	cat0		10.46936/10.25585/60000627
3300010376_____Ga0126381_100000306	54539	3300010376	IMGVR_UViG_3300010376_000041	Terrestrial	Forest	Low-quality	45.3	Proteobacteria	3300010376_____Ga0126381_100000306_58	1.10E-07	25.5	Chitosanase	HXXXXVVOXVXXXXXXXXXXXXXXXXXXXXXXXXXXOXXXXXXXXXVXXXVXXXXXVXXXX	XXXVHXXVXXXXXXXVVXXXXVVVXXV	cat0		10.46936/10.25585/60000627
3300010376_____Ga0126381_100000470	44585	3300010376	IMGVR_UViG_3300010376_000059	Terrestrial	Forest	Low-quality	44.93	Proteobacteria	3300010376_____Ga0126381_100000470_39	1.80E-08	28	Chitosanase	XVXXXVXVXXXXXXXXXXXOXXXVXXXXXXXXXXXVXVXV	XVXXXXOXXXXXXXXXXHXVXOOXXXHXX	cat1		10.46936/10.25585/60000627
3300012971_____Ga0126369_10000399	28364	3300012971	IMGVR_UViG_3300012971_000071	Terrestrial	Forest	Low-quality	46.89	Proteobacteria	3300012971_____Ga0126369_10000399_5	3.90E-09	30.2	Chitosanase	XXHH	XXXVVVVVOXHXXXHHXXXHXHXVHXXHX	cat0		10.46936/10.25585/60000627
3300012971_____Ga0126369_10004632	10108	3300012971	IMGVR_UViG_3300012971_000182	Terrestrial	Forest	Low-quality	16.21	Proteobacteria	3300012971_____Ga0126369_10004632_5	5.60E-09	29.7	Chitosanase	XXHH	XXXXVVHVVV	cat0		10.46936/10.25585/60000627
3300019888_____Ga0193751_1000386	35792	3300019888	IMGVR_UViG_3300019888_000013	Terrestrial	Freshwater	Medium-quality	55.5	Proteobacteria	3300019888_____Ga0193751_1000386_16	6.75E-23	104	Chitosanase	XXHXXXXVVOXXXXX	XXXXXXXXVXVXXXXXVXXXOOVXHXOVVVVXXXHH	cat0		10.46936/10.25585/60000753
3300020062_____Ga0193724_1000025	32263	3300020062	IMGVR_UViG_3300020062_000007	Terrestrial	Freshwater	Low-quality	35.96	Proteobacteria	3300020062_____Ga0193724_1000025_14	3.20E-13	43.5	Chitosanase	XVXVVVVXXOXOX	XXVVXXXXXVXXXVXXXXXXXXXXXXVXVXXX	cat2		10.46936/10.25585/60000753
3300020550_____Ga0208600_1000021	20486	3300020550	IMGVR_UViG_3300020550_000017	Aquatic	Freshwater	Low-quality	8.92	Firmicutes	3300020550_____Ga0208600_1000021_2	2.45E-07	65.9	Chitosanase	H	XXVVXVHOXVXVXXXXXXV	cat0		10.46936/10.25585/60000533
3300020582_____Ga0210395_10003499	12029	3300020582	IMGVR_UViG_3300020582_000050	Terrestrial	Forest	Low-quality	12.77	Proteobacteria	3300020582_____Ga0210395_10003499_9	1.59E-45	163	Chitosanase	XXVXVVXV	VVVXV	cat2		10.46936/fics.proj.2016.49483/60006003
3300020699_____Ga0214221_1000072	43979	3300020699	IMGVR_UViG_3300020699_000007	Aquatic	Freshwater	Medium-quality	50.91	Verrucomicrobia	3300020699_____Ga0214221_1000072_41	9.33E-15	82	Chitosanase	XXXXXXXVVXOXXOXXXXXXXOVXXXVXXXXXXXXXXXXX	HHXXVX	cat1		10.46936/10.25585/60000533
3300020700_____Ga0214225_1000035	43969	3300020700	IMGVR_UViG_3300020700_000001	Aquatic	Freshwater	Medium-quality	54.11	Verrucomicrobia	3300020700_____Ga0214225_1000035_38	9.33E-15	82	Chitosanase	XXXXXVXXXVOXXXXXXXOXXOXVVXXXXXXXXVXXHH	XXXXXXXXX	cat2		10.46936/10.25585/60000533
3300020703_____Ga0214227_1000027	43858	3300020703	IMGVR_UViG_3300020703_000002	Aquatic	Freshwater	Medium-quality	50.77	Actinobacteria	3300020703_____Ga0214227_1000027_17	9.33E-15	82	Chitosanase	OXVVXXXXXXXXVXXHH	XXXXXXXXXXXXXVXXXVOXXXXXXXOXXX	cat1		10.46936/10.25585/60000533
3300020706_____Ga0214217_1000118	34792	3300020706	IMGVR_UViG_3300020706_000009	Aquatic	Freshwater	Low-quality	44.55	Actinobacteria	3300020706_____Ga0214217_1000118_28	9.33E-15	82	Chitosanase	XXXXXXXXOVXXXVXXXXXXXXXXXXX	HHXXVXXXXXXX	cat1		10.46936/10.25585/60000533
3300021124_____Ga0214199_1000026	35694	3300021124	IMGVR_UViG_3300021124_000015	Aquatic	Freshwater	Medium-quality	59.81	Unclassified	3300021124_____Ga0214199_1000026_7	1.62E-18	92.4	Chitosanase	VXXXHX	XXXXXXXVXXXXXXXXXXXVHOVXVXXOOXXOXXXXXXXXXXX	cat0		10.46936/10.25585/60000533
3300021139_____Ga0214166_1000301	26064	3300021139	IMGVR_UViG_3300021139_000125	Aquatic	Freshwater	Low-quality	47.43	Unclassified	3300021139_____Ga0214166_1000301_5	1.62E-18	92.4	Chitosanase	XXHX	XXXXXXXVXXXXXXVXXXVHOVXVXXX	cat0		10.46936/10.25585/60000533
3300021142_____Ga0214192_1000169	40324	3300021142	IMGVR_UViG_3300021142_000076	Aquatic	Freshwater	Medium-quality	83.86	Acidobacteria	3300021142_____Ga0214192_1000169_29	1.41E-19	95.1	Chitosanase	XXXXXXXXHXXXXXVHXXXXVVXVVXXH	XXHHXVXVXVXVVVO	cat0		10.46936/10.25585/60000533
3300021403_____Ga0210397_10000139	54296	3300021403	IMGVR_UViG_3300021403_000012	Terrestrial	Forest	Medium-quality	57.84	Proteobacteria	3300021403_____Ga0210397_10000139_99	1.42E-45	163	Chitosanase	[illegible]	V	cat1		10.46936/fics.proj.2016.49483/60006003
3300021404_____Ga0210389_10001107	22360	3300021404	IMGVR_UViG_3300021404_000041	Terrestrial	Forest	Not-determined	NA	Chloroflexi	3300021404_____Ga0210389_10001107_14	9.24E-47	166	Chitosanase	XXXVOOXXXXXVVX	XVVX	cat2		10.46936/fics.proj.2016.49483/60006003
3300021405_____Ga0210387_10000062	77711	3300021405	IMGVR_UViG_3300021405_000010	Terrestrial	Forest	Medium-quality	89.74	Chloroflexi	3300021405_____Ga0210387_10000062_108	4.92E-46	164	Chitosanase	[illegible]	V	cat1		10.46936/fics.proj.2016.49483/60006003
3300021405_____Ga0210387_10000245	39317	3300021405	IMGVR_UViG_3300021405_000031	Terrestrial	Forest	Medium-quality	61.82	Proteobacteria	3300021405_____Ga0210387_10000245_25	1.69E-45	163	Chitosanase	VXXXXXXVXXXXVOOXXVXVVXXX	VVVXVXXVVXXXX	cat2		10.46936/fics.proj.2016.49483/60006003
3300021478_____Ga0210402_10000026	135000	3300021478	IMGVR_UViG_3300021478_000017	Terrestrial	Forest	Medium-quality	60.74	Armatimonadetes	3300021478_____Ga0210402_10000026_183	1.20E-10	35.1	Chitosanase	[illegible]	XXXXXXH	cat0		10.46936/fics.proj.2016.49483/60006003
3300021478_____Ga0210402_10000601	39831	3300021478	IMGVR_UViG_3300021478_000071	Terrestrial	Forest	Medium-quality	66.95	Proteobacteria	3300021478_____Ga0210402_10000601_21	5.10E-10	33	Chitosanase	XXXXVXXVXXXXVXXXHXXX	XXXOOVXVXXVVXXXXXXHXXXXVVVXXVXXO	cat0		10.46936/fics.proj.2016.49483/60006003
3300021479_____Ga0210410_10001803	19682	3300021479	IMGVR_UViG_3300021479_000067	Terrestrial	Forest	Low-quality	24.78	Proteobacteria	3300021479_____Ga0210410_10001803_30	1.42E-45	163	Chitosanase	VXXXXXXXXXXXXXXVXXXXXVVXXVXVVV	VX	cat2		10.46936/fics.proj.2016.49483/60006003
3300021560_____Ga0126371_10000592	30037	3300021560	IMGVR_UViG_3300021560_000057	Terrestrial	Forest	Low-quality	21.32	Proteobacteria	3300021560_____Ga0126371_10000592_23	1.30E-54	187	Chitosanase	XXXXXVVXXOVXVXXXXXVXXXX	VVVVVXXVV	cat2		10.46936/10.25585/60000627
3300021560_____Ga0126371_10004477	12321	3300021560	IMGVR_UViG_3300021560_000128	Terrestrial	Forest	Low-quality	13.31	Unclassified	3300021560_____Ga0126371_10004477_11	2.69E-54	186	Chitosanase	XVVVXVXXXX	XXVXVVXXVH	cat1		10.46936/10.25585/60000627
3300022534_____Ga0224452_1000001	66947	3300022534	IMGVR_UViG_3300022534_000001	Aquatic	Freshwater	Medium-quality	80.85	Unclassified	3300022534_____Ga0224452_1000001_36	1.20E-18	92	Chitosanase	OXXXVXVXXHXXXXXXOXXXXXXXXXXXXXXXXXX	VXXVXXXXXVVVVOOVXVXXVOVXXXXXXXXXVXVXXXXXO	cat1		10.46936/10.25585/60000753
3300022555_____Ga0212088_10005809	22443	3300022555	IMGVR_UViG_3300022555_000266	Aquatic	Freshwater	Low-quality	31.08	Unclassified	3300022555_____Ga0212088_10005809_10	1.10E-08	28.8	Chitosanase	XXOXVXXXX	XXXXVXXXXXXXXXXXXOXVVXOHXHX	cat1		10.46936/10.25585/60000876
3300022602_____Ga0248169_101248	36528	3300022602	IMGVR_UViG_3300022602_000243	Aquatic	Freshwater	Medium-quality	61.07	Unclassified	3300022602_____Ga0248169_101248_4	2.40E-09	30.9	Chitosanase	XVV	XXXXXVXXVVXXXXVVVVVVHXXHXHVVXVXHXVXVVXXXXXVX	cat1	C3	
3300022694_____Ga0222623_10000004	67114	3300022694	IMGVR_UViG_3300022694_000001	Aquatic	Freshwater	Medium-quality	81.05	Firmicutes	3300022694_____Ga0222623_10000004_27	1.20E-18	91.8	Chitosanase	HXXXXXXOXXXXXXXXXXXXXXXXX	VXXVXXXXXVVVVOOVXVXXVOVXXXXXXXXXVXVXXXXXVXXXXVXVXXX	cat1		10.46936/10.25585/60000753
3300022745_____Ga0228698_1000621	23565	3300022745	IMGVR_UViG_3300022745_000143	Aquatic	Freshwater	Low-quality	15	Acidobacteria	3300022745_____Ga0228698_1000621_24	4.64E-21	100	Chitosanase	VXVOVVXXXXVXXXXXVXXXXOXX	VVXXOVXX	cat2		10.46936/10.25585/60000733
3300025162_____Ga0209083_1001017	34747	3300025162	IMGVR_UViG_3300025162_000112	Aquatic	Freshwater	Low-quality	42.34	Unclassified	3300025162_____Ga0209083_1001017_31	1.10E-08	28.8	Chitosanase	XVXOOVXXXXXVXXXXXXXXXXXOXVXXXX	XXXXVXXXXXXXXXXXXOXVVXOHXHX	cat1		10.46936/10.25585/60000876
3300025316_____Ga0209697_10002550	26047	3300025316	IMGVR_UViG_3300025316_000058	Aquatic	Freshwater	Low-quality	22.6	Unclassified	3300025316_____Ga0209697_10002550_20	2.25E-14	81.3	Chitosanase	XXXVOOVXOXXOOXXVXVX	XXXXXXXXVXXXXXXVXOX	cat2		10.46936/10.25585/60000876
3300025381_____Ga0208871_1000019	57914	3300025381	IMGVR_UViG_3300025381_000014	Aquatic	Freshwater	Medium-quality	77.28	Proteobacteria	3300025381_____Ga0208871_1000019_6	1.67E-28	118	Chitosanase	VXXXX	XVVXXXXXOVXXHVVVVVXXVVXXXXXVXXXXXXXXXVXOXXHXXVXXXXXVVXV	cat1		10.46936/10.25585/60000543
3300025390_____Ga0208743_1000010	128251	3300025390	IMGVR_UViG_3300025390_000003	Aquatic	Freshwater	Medium-quality	57.98	Firmicutes	3300025390_____Ga0208743_1000010_121	3.45E-41	152	Chitosanase	[illegible]	XVXVXOXVXXVOVXXXVV	cat1		10.46936/10.25585/60000543
3300025391_____Ga0208740_1000034	57831	3300025391	IMGVR_UViG_3300025391_000001	Aquatic	Freshwater	Medium-quality	77.21	Proteobacteria	3300025391_____Ga0208740_1000034_55	1.67E-28	118	Chitosanase	VXVVXXXXXVXXHXXOXVXXXXXXXXXVXXXXXVVXXVVVVVHXXVOXXXXXVVX	XXXXV	cat1		10.46936/10.25585/60000543
3300025395_____Ga0208109_1000076	56922	3300025395	IMGVR_UViG_3300025395_000010	Aquatic	Freshwater	Low-quality	27.14	Firmicutes	3300025395_____Ga0208109_1000076_60	3.45E-41	152	Chitosanase	XVXXXXVXXXVOXVVVOOVXXVXVVXXVXVXVVVVXXXVVXXXXXXXXXXVXXXVXXVHXX	XVXVXOXVXXVOVXXXVV	cat1		10.46936/10.25585/60000543
3300025400_____Ga0208387_1000027	57884	3300025400	IMGVR_UViG_3300025400_000021	Aquatic	Freshwater	Medium-quality	77.25	Proteobacteria	3300025400_____Ga0208387_1000027_6	1.67E-28	118	Chitosanase	VXXXX	XVVXXXXXOVXXHVVVVVXXVVXXXXXVXXXXXXXXXVXOXXHXXVXXXXXVVXV	cat1		10.46936/10.25585/60000543
3300025403_____Ga0208745_1000041	57849	3300025403	IMGVR_UViG_3300025403_000009	Aquatic	Freshwater	Medium-quality	77.23	Proteobacteria	3300025403_____Ga0208745_1000041_13	1.67E-28	118	Chitosanase	XXXXVVXVXXXX	XVVXXXXXOVXXHVVVVVXXVVXXXXXVXXXXXXXXXVXOXXHXXVX	cat1		10.46936/10.25585/60000543
3300025410_____Ga0208875_1000369	15454	3300025410	IMGVR_UViG_3300025410_000137	Aquatic	Freshwater	Low-quality	6.34	Unclassified	3300025410_____Ga0208875_1000369_6	3.45E-41	152	Chitosanase	XVXVX	XXHVXXVXXXVXXXXXXXXXXVVXXX	cat1		10.46936/10.25585/60000543
3300025416_____Ga0208877_1000289	16175	3300025416	IMGVR_UViG_3300025416_000080	Aquatic	Freshwater	Low-quality	18.58	Proteobacteria	3300025416_____Ga0208877_1000289_14	1.67E-28	118	Chitosanase	XXXXXVVXVXXXX	XVVXXXXXOVXX	cat2		10.46936/10.25585/60000543
3300025423_____Ga0208746_1000050	57920	3300025423	IMGVR_UViG_3300025423_000022	Aquatic	Freshwater	Medium-quality	77.28	Proteobacteria	3300025423_____Ga0208746_1000050_6	1.67E-28	118	Chitosanase	VXXXX	XVVXXXXXOVXXHVVVVVXXVVXXXXXVXXXXXXXXXVXOXXHXXVXXXXXVVXV	cat1		10.46936/10.25585/60000543
3300025430_____Ga0208622_1000032	57833	3300025430	IMGVR_UViG_3300025430_000002	Aquatic	Freshwater	Medium-quality	77.21	Proteobacteria	3300025430_____Ga0208622_1000032_55	1.67E-28	118	Chitosanase	VXVVXXXXXVXXHXXOXVXXXXXXXXXVXXXXXVVXXVVVVVHXXVOXXXXXVVX	XXXXV	cat1		10.46936/10.25585/60000543
3300025430_____Ga0208622_1000101	39000	3300025430	IMGVR_UViG_3300025430_000049	Aquatic	Freshwater	Medium-quality	64.63	Proteobacteria	3300025430_____Ga0208622_1000101_25	6.70E-11	35.9	Chitosanase	XVVVVVXXXXHVXVXVHVXXXXXX	VVHXXOVXVXHVOVVHXHXXHX	cat0		10.46936/10.25585/60000543
3300025648_____Ga0208507_1000129	57857	3300025648	IMGVR_UViG_3300025648_000029	Aquatic	Freshwater	Medium-quality	77.23	Proteobacteria	3300025648_____Ga0208507_1000129_46	1.67E-28	118	Chitosanase	VVVVVXXVVXXXXXVXXXXXXXXVXOXXHXXVXXXXXVVXVXXXX	XVVXXXXXOVXXH	cat0		10.46936/10.25585/60000543
3300025648_____Ga0208507_1000958	17019	3300025648	IMGVR_UViG_3300025648_000142	Aquatic	Freshwater	Low-quality	13.45	Proteobacteria	3300025648_____Ga0208507_1000958_17	6.70E-11	35.9	Chitosanase	XXHVXVXVHVXXXXXX	VX	cat1		10.46936/10.25585/60000543
3300025778_____Ga0208388_1000019	51482	3300025778	IMGVR_UViG_3300025778_000019	Aquatic	Freshwater	Medium-quality	57.29	Proteobacteria	3300025778_____Ga0208388_1000019_59	6.80E-08	26.1	Chitosanase	VXXXXXXXXXXHVXVXXXHVXXXXXXXXXXXXVXOXVVXHXVVXXXXXVXXXXXXXXXX	XXXVX	cat1		10.46936/10.25585/60000543
3300025783_____Ga0208258_1000037	57837	3300025783	IMGVR_UViG_3300025783_000009	Aquatic	Freshwater	Medium-quality	77.22	Proteobacteria	3300025783_____Ga0208258_1000037_53	1.67E-28	118	Chitosanase	VVXXXXXVXXHXXOXVXXXXXXXXXVXXXXXVVXXVVVVVHXXVOXXXXXVVX	XXXXVXX	cat1	C5	10.46936/10.25585/60000543
3300025789_____Ga0208499_1000259	21646	3300025789	IMGVR_UViG_3300025789_000177	Aquatic	Freshwater	Low-quality	27.63	Proteobacteria	3300025789_____Ga0208499_1000259_22	6.70E-11	35.9	Chitosanase	HXXHXHVVOVHXVXVOXXHVV	XXXXXXXXVHVXVXVHXX	cat0		10.46936/10.25585/60000543
3300025838_____Ga0208872_1000043	128554	3300025838	IMGVR_UViG_3300025838_000021	Aquatic	Freshwater	Medium-quality	58.11	Firmicutes	3300025838_____Ga0208872_1000043_121	3.45E-41	152	Chitosanase	[illegible]	XVXVXOXVXXVOVXXXVV	cat1	C4	10.46936/10.25585/60000543
3300026285_____Ga0209438_1000629	11005	3300026285	IMGVR_UViG_3300026285_000008	Terrestrial	Grassland	Low-quality	10.78	Proteobacteria	3300026285_____Ga0209438_1000629_3	7.40E-14	45.5	Chitosanase	XV	XVVXXXVXHXXXX	cat1		10.46936/10.25585/60000762
3300026285_____Ga0209438_1001046	8979	3300026285	IMGVR_UViG_3300026285_000009	Terrestrial	Grassland	Low-quality	13.57	Unclassified	3300026285_____Ga0209438_1001046_4	6.00E-13	42.6	Chitosanase	VVV	XVXXXXVXXVV	cat2		10.46936/10.25585/60000762
3300026304_____Ga0209240_1000230	22945	3300026304	IMGVR_UViG_3300026304_000007	Terrestrial	Grassland	Low-quality	17.79	Proteobacteria	3300026304_____Ga0209240_1000230_3	7.40E-14	45.5	Chitosanase	XV	XVVXXXVXHXXXXXXXXXXXXXXXHVV	cat1		10.46936/10.25585/60000762
3300026324_____Ga0209470_1001151	19943	3300026324	IMGVR_UViG_3300026324_000003	Terrestrial	Grassland	Low-quality	27.23	Proteobacteria	3300026324_____Ga0209470_1001151_22	1.62E-42	155	Chitosanase	XXVXXXVOXXXVXXVXXXXXX	XVHXV	cat1		10.46936/10.25585/60000762
3300026551_____Ga0209648_10005312	11014	3300026551	IMGVR_UViG_3300026551_000014	Terrestrial	Grassland	Low-quality	10.79	Proteobacteria	3300026551_____Ga0209648_10005312_14	7.40E-14	45.5	Chitosanase	XXXXHXVXXXXVVX	VX	cat1		10.46936/10.25585/60000762
3300027573_____Ga0208454_1000129	52778	3300027573	IMGVR_UViG_3300027573_000042	Aquatic	Freshwater	Medium-quality	66.46	Unclassified	3300027573_____Ga0208454_1000129_10	5.20E-08	26.5	Chitosanase	XXXXVXOXX	VXXXVXVVXXVVVVVXXXVXXXVVXOOXXVXXVVVXVXXV	cat2	C2	10.46936/10.25585/60000753
3300027655_____Ga0209388_1000050	19675	3300027655	IMGVR_UViG_3300027655_000004	Terrestrial	Rhizosphere	Low-quality	29.43	Proteobacteria	3300027655_____Ga0209388_1000050_4	4.70E-08	26.7	Chitosanase	XVX	XXXXHVXXXXOXXXVVXXXXVOXX	cat1		10.46936/10.25585/60000754
3300027712_____Ga0209499_1000398	32432	3300027712	IMGVR_UViG_3300027712_000088	Aquatic	Freshwater	Low-quality	48.14	Proteobacteria	3300027712_____Ga0209499_1000398_25	3.20E-09	30.4	Chitosanase	XHHVHOXXXXXXXXVXXXXXXXXX	XXXXVXXXXXXVVVXXXXVXXXXXXVO	cat1		10.46936/10.25585/60000587
3300027733_____Ga0209297_1000613	22211	3300027733	IMGVR_UViG_3300027733_000224	Aquatic	Freshwater	Low-quality	31.51	Proteobacteria	3300027733_____Ga0209297_1000613_6	3.40E-09	30.4	Chitosanase	VXXXX	XXXXVXXXXXVXXXXXXXXVHVHXHX	cat1		10.46936/10.25585/60000587
3300027734_____Ga0209087_1000582	22848	3300027734	IMGVR_UViG_3300027734_000331	Aquatic	Freshwater	Low-quality	35.54	Proteobacteria	3300027734_____Ga0209087_1000582_26	2.80E-09	30.6	Chitosanase	XHXHOHOXXVXXXXXHXXXXXXXXX	XXXXV	cat1		10.46936/10.25585/60000587
3300027734_____Ga0209087_1000604	22398	3300027734	IMGVR_UViG_3300027734_000334	Aquatic	Freshwater	Low-quality	31.77	Proteobacteria	3300027734_____Ga0209087_1000604_27	3.40E-09	30.4	Chitosanase	XHXHVHVXXXXXXXXVXXXXXVXXXX	XXXXV	cat1		10.46936/10.25585/60000587
3300027741_____Ga0209085_1000149	54249	3300027741	IMGVR_UViG_3300027741_000085	Aquatic	Freshwater	Medium-quality	69.47	Proteobacteria	3300027741_____Ga0209085_1000149_41	1.30E-08	28.4	Chitosanase	XXXXXVXXXXXXXXXVXXVXXXXVVVXXHVVXXVXVXXXX	XXXXXXXOXXXVXXXXXHXXXOXVX	cat0	C9	10.46936/10.25585/60000587
3300027746_____Ga0209597_1000003	202880	3300027746	IMGVR_UViG_3300027746_000013	Aquatic	Freshwater	Medium-quality	78.05	Firmicutes	3300027746_____Ga0209597_1000003_90	2.10E-07	24.5	Chitosanase	[illegible]	[illegible]	cat0		10.46936/10.25585/60000587
3300027754_____Ga0209596_1000127	66059	3300027754	IMGVR_UViG_3300027754_000083	Aquatic	Freshwater	Medium-quality	75.12	Acidobacteria	3300027754_____Ga0209596_1000127_18	2.50E-07	24.3	Chitosanase	XXXXVXXXXXXXXXXXX	XXXXXXXXXXXXXXHVXVXXXHXXXVXXXXXXXXXXVXXXXXXXXOXXXXVXXXXXXVXXXXVXXXXXXXXXXVXXXXXXXXOXVXXVXVXVX	cat1		10.46936/10.25585/60000587
3300027754_____Ga0209596_1000555	34546	3300027754	IMGVR_UViG_3300027754_000255	Aquatic	Freshwater	Low-quality	42.81	Unclassified	3300027754_____Ga0209596_1000555_48	1.20E-10	35.1	Chitosanase	VXXXXXXXXXXXXXXXHXVHXVXVXVXXVXXXXVXXVVXXOVXXXXX	XXXXX	cat2		10.46936/10.25585/60000587
3300027782_____Ga0209500_10002238	13950	3300027782	IMGVR_UViG_3300027782_000773	Aquatic	Freshwater	Low-quality	18.82	Unclassified	3300027782_____Ga0209500_10002238_10	2.70E-10	33.9	Chitosanase	VXXXXXVXX	XXXVVOXXHVXV	cat1		10.46936/10.25585/60000587
3300027821_____Ga0209811_10000270	24086	3300027821	IMGVR_UViG_3300027821_000031	Terrestrial	Unclassified	Low-quality	22.36	Unclassified	3300027821_____Ga0209811_10000270_8	9.80E-08	25.6	Chitosanase	VXVXXHV	XXVVVVVVXXXXXXXHXXXXXXXX	cat0		10.46936/10.25585/60007659
3300027911_____Ga0209698_10001340	28378	3300027911	IMGVR_UViG_3300027911_000054	Aquatic	Freshwater	Low-quality	38.61	Verrucomicrobia	3300027911_____Ga0209698_10001340_22	5.66E-63	206	Chitosanase	OOVXXVVXVXVXXXOOVXXHX	XXHOVVXXXXXXXXXXV	cat0	C11	10.46936/10.25585/60000806
3300027969_____Ga0209191_1000371	35468	3300027969	IMGVR_UViG_3300027969_000241	Aquatic	Freshwater	Low-quality	48	Proteobacteria	3300027969_____Ga0209191_1000371_28	2.80E-09	30.6	Chitosanase	XXOVXOVXVXXVXXXVVXXXXXVXXXX	XXXXXXXXXHXXXXXVXXOHOHXHX	cat1		10.46936/10.25585/60000587
3300028590_____Ga0247823_10000072	73933	3300028590	IMGVR_UViG_3300028590_000012	Terrestrial	Agricultural	Medium-quality	75.63	Proteobacteria	3300028590_____Ga0247823_10000072_83	8.10E-10	32.4	Chitosanase	[illegible]	XXXXXVXVXXVXXVVXXXXVVXXVXXXOVXXXVV	cat2		10.46936/10.25585/60000933
3300028590_____Ga0247823_10000159	56152	3300028590	IMGVR_UViG_3300028590_000019	Terrestrial	Agricultural	Medium-quality	59.93	Proteobacteria	3300028590_____Ga0247823_10000159_19	2.90E-08	27.3	Chitosanase	OXVVXXVVVVXXXXXXXX	HVXXVXXVXVXXVXXXXXXXXXXXXVXXXXVXXVXXXXXVXXXXXXVXXXXVXXXXXVXXXXXOXXOVXOVXXO	cat1		10.46936/10.25585/60000933
3300028590_____Ga0247823_10000343	40633	3300028590	IMGVR_UViG_3300028590_000046	Terrestrial	Agricultural	Medium-quality	60.9	Proteobacteria	3300028590_____Ga0247823_10000343_31	5.00E-10	33.1	Chitosanase	XHVVHXXOXXOXXXXXXXXXXXXXOXXXXX	VHXXXXXVXXVVVVXOXXXXVOXXVVXXVXX	cat0	C7	10.46936/10.25585/60000933
3300028592_____Ga0247822_10000132	33163	3300028592	IMGVR_UViG_3300028592_000054	Terrestrial	Agricultural	Medium-quality	51.07	Proteobacteria	3300028592_____Ga0247822_10000132_31	5.00E-10	33.1	Chitosanase	XHVVHXXOXXOXXXXXXXXXXXXXOXXXXX	VHXXXXXVXXVVVVXO	cat0		10.46936/10.25585/60000933
3300029798_____Ga0239581_1000219	52648	3300029798	IMGVR_UViG_3300029798_000007	Aquatic	Freshwater	Medium-quality	59.61	Proteobacteria	3300029798_____Ga0239581_1000219_64	2.70E-10	34	Chitosanase	XVXVVXXOXXXXXOOXXXXXXXXVXVXOVXVOXVXXXXXVVXXXXXXXXXXXXXXXXXXXXXVO	XXXXXXHX	cat1		
3300029922_____Ga0311363_10000523	78115	3300029922	IMGVR_UViG_3300029922_000048	Terrestrial	Permafrost	Low-quality	40.45	Actinobacteria	3300029922_____Ga0311363_10000523_93	7.80E-10	32.4	Chitosanase	[illegible]	XXXXXVXX	cat2		10.46936/fics.proj.2017.49950/60006215
3300029953_____Ga0311343_10000842	54266	3300029953	IMGVR_UViG_3300029953_000039	Terrestrial	Permafrost	Medium-quality	77.81	Proteobacteria	3300029953_____Ga0311343_10000842_75	2.22E-11	70.5	Chitosanase	OXXXXXXVVXXXXXXXOXVXXOXXXXOVXXVHXVXVXHXVXXXXXXXVXXVXXXXHXXXXOXXXVVXXXXXXXXXVXX	XXOXVXX	cat1		10.46936/fics.proj.2017.49950/60006215
3300031524_____Ga0302320_10005537	28283	3300031524	IMGVR_UViG_3300031524_000066	Terrestrial	Permafrost	Low-quality	21.63	Unclassified	3300031524_____Ga0302320_10005537_8	9.10E-11	35.5	Chitosanase	OXXXXXV	OVXXVXXXXXXXXXXVOXXVVXVOVXXXXVXXXXXXVXVX	cat2		10.46936/fics.proj.2017.49950/60006215
3300031788_____Ga0302319_10002010	44714	3300031788	IMGVR_UViG_3300031788_000036	Terrestrial	Permafrost	Low-quality	40.29	Firmicutes	3300031788_____Ga0302319_10002010_6	9.10E-11	35.5	Chitosanase	XXXXV	OVXXVXXXXXXXXVOXXVVXVXVXXXXXXXXVXVXVXVVVXXXXVXVVXXXXXV	cat2		10.46936/fics.proj.2017.49950/60006215
3300032605_____Ga0316232_1002407	19455	3300032605	IMGVR_UViG_3300032605_000189	Aquatic	Freshwater	Low-quality	20.15	Verrucomicrobia	3300032605_____Ga0316232_1002407_8	2.80E-07	24.1	Chitosanase	OVVVXXV	XXHXXXXXVXXXXXXOOXXXXXXXVXXXXH	cat1		10.46936/10.25585/60000907
3300032668_____Ga0316230_1001206	28878	3300032668	IMGVR_UViG_3300032668_000112	Aquatic	Freshwater	Low-quality	45.89	Verrucomicrobia	3300032668_____Ga0316230_1001206_42	2.80E-07	24.1	Chitosanase	VXXXXVHVVHXVHXXXXVXXXXXXOOXXXXXXVXXXXXHXX	VXXVVVO	cat1		10.46936/10.25585/60000907
3300032677_____Ga0316227_1000899	36737	3300032677	IMGVR_UViG_3300032677_000088	Aquatic	Freshwater	Low-quality	47.44	Proteobacteria	3300032677_____Ga0316227_1000899_11	2.40E-06	21.1	Chitosanase	XHVVXXXXXX	XXXXXXVXVXHVXHXHXXXVXXVXXVXXOXXXVXXX	cat0		10.46936/10.25585/60000907
3300032722_____Ga0316231_1001296	28329	3300032722	IMGVR_UViG_3300032722_000095	Aquatic	Freshwater	Low-quality	45.02	Verrucomicrobia	3300032722_____Ga0316231_1001296_42	2.80E-07	24.1	Chitosanase	VXXXXVHVVHXVHXXXXVXXXXXXOOXXXXXXVXXXXXHXX	VXXVVVO	cat1		10.46936/10.25585/60000907
3300032783_____Ga0335079_10001352	27822	3300032783	IMGVR_UViG_3300032783_000007	Terrestrial	Wetland	Low-quality	31.06	Actinobacteria	3300032783_____Ga0335079_10001352_24	3.02E-36	137	Chitosanase	XVXXXVXHXXXXXXXVHXXVXXX	XXXXOXXXXXXVXXXXXX	cat1		
3300032954_____Ga0335083_10000404	67143	3300032954	IMGVR_UViG_3300032954_000008	Terrestrial	Wetland	Low-quality	40.15	Proteobacteria	3300032954_____Ga0335083_10000404_39	2.88E-24	108	Chitosanase	XXOOOVVXVOVXXOXXXXVVVXXOXXXXXXXXXXXXX	XXXVXXOOXXXXXXXXXOXXXXXXXVXXVXXXVVXXVXXXXXXXX	cat2		
3300033158_____Ga0335077_10000474	44437	3300033158	IMGVR_UViG_3300033158_000003	Terrestrial	Wetland	Low-quality	46.38	Proteobacteria	3300033158_____Ga0335077_10000474_60	1.74E-48	170	Chitosanase	HVHXXXOXXXXXXXXXXXOXXXXOXXXVVXXXXXXXXHVXVXXXXVXXHXXXOXXXXXXXVX	XVXXXH	cat0		
