# Supplementary material for: Structural characterization of a soil viral auxiliary metabolic gene product – a functional chitosanase
Source: Nat Commun. 2022 Sep 19;13:5485. doi: 10.1038/s41467-022-32993-8 (PMC9485262; doi:10.1038/s41467-022-32993-8)
Supplement: Supplementary file 3 — Description of Additional Supplementary Files [file 41467_2022_32993_MOESM3_ESM.pdf]

File Name: Supplementary Data 1
Description: Multiple alignment of bacterial, fungal and viral chitosanase protein sequences. Sequences with headers containing ‘ref_bact’, ‘ref_fungi’ and ‘exp_virus’ are bacterial, fungal and viral chitosanase sequences, respectively. The chitosanase sequences were aligned with a phage lysozyme (YP_006987285, ‘OutGroup’) as an outgroup sequence for the phylogenetic tree construction. ‘-’ represents the gap within the alignments.
