# Supplementary material for: Structural characterization of a soil viral auxiliary metabolic gene product – a functional chitosanase
Source: Nat Commun. 2022 Sep 19;13:5485. doi: 10.1038/s41467-022-32993-8 (PMC9485262; doi:10.1038/s41467-022-32993-8)
Supplement: Supplementary file 4 — Supplementary Data 1 [file 41467_2022_32993_MOESM4_ESM.pdf]

Supplementary dataset

Structural characterization of a soil viral auxiliary metabolic gene product – a functional chitosanase

Ruonan Wu[1*], Clyde A. Smith[2*], Garry W. Buchko[1,3], Ian K. Blaby[4], David Paez-Espino[5], Nikos C. Kyrpides[4], Yasuo Yoshikuni[4], Jason E. McDermott[1,6], Kirsten S. Hofmockel[1], John R. Cort[1,7], Janet K. Jansson[1**]

[1]Earth and Biological Sciences Directorate, Pacific Northwest National Laboratory, Richland, WA 99354, USA; [2]Stanford Synchrotron Radiation Lightsource, Stanford University, Menlo Park, CA 94025, USA; [3]School of Molecular Biosciences, Washington State University, Pullman, WA 99164, USA; [4]US Department of Energy Joint Genome Institute, Lawrence Berkeley National Laboratory, Berkeley, CA 94720, USA; [5]Mammoth Biosciences, Brisbane, CA 94005, USA; [6]Department of Molecular Biology and Immunology, Oregon Health & Science University, Portland, OR, 97239, USA; [7]Institute of Biological Chemistry, Washington State University, Pullman, Washington, USA

*Co-first authors

**Senior, corresponding author, janet.jansson@pnnl.gov

>OutGroup_PhageLysozyme_YP_006987285.1/39-307
TSLVSQEAVVQQ----EPQ---ITIQKQPPVIIQIEPEEATVNRKQYKVLSG--DSLWTIANRI--KP--------------------HNVEVDEYVKVLKVV-------NSDVEMH-------------------PGTMF----EIPSADDLKEVTLPDVLVHFD-----------------
ITDETVINHIKK------AEGTSESQAVLKRRLLGGKVGPSFK----N-GKFYPYRDSTGHFTIGYGHYLGKSEKDAAKYRNGISKRQAHDLLLTDMQRTMNDFVLLLQRKRAVDLTVDQQRILYEMAFTLGVDKLSRFNKMWKSVENNNQHKFKTEIANSLWYKQMGNRAVMLV
>exp_virus_3300000364_____INPhiseqgaiiFebDRAFT_101854920_13/27-116
TGHVDADG-S--G---------------------------GN---------------------------------------PW-G--DPD--------------------FQPRTSLKR------PDG-----------------------------------NWLNANEERYIAVPPGVV--------S----------------------GV-------RPI-----------VLGCQAWVLYR-----
GHCV------------SA---------------------------VVADV------------------------------------GP-------------AG------------------KLGELSVACA
>exp_virus_3300012349_____Ga0137387_10000020_34/31-118
DADIDIDG-S--PDW----------QRDP-YGQADTTLH-H----------N-G--KP---I---DSESVPFFVLPPEVI--------K-----------------------AF-------KGV-----------ILGCHGTIHYE-----GKSI------
------DA----------------------------VCADV-----------------------------------GP--------------HS------------------KLGEISPEAA
>exp_virus_3300005937_____Ga0081455_10000703_29/40-129
DLDICNDG-S--GPA--------HGDP-HHIKATAYK-H------------N-R--KY---L---NADHDYYIVIPPQVR--------K--------------------LS-------RLK-----------VMGCKSRLTNLT--T-
GGSH------------DA---------------------------VCGEI----------------------------------GP--------------PN------------------KTGEASYCLA
>exp_virus_3300027874_____Ga0209465_10001056_2/42-131
DLDVCTDG-T--GNH----------HGDN-TPQDETAYY-N----------G-G--KF---L---NADVDKYIVIPPQIR--------S--------------------MV-------PPV-----------VMGCQARLTQLD--T-
QQAS------------PA---------------------------VTGEI----------------------------------GP--------------DD------------------KTGEAAYCLC
>exp_virus_3300012971_____Ga0126369_10000332_32/44-133
DLDVCTDG-S--GPS----------HGDP-SYQPQTAYY-N----------G-G--KF---L---NADVDKYIVIPPQVR--------S--------------------MV-------APV-----------VMGCQGKLTRLD--S-
ALQH------------AA---------------------------VVGEI----------------------------------GP--------------KD------------------KTGEAAICLA
>exp_virus_3300005937_____Ga0081455_10000687_48/36-125
DLDICNDG-S--GPS----------HGDP-SYQSQTAYY-N----------G-G--KY---L---NADKDKYIVIPPQVR--------K--------------------MV-------PPA-----------VMGCKARLTNLD--S-
KVTS------------AA---------------------------VTGEI----------------------------------GP--------------DD------------------KTGEAAYCLA
>exp_virus_3300010043_____Ga0126380_10000006_37/15-150
KLAVDGDG-D-------PR------CYNP------NGRP-----------PG----LDFLANA------------GH------------PGNWWGLAC--D-----------GE-G--IP---FIQEASD-----PAPGFFVSTTSLED------HSL-A-ASN------------PR-----------RYVNSGQIPFVVLPSKP---------K--------------------FS-------PAQ----
-------MLGDLFVVFNEK--S-SKMT------------WA------------------------LYADV-----------------------------------GP------------AN-----------------QIGEGSIALN
>exp_virus_3300012582_____Ga0137358_10000037_18/28-160
GLTVNADG-S-------PH------AYGP--------DN----------SG----LDYTANA------------G------------SAGNWWGIYAPPD----------GQ-G--VP---VVQAAYH-----PAPGHYISTTALVD------PTY-P-ENH------------PD-----------RYVDSERYGFYVIPG----------------------------------G-------ESFC--
--------KLGDVGLALNTK--T-GDNF-----------YF-------------------------AMGDI------------------------------------GP------------SG-----------------QIGEGSMLLA
>exp_virus_3300012926_____Ga0137401_10000034_25/28-160
GLTVNADG-S-------PH------AYGP--------DN----------SG----LDYTANA------------G------------SAGNWWGIYAPPD----------GQ-G--VP---VVQAAYH-----PAPGHYISTTALVD------PTY-P-ENH------------PD-----------RYVDSERYGFYVIPG----------------------------------G-------ESFC--
--------KLGDVGLALNTK--T-GDNF-----------YF-------------------------AMGDI------------------------------------GP------------SG-----------------QIGEGSMLLA
>exp_virus_B3_3300010043_____Ga0126380_10000125_30/30-163
GFAIDADG-A-------PN------AYAP--------NN----------GG----TDFTANG------------GD----------DQGGDWWGGPV--D----------AE-G--YP---IKQKIFD-----PFPGYYVSATAHFN------PAY-S-EDS------------PY-----------RYIDSNSIPFIVLPGNH-------------------------------S-------NGA----
-------KLGDVALVYNEK--T-GDNC-----------YA------------------------IYGDV------------------------------------GP------------SS-----------------KIGEGSVRLA
>ref_archaea_SNQ62943.1Ruonan267/77-180
SGAIDCDG--------QA-------------------------------------T-THC-----NS--N-T--DP------------------YYQGQTSFT------------QSD-------------------GR------P-LNAELLPWYVLPE------------------------TPNPIFDY-AT-------RNI----------YGGEAGVVLY----N-
DKME------------FG------------------------VFGDE----------------------------------RG------------RDGGNSAG----------LAIGEISYAMA
>ref_archaea_VVB54343.1Ruonan394/70-173
SGAIDCDG--------QA-------------------------------------T-IHC-----TG--T-T--DP------------------AYQGQTSFT------------QSN-------------------GE------P-LNAELLPWYVLPE------------------------TPNPIFDY-AT-------RDI----------HGGEAGVVLY----N-
GKME------------YG------------------------VFGDE----------------------------------RG------------RDGGNSAG----------LAIGEVSYAMA
>ref_fungi_RDW92923.1Ruonan825/97-184
DMDVDCDG-IDF--RC-----RG--N-S--D--------------------GLPETSW--GALAAYEVPFIVIPEQYQ-------EA-H------------------EA-------A-I----------PGNNIAAVIC----N-GKMF----
--------YG------------------------ILGDT--------------------------------NG-------------DSPE-------------VTGEASWLMA
>ref_fungi_PKY03197.1Ruonan386/99-186
DMDVDCDG-IDY--KC-----VG--N-P--D--------------------GQDQTNW--GALAAYEVPFIVIPQKFL-------SA-N------------------GN-------L-L----------SGNNIAAVIC----N-GKMY-
-----------YG------------------------ILGDS--------------------------------NG-------------DSPQ-------------VTGEASWLMA
>ref_fungi_BCS30771.1Ruonan303/96-183
DMDVDCDG-LDH--DC-----DG--N-P--D--------------------GQPQTNW--GSLAAYEVPFIVIPDEYL-------TQ-N------------------EE-------T-L----------SGNNIAAVIC----D-GKMF--
----------YG------------------------ILGDS--------------------------------NG-------------DEPQ-------------VTGEASWLMA
>ref_fungi_KAE8154786.1Ruonan292/97-184
DMDVDCDG-IND--KC-----DG--N-P--D--------------------GQPQTNW--GALSAYAVPYIVIPDRYL-------AA-N------------------KV-------L-L----------PGNNIAAVIC----N-
GQMY------------YG------------------------ILGDS--------------------------------NG-------------DSPQ-------------ITGEASWLMA
>ref_fungi_KAB8070890.1Ruonan288/99-186
DMDVDCDG-VNE--KC-----EG--N-Q--D--------------------GQPQTNW--GHLAAYAVPYIVIPDRFL-------TS-N------------------KE-------I-L----------PGNNVAAVIC----N-
KKMY------------YG------------------------ILGDS--------------------------------NG-------------DEPQ-------------ITGEASWLMA
>ref_fungi_KNG84729.1Ruonan1195/99-186
DMDVDCDG-VNH--KC-----EG--N-Q--D--------------------GQPQTNW--GALSAYAVPYIVIPDRFL-------AA-N------------------KD-------I-L----------PGNNIAAVIC----N-
GKMY------------YG------------------------ILGDS--------------------------------NG-------------DDPQ-------------ITGEASWLMA
>ref_fungi_GES60629.1Ruonan321/99-186
DMDVDCDG-INS--DC-----KG--S-T--T--------------------SQTQTNW--GALSAYEVPYIVTPDRFL-------SA-N------------------SE-------I-L----------PGNNVAAVIC----D-GKMF---
---------YG------------------------ILGDS--------------------------------NG-------------HDPQ-------------ITGEASWLMA
>ref_fungi_CEN60696.1Ruonan398/109-197
DMDVDCDG-IDY--KC-----DG--N-K--D--------------------GQALTDY--GALSAYAVPFIVIPQEYV-------SSGN------------------QD-------A-I----------PGNNVAAVIC----N-
GKMF------------YG------------------------ILGDT--------------------------------NG-------------NDPQ-------------VTGEASWLMA
>ref_fungi_BCS29405.1Ruonan291/92-179
DMDVDCDG-IDY--KC-----SG--N-A--D--------------------GQPVTNW--GALSATEVPYIVIPQAFL-------EA-N------------------PE-------A-I----------PGNNVAAVIC----N-NRMF---
---------YA------------------------ILGDT--------------------------------NG-------------NNPQ-------------VTGEASLLLA
>ref_fungi_RDW72465.1Ruonan338/87-174
DMDTDCDG-LDY--KC-----DG--N-A--D--------------------GQPLTNW--GALSAFEVPFIVIPQRFL-------EA-N------------------PK-------A-I----------PGNNVAAVIC----N-NKMF--
----------YG------------------------ILGDT--------------------------------NG-------------NDPQ-------------VTGEASWLLA
>ref_fungi_CEL02971.1Ruonan291/84-171
DMDIDCDG-INF--QC-----VG--N-L--D--------------------GQPLTNW--GALSAYEVPFIVIPNRFL-------EA-N------------------PT-------A-T----------PGNNVAAVIC----N-NKMY---
---------YG------------------------ILGDT--------------------------------NY-------------NDPQ-------------VTGEASWLLS
>ref_fungi_KKK15912.1Ruonan312/91-178
DMDVDCDG-LEY--GC-----EG--N-P--D--------------------GQPLTNW--GALDAYQVPFIVIPDKFL-------VA-N------------------PD-------L-L----------PGNNVAAIIC----N-NKIF----
--------YA------------------------ILGDS--------------------------------NH-------------NDPQ-------------VTGEASWLLA
>ref_fungi_RDK39067.1Ruonan362/98-185
DMDVDCDG-IDY--KC-----KG--N-G--D--------------------GLPETNW--GALSAYEVPWIVIPDQFL-------TA-N------------------ED-------L-L----------PGNNVAAVIC----N-
GKMY------------YG------------------------ILGDS--------------------------------NG-------------DDPE-------------VTGEASWLMA
>ref_fungi_PYH97196.1Ruonan341/98-185
DMDVDCDG-KDW--KC-----NS--N-G--D--------------------GQPQTNW--GTLAAYEVPWIVIPDQVL-------TA-N------------------PT-------L-L----------PGNNVAAVIC----N-
GNMF------------YG------------------------ILGDT--------------------------------NG-------------DNPQ-------------ITGEASWVMA
>ref_fungi_KAA8646466.1Ruonan287/81-168
DMDVDCDG-MDF--GC-----KG--N-P--D--------------------GQSITNW--GALSAYDVPFIVIPDDFL-------SA-N------------------RD-------K-M----------PGNNVAAVIC----N-
GNMY------------FG------------------------ILGDS--------------------------------NG-------------DTPL-------------VTGEASWLMA
>ref_fungi_PLB45331.1Ruonan354/92-179
DMDVDCDG-LDY--GC-----KG--N-P--D--------------------GLPKTNW--GALSAYHVPFIVVPDDFM-------VV-N------------------QH-------D-L----------PENNVAAVIC----N-
GKMY------------YG------------------------ILGDS--------------------------------NG-------------DTPL-------------VTGEASWLMA
>ref_fungi_KAB8077979.1Ruonan305/98-185
DMDVDCDG-LNH--DC-----KG--N-P--D--------------------GQGQTNW--GALSAYEVPFIVVPNSFQ-------AA-N------------------SG-------A-L----------PGNNVVAVIC----N-
GKMY------------YG------------------------ILGDT--------------------------------NG-------------NDPE-------------VTGEASWVLA
>ref_fungi_KAE8154996.1Ruonan332/99-186
DMDTDCDG-LNH--GC-----PG--N-P--D--------------------GQPQTNW--GALSAYQVPFIVIPNDFQ-------VA-N------------------SA-------A-I----------PGNNVVAVIC----N-GKMF-
-----------YG------------------------IVGDT--------------------------------NG-------------NSPQ-------------VTGEASWIMA
>ref_fungi_BAE63124.1Ruonan389/98-189
DMDVDCDG-LNH--GC-----KG--N-P--D--------------------GQKETNW--GALSAYEVPFIVIPQEFL-------DA-N------------------KG-------T-L----------KGNAVAAVICATSSN-
GKMF------------YG------------------------IFGDS--------------------------------NG-------------DSPQ-------------VTGEASWLMA
>ref_fungi_KAE8356744.1Ruonan293/99-185
DMDVDCDG-LDH--NC-----EG--N-P--D--------------------GQDKTNW--GALSAYEVPYVVIPEEFL-------QA-N------------------PS-------I-K----------P-NALTAVIC----N-GKMY--
----------YG------------------------ILGDA--------------------------------NG-------------NSPQ-------------VTGEASWLMA
>ref_fungi_EAW13238.1Ruonan390/99-186
DMDVDCDG-LDH--NC-----KG--N-P--D--------------------GQPDTNF--GALAAYEVPFVVIPDRFA-------TT-Y------------------AS-------A-L----------PGNNIVAVIC----D-GKMF-
-----------YG------------------------IFGDT--------------------------------DG-------------DHPQ-------------VIGEASWLMA
>ref_fungi_QZP43727.1Ruonan346/98-185
DMDVDCDG-LDY--KC-----KG--N-P--D--------------------GQPNTNF--GALAAYEVPFIVIPDKFA-------TT-Y------------------ES-------V-L----------PGNNIGAVIC----D-GKMF---
---------YG------------------------IFGDT--------------------------------DG-------------DSPQ-------------VIGEASWLMA
>ref_fungi_OQD80961.1Ruonan457/98-185
DMDVDCDG-IDY--KC-----KG--N-P--D--------------------GQAETNF--GALAAYEVPFIVIPDKFG-------TK-Y------------------AS-------V-L----------PGNNIGAVIC----N-GKMF---
---------YG------------------------IYGDT--------------------------------DG-------------DSPQ-------------VIGEASWLMA
>ref_fungi_OQD94714.1Ruonan357/149-236
DMDVDCDG-LDY--KC-----KG--N-P--D--------------------GLPDTNF--GALAAYEVPFFVIPDRFG-------TK-Y------------------AK-------E-L----------PGNNIGAVIC----D-GKMF--
----------YG------------------------IYGDS--------------------------------DG-------------DSPQ-------------VIGEASWLMA
>ref_fungi_CRG88486.1Ruonan258/97-184
NMNVDCDG-IDY--KC-----KG--N-P--D--------------------GQDTTDN--GELAAYEVPWVVIPEHFQ-------EA-Y------------------SD-------V-L----------PGNNLAAVIC----N-
NKMF------------YG------------------------IYGDS--------------------------------DG-------------DTPE-------------DIGEASWLMA
>ref_fungi_RHZ43460.1Ruonan374/100-187
DMDVDCDG-LDY--QC-----KG--N-G--D--------------------GQDATNF--GALAAYEVPFIVIPQHFY-------KA-Y------------------ES-------Q-L----------TGNNVAAVIC----N-
GKMY------------YG------------------------IFGDT--------------------------------DG-------------DTPE-------------VIGEASWLMA
>ref_fungi_KIN01125.1Ruonan299/96-183
DMDVDCDG-IDY--KC-----KG--N-P--D--------------------GQDQTNF--GALAAYEAPWIVIPDKFA-------SV-N------------------SG-------Q-L----------AGNNVAAVIC----D-GKIF-
-----------YA------------------------VFADT--------------------------------DG-------------DSPE-------------EIGEASWLLA
>ref_fungi_OAQ69235.1Ruonan257/105-192
DMDVDCDG-LDY--KC-----EG--N-P--D--------------------GQDETNF--GALAAYEVPFVVIPDKFA-------SE-H------------------SS-------E-L----------AGNNLVAVIC----G-GKLY-
-----------YG------------------------VFGDT--------------------------------DG-------------DSPE-------------EIGEASWLMA
>ref_fungi_KIM71577.1Ruonan275/108-195
DMDIDCDG-IDF--NC-----KG--N-S--D--------------------GQPQTDY--GALAAYQVPFIVIPGNWV-------NG-H------------------SK-------E-L----------PGNNVAAVIC----G-GKMY-
-----------YG------------------------IFGDT--------------------------------NG-------------ATPE-------------VIGEASYRMG
>ref_fungi_KAF8336588.1Ruonan206/54-141
DMDIDCDG-IDY--RC-----PG--N-T--D--------------------GQPQTTY--GALSAYSVPWIVIPDSFV-------SQ-H------------------SS-------R-L----------PGNNVAAVLC----N-GKLF----
--------YG------------------------ILGDT--------------------------------NG-------------DSPE-------------VTGEASYLLG
>ref_fungi_EJD35896.1Ruonan286/89-178
DLDVDCDG-VDW--QC-----PG--N-E--D--------------------GQPQTSF--GHLSAYRVPFYVVPVTFS-------EQQQ------------------RA-------GNL----------RPNAVGAVIC----N-
GNMY------------YG------------------------IFGDE--------------------------------DG-------------DSPE-------------VIGEASILIA
>ref_fungi_EJD41398.1Ruonan332/92-177
DMDVDCDG-VAW--QC-----PG--N-P--D--------------------GQAQTSF--GAMDATQAPWYVIPEEFY-------QQ-G------------------Y---------L----------QPNALGAVIC----N-
GRMF------------YG------------------------VFADS--------------------------------NG-------------NTPQ-------------IIGEASLILA
>ref_fungi_SJL04887.1Ruonan278/81-167
DMDVDCDG-VDY--KC-----PG--N-G--D--------------------GQDETDF--GALDARSVPWYVLPNKFY-------KA-N------------------KD---------I----------KPNALGAIIC----D-
GKMY------------YG------------------------IFGDS--------------------------------NG-------------DTPE-------------VIGEASLLLA
>ref_fungi_KTB27940.1Ruonan239/92-179

DMDVDCDG-VDY--MC-----PG--N-S--G--------------------GDRDTSF--GHLDASKVPYYVIPARFY-------SQ-Y------------------YP-------K-V----------LPNALGAIIC----N-GKIF---
---------YG-------------------------IMGDT----------------------------------NG-------------ANPQ-------------MIGEASYLLA
>ref_fungi_TFY70230.1Ruonan784/226-312
DMDVDCDG-VDF--QC-----SG--N-P--D--------------------GQSATSF--GALNAAQVPFYVLPLKFT-------DA-H------------------KD---------I----------KPNALGAIIC----N-DKIF-----
-------YG-------------------------IYGDQ----------------------------------NG-------------DDPQ-------------VIGEASIVLA
>ref_fungi_KAF7327226.1Ruonan722/129-216
DMDTDCDG-VSE--NC-----EG--N-A--D--------------------GQNATAF--GALDATRVPYFVLPLNFT-------ME-H------------------RN-------I-L----------KPNALGAIIC----G-QQMV-
-----------YG-------------------------IYGDQ----------------------------------NG-------------DDPQ-------------VIGEASILVG
>ref_fungi_KAF7324972.1Ruonan256/136-219
DMDVDCDG-P---KG--N-R--D--------------------GQSQTSF--GALDATRVPYFVLPERFT-------KE-Y------------------KS-------M-V----------MDNALGAIIC----N-GKMF----
--------YG-------------------------IYGDQ----------------------------------DA-------------DSPQ-------------VIGEASILIA
>ref_fungi_KAF8218012.1Ruonan218/75-158
DMDIDCDG-L---KG--N-Q--D--------------------GQTVTSF--GALDATRVPYFVLPERFT-------TQ-Y------------------KS-------I-L----------KDNALGAIIC----G-SKML--------
----YG-------------------------IYGDQ----------------------------------DA-------------DSPQ-------------VIGEASILIG
>ref_fungi_TFY78632.1Ruonan309/102-188
DMDVDCDG-VDF--QC-----SG--N-P--D--------------------GQSETSF--GRLDAGQVPFYVLPEQFT-------NK-H------------------KD---------I----------KPNALGAIIC----N-NKMF----
--------YG-------------------------IFGDE----------------------------------NG-------------DDPQ-------------LIGEASILLA
>ref_fungi_BAH80469.1Ruonan281/136-221
DMDVDCDG-TDY--QC-----QG--N-D--D--------------------GQSETSY--GALSAKRVPFYVLPQSFV-------DQ-Q------------------K---------I----------KGNSLGAIIC----N-NKMF---
---------YA-------------------------IMGDT----------------------------------NG-------------DSPE-------------VIGEASWLMA
>ref_fungi_KAF9058762.1Ruonan249/104-189
DMDVDCDG-VDY--QC-----KG--N-P--D--------------------GQSETSY--GALAATQVPFYVIPQSFV-------DK-Q------------------K---------I----------KGNSLGAIIC----N-NKMF----
--------YA-------------------------IMGDT----------------------------------NG-------------DSPE-------------VIGEASLLLA
>ref_fungi_THU98230.1Ruonan165/24-110
DMDVDCDG-AE---PN--D-P--S--------------------GQGQTTW--GYLNADQVPFYVLPQSLV-------FDET------------------DG-------EFV----------QPNSLGAIIC----G-GKMF--
----------YA-------------------------IMGDT----------------------------------NG-------------DDVE-------------HIGEASILLA
>ref_fungi_THU98237.1Ruonan352/188-275
DMDVDCDG-SQ--SC-----SN--N-P--D--------------------GQSQTSY--GALNAEKVPFYVIPESFL-------GK-K------------------DG-------GFI----------KPNSLGAIIC----N-GKMF-----
-------YA-------------------------IMGDT----------------------------------NG-------------DDPQ-------------LIGEGSILLA
>ref_fungi_KAA8903987.1Ruonan280/85-183
DMDVDCDG-SS--NC-----EGK--S-K--D--------------------YQSGTAFD--------------DVLAGQNY------------GI------ASLDAEVHPYVVLGTCSA-----DVA------------------------------KVV----------KPLSVVAVVC----
N-RKLH------------YA-------------------------VWGDT----------------------------------NG------------CDDDN-------------FTGEASLALA
>ref_fungi_TGZ83149.1Ruonan298/77-185
DMDIDCDG-AA--NC-----GSL--KKN-S--D--------------------FQPETAFD--------------DTLAELGY------------AIP----GKSLNASVIPYAVLGTCDV-----PVAKN----------------KK-EK-------GPV-----------
PELGVVAVVC----G-DKLR------------YA-------------------------VFGDT----------------------------------NG------------CDDDN-------------FTGEASIALA
>ref_fungi_KAA8902930.1Ruonan339/76-177
DMDIDCDG-AH--KC-----TG--D-G--S--------------------FQSGTAFD--------------DILQSQGY------------GI------ESLDASKHTFVVLGTCDV-----AVDT------------------L-TG-------GPI----------QPLSLVAVVC----
N-NQLH------------YA-------------------------VFGDT----------------------------------NG------------CDDDN-------------FTGEASLSLG
>ref_fungi_KIM76821.1Ruonan236/69-170
DMDVDCDG-KE--SC-----SH--D-G--D--------------------HQSGTAFD--------------SILSRGRY------------GI------SKLDPLIHSYSVLGTCHV-----PVDK------------------P-SG-------GPV----------PPLSTVAVVC----N-
NQLF------------YS-------------------------VFGDT----------------------------------NG------------CDDAD-------------FTGEASYALA
>ref_fungi_EPS45085.1Ruonan289/75-172
DMDIDCDG-AH--NC-----GGL--S-G--D--------------------FQAQTSFD--------------DIL-KKSY------------GI------KTLDASIHQFSVLGTCHV-----SMT------------------------------GTI----------EPLALVAVVC----G-
GNLF------------YS-------------------------VWGDT----------------------------------NG------------CDGDD-------------YTGEASISLA
>ref_fungi_RVD82095.1Ruonan516/190-288
DMDVDCDG-AS--NC-----GGL--S-G--D--------------------WQAGTSFD--------------DILASKKY------------GI------KTLDASVHQFSVLGTCNL-----NLE------------------------------GLI----------SPLALVAVVC----N-
NQLF------------YS-------------------------VWGDT----------------------------------NG------------CDDNN-------------FTGEASISLA
>ref_fungi_CDR46339.1Ruonan468/102-192
NMDVDCDG---------------------------SSK----------KG-P-C-----SN--D-E--S--------------------FQGQTAFQ--------------D------------------KSG------KDIDA---HYVVIDQDK-------------------------DFDP-TK-------FGV----------QPLSVVAVVC----G-
GKLT------------FG-------------------------IWADT----------------------------------NA------------LGD-----------------MGEVSVYLA
>ref_fungi_KPV73414.1Ruonan357/89-183
NMDIDCDG---------------------------SDG----------KG-EIC-----NG--D-G--S--------------------YQSSTTFQ--------------N------------------DDG------KHIDAQAIQYVVIDQDD-------------------------DFDP-VK-------FGI----------QPLSVVAVVC----G-
GKLT------------FG-------------------------VWADT----------------------------------NA------------LGS-----------------MGEASVYLA
>ref_fungi_KFX85809.1Ruonan242/76-179
NMDIDCDG-AN-------------------------RT----------GG--KC-----SN--D-P--T--------------------GQGQTAFQ--------------S-EIQ-ELN----------VGI------DDLDSNLHPYVVFGNE----------------------GSSPEFDP-RQ-------YGM-----------
EPLSVMAVVC----N-NQVF------------YG-------------------------VWGDT----------------------------------NG-------------ET-----------------SVGEASISLA
>ref_fungi_KXG51763.1Ruonan319/73-168
DMDIDCDG-AN-------------------------RL----------EG--DC-----YN--D-G--S--------------------GQSQTAFM--------------D-ELE---------------GI------DDLDAGIHPYVVFGTG--------------------------DYDP-RD-------DGM----------KALSVMAVVC----
G-GKLH------------YG-------------------------IWGDT----------------------------------NG-------------HT-----------------ATGEASLSMA
>ref_fungi_OQE35243.1Ruonan326/76-173
DMDIDCDG-AN-------------------------RL----------NG--DC-----GD--D-N--S--------------------GQSQTAFM--------------G-DLK-PY------------HI------DDLDAGIHPYVVFGTK--------------------------GYDP-EE-------DGM----------KPLSVMAVVC--
--G-GKLH------------YA-------------------------IWGDT----------------------------------NG-------------HT-----------------STGEASLSLG
>ref_fungi_QRD94823.1Ruonan282/115-219
DMDIDCDG-AN-------------------------RH----------AG--KC-----SN--D-H--S--------------------GQGETRWK--------------D-EVQ-KL------------GI------DDLDANIHPYVVFGNEN--------DD-----------GDDPEFDP-RK-------HGM-----------
EPLSVMAVVC----N-KKLF------------YG-------------------------IWGDT----------------------------------NG-------------HT-----------------ATGEASLSMA
>ref_fungi_OGE54044.1Ruonan237/77-174
NMDVDCDG-LN-------------------------DK----------GG--DC-----GS--D-P--T--------------------GQGETAFK--------------D-QLS-QF------------GI------EDLDANLHPYVVFGNT----------------------G----FDP-QQ-------YGM----------EPLSVMAVVC--
--N-NQVL------------YG-------------------------IWGDT----------------------------------NG-------------AD-----------------STGEASISLA
>ref_fungi_KAA8645350.1Ruonan235/76-173
NMDVDCDG-VN-------------------------NS----------GG--AC-----GD--D-P--T--------------------GQNQTSFK--------------G-LLN-QY------------GI------DELDANIHPYVVFGNE--------------------------AFNP-QE-------YGM----------EPLSVMAVVC--
--G-DQLH------------YG-------------------------IWGDT----------------------------------ND-------------ED-----------------STGEASISLA
>ref_fungi_KKK17910.1Ruonan233/73-170
DMDIDCDG-TD-------------------------DS----------GG--DC-----GN--D-P--S--------------------GQGQTSFQ--------------S-QLG-DY------------GI------SDLNAKVHPYVVFGNT--------------------------NFDP-QE-------YGM----------EPLSVMAVVC---
-G-GQLH------------YG-------------------------IWGDT----------------------------------NG-------------GD-----------------LTGEASISLA
>ref_fungi_OJJ97649.1Ruonan232/73-170
NMDVDCDG-VS-------------------------PT----------SG--GC-----RN--D-P--S--------------------KQSQTAFH--------------D-QFR-QY------------DI------GDLSAFVHPYVVFGNT--------------------------NFDP-QQ-------YGM----------QPLSVMAVVC---
-G-GQLH------------YG-------------------------IWGDT----------------------------------NG-------------GS-----------------KTGEASLAMA
>ref_fungi_RAL13410.1Ruonan232/73-170
DMDVDCDG-AS-------------------------ST----------SG--GC-----GN--D-P--S--------------------NQGQTTFQ--------------D-QVR-PF------------GI------NNLNANIHPYVVFGNT--------------------------NFSP-QQ-------YGV----------QPLSVMVVVC----
G-EQLF------------YG-------------------------IWGDT----------------------------------NG-------------GS-----------------KTGEASISLA
>ref_fungi_PWY87547.1Ruonan240/80-177
NMDVDCDG-AH-------------------------AS----------GG--DC-----GD--D-P--S--------------------IQGQTSFQ--------------D-QVV-QY------------GI------SDLDANVHPYVVFGNT--------------------------NFDP-QS-------SGM----------QPLSVMAVVC--
--G-GQLY------------YG-------------------------VWGDM----------------------------------NG-------------GG-----------------VTGEASIALA
>ref_fungi_RDK47088.1Ruonan254/84-182
NMDIDCDG-AN-------------------------SH----------GG--NC-----GY--D-E--S--------------------IQDQTSFK--------------DLVVS-QY------------GI------PDLDANIHPYVVFGNT--------------------------DYDP-QR-------DGM----------WPLSVMVVVC--
--G-NQLF------------YG-------------------------IWGDT----------------------------------AG-------------GS-----------------WTGEASIALA
>ref_fungi_PWY93418.1Ruonan239/79-176
NMDIDCDG-TN-------------------------PN----------SG--NC-----GN--D-P--S--------------------IQPQTTFQ--------------D-QVS-QY------------GI------SDLDANVHPYVVFGNT--------------------------DFDP-QG-------SGM----------EPLSVMAVVC----
G-GEVV------------YG-------------------------IWGDT----------------------------------NG-------------GD-----------------ATGEASIALA
>ref_fungi_RJE19078.1Ruonan241/78-179
DMDIDCDG-SN-------------------------IH----------GG--DC-----GD--D-P--T--------------------GQGETAFK--------------S-QVQ-QY------------GI------DDLDATVHPYVVFGNE----------------------GGDVEFDP-QE-------YGM-----------
QPLGIMAVVC----D-GKLI------------YG-------------------------IWGDT----------------------------------NG-------------GT-----------------STGESSISLA
>ref_fungi_TQB70058.1Ruonan244/80-181
NMDIDCDG-SN-------------------------MY----------GG--KC-----SN--D-P--T--------------------GQPETAFK--------------D-QVS-QY------------GI------SDLDATIHPYVVFGNE----------------------GAVPNFDP-QE-------YGM-----------
KPLSVMAVVC----D-GQVF------------YG-------------------------VWGDT----------------------------------NG-------------LT-----------------STGEASISLA
>ref_fungi_EGU78186.1Ruonan260/103-198
NMDIDCDG-AN-------------------------NH----------AG--AC-----SN--D-P--T--------------------GQGETAFK--------------D-TVN-QY------------GI------SDLDANVHPYVVFGNE----------------------GASPSFDP-QQ-------HGL----------KPLSVMAV---
--------H------------YG-------------------------IWGDT----------------------------------NG-------------GT-----------------STGEASISLA
>ref_fungi_KPA35701.1Ruonan234/72-173
NMDIDCDG-AN-------------------------NH----------AG--AC-----AN--D-P--T--------------------GQGVTAFR--------------D-TVA-GY------------GI------PDLDANLHPYVVFGND----------------------GDSPSFDP-QT-------YGM-----------
KPLSVMAIIC----D-GKLH------------YG-------------------------IWGDV----------------------------------NG-------------GT-----------------STGEASISLA
>ref_fungi_CRL27399.1Ruonan285/76-177
DMDVDCDG-AN-------------------------NS----------EG--GC-----SN--D-P--S--------------------GLGVTAFQ--------------D-EVK-HF------------GI------KDLDANIHPYVVFGNE----------------------EHKPSFMP-DK-------HGM-----------
EHLSVMAIVC----G-GQLH------------YG-------------------------IWGDT----------------------------------NG-------------GT-----------------STGEASISLA
>ref_fungi_OQE00038.1Ruonan296/75-176
DMDIDCDG-VN-------------------------RS----------AG--DC-----AN--D-G--S--------------------GQDETAFK--------------S-EVA-DY------------GI------EDLDANLHPYVVFGNE----------------------GSSPSFMP-DE-------HGM-----------
EHLSVMAVVC----G-GKLH------------YG-------------------------IWGDT----------------------------------NG-------------AT-----------------STGEASISLA
>ref_fungi_CEJ80605.1Ruonan233/70-171
NMDVDCDG-AN-------------------------NS----------GG--DC-----AN--D-P--S--------------------GQGETAFK--------------D-TVK-GY------------GI------GDLDANIHTYIVFGNE----------------------GGKTTFKP-QD-------HGI-----------
KPLSLMAVVC----G-GKLH------------YG-------------------------IWGDT----------------------------------NG-------------GT-----------------STGEASIAMA
>ref_fungi_EPS31072.1Ruonan380/77-178
DMDIDCDG-AN-------------------------NS----------AG--AC-----SN--D-P--S--------------------GQGQTAFK--------------D-DVK-EF------------GI------SDLNANVHPYVVFGNE----------------------DGNPKFDP-KK-------YGM-----------
EPLSVMAVVC----N-GQLH------------YG-------------------------IWGDT----------------------------------NG-------------GH-----------------VTGESSLSMA
>ref_fungi_KAE8376156.1Ruonan239/75-176
NMDIDCDG-AN-------------------------NT----------AG--KC-----AN--D-K--S--------------------GQGITAFQ--------------D-EVK-KF------------GI------SDLDANLHPYIVFGNE----------------------DHSPHFNP-QE-------YGM----------EPLSVMAVVC-
---K-NQVH------------YG-------------------------IWGDT----------------------------------NG-------------GT-----------------STGESSLAMA
>ref_fungi_AQA29344.1Ruonan262/85-194
DMDIDCDG-AQ--AC-----PD--D-P--T--------------------FQGQTSFK-----------------TAYGGYQGVQELSRAAGAYV------SDLNATRHPYVVFGNEG--------SK---------------PTFNP-KD-------YGM-----------
VPLSVMAVVC----N-GQVH------------YG-------------------------VWGDT----------------------------------NG-------------GT-----------------STGEASLAMG
>ref_fungi_SMR60234.1Ruonan238/74-175
NMDIDCDG-AN-------------------------RS----------AG--RC-----SN--D-P--T--------------------GQGETAFK--------------D-TVK-QY------------GI------SDLDANKHPYVVFGNS----------------------GSNPSFNP-ED-------EHM-----------
HPLSVMAVVC----N-NQVH------------YG-------------------------VWGDT----------------------------------NG-------------GT-----------------STGEASLALA
>ref_fungi_KFG81970.1Ruonan246/68-169
DMDIDCDG-AN-------------------------NS----------TG--DC-----AN--D-P--T--------------------GQGQTAFM--------------D-EVS-QF------------GI------PDLDAHIHPYVVFGNA----------------------EGSPTFVP-QD-------HGM-----------
RPLSVMAVVC----D-NQLL------------YG-------------------------VWGDV----------------------------------NG-------------GN-----------------LTGEASISLG
>ref_fungi_OAA48341.1Ruonan234/70-171
DMDIDCDG-VN-------------------------RS----------AG--AC-----AN--D-Q--T--------------------GQDQTAFM--------------D-TVK-TF------------GI------PDLDANVHPYVVFGNA----------------------EAVPSFVP-QD-------HGI-----------
KPLSVMAVIC----N-DQVH------------YG-------------------------VWGDV----------------------------------NG-------------GV-----------------LTGEASLSMA
>ref_fungi_CEJ90007.1Ruonan320/68-169
NMDTDCDG-LD-------------------------NT----------AG--DC-----GD--D-Q--T--------------------GQSQTAFQ--------------G-LAQ-SY------------GI------EDLNANIHTYVVFGNE----------------------GGNPSFNP-QN-------HGI-----------
KPLSVMAVVC----N-NQVY------------YG-------------------------VWGDT----------------------------------NG-------------GT-----------------TTGEAAISLG
>ref_fungi_PTB45945.1Ruonan324/69-167
NMDVDCDG-SD-------------------------DK----------AG--DC-----SN--D-P--T--------------------GYGETAFK--------------D-TVQ-SY------------GI------SDLNANIHPYVVFNEP--------------------------PFFNP-EQ-------YGM----------KPLSVMAVVC---
-N-NQVW------------YG-------------------------IWGDT----------------------------------NT-------------EP-----------------TTGEAAISLA
>ref_fungi_PTB41840.1Ruonan235/70-171
NMDIDCDG-AN-------------------------NS----------AG--AC-----SN--D-P--S--------------------GQGETAFK--------------D-TVQ-SF------------GI------DDLDANIHPYVVFGNE----------------------GGNPSFSP-EQ-------FGM----------QPLSIMAVVC---
-N-DQVF------------YG-------------------------IWGDT----------------------------------NG-------------FT-----------------STGESAISMG
>ref_fungi_KHN95581.1Ruonan236/69-172
NMDIDCDG-ARY----W-------------------------------SG--EC-----SN--D-G--DI-------------------YGLGYTAFV--------------D-TVR-TY------------GI------RDLDTNVHPYVVFGNEG--------SN---------------PSFDP-RS-------MGM-----------
QPLSVMAVVC----N-GQLF------------YA-------------------------VWGDT----------------------------------ND------------------FT------------STGEASLALG

>ref_fungi_KAF4464478.1Ruonan234/73-170
NMDIDCDG-AN-------------------------NA-----------AG--DC-----AN--D-P--S--------------------GQGQTSFV---------------D-TVK-KF------------GI------KDLDANLHSYVVFGTN---------------------------SFDP-QK-------HGL-----------KPLSVMAVVC---
-N-NQVF------------YG------------------------VWGDT----------------------------------NG-------------FR----------------STGEASISLA
>ref_fungi_OAQ88808.1Ruonan261/96-197
NLDIDCDG-AN-------------------------NS-----------AG--DC-----AN--D-P--S--------------------GQGQTSFV---------------D-TVK-NY------------GI------SDLDANLHPYVVFGNE----------------------GASPSFNP-QD-------HGI-----------KPLSVMAVVC-
---N-NQVF------------YG------------------------VWGDT----------------------------------NG-------------FT----------------STGEASLALG
>ref_fungi_OXV08208.1Ruonan203/5-106
GMNVDCDG-AN-------------------------NS-----------GG--KC-----TN--D-P--S--------------------GQGQTAFK---------------D-TVK-SY------------GI------PDLDANLHSYVVFGNE----------------------GAIPTFNP-KT-------AGM-----------
EPLSVMAVVC----G-DNLI------------YG------------------------VWGDT----------------------------------NG-------------AD----------------STGEVSISLA
>ref_fungi_GAD97933.1Ruonan979/549-650
NMDVDCDG-AN-------------------------NT-----------AG--KC-----NN--D-P--S--------------------GQGETTFR---------------H-IVK-SY------------GI------PDLNANIHSYIVFGNE----------------------GRIPSFNP-QA-------YGM-----------RPLSVMAVIC---
-N-NQLF------------YG------------------------VWGDT----------------------------------NG-------------GV----------------DTGEASISLA
>ref_fungi_RWQ92488.1Ruonan270/88-189
DMDVDCDG-AN-------------------------NT-----------VG--RC-----GN--D-P--S--------------------GQGKTAFK---------------N-IAQ-SY------------GI------PDLNANIHSYIVFGNE----------------------GAVPSFNP-RA-------HGM-----------RPLSVMAVIC--
--N-NQLF------------YG------------------------VWGDT----------------------------------NG-------------GV----------------DTGEASISLA
>ref_fungi_KKA18539.1Ruonan254/88-189
DMDVDCDG-AN-------------------------NK-----------GG--KC-----SN--D-P--S--------------------GQSQTAFQ---------------D-IVK-TY------------GI------KDLNANIHPYVVFGNE----------------------DSSPSFKP-QS-------VGM-----------
KPLSVMAVVC----N-NNLF------------YG------------------------IWGDT----------------------------------NG-------------GT----------------DTGEAAISLA
>ref_fungi_KXS96242.1Ruonan247/80-184
NLDIDCDG-AS-------------------------SG-----------TG--AC-----AD--D-P--T--------------------GQGITAFQ---------------Y-LLP-QY------------GI------NDLDAQIHPYVVFGND--------GDC------------DDCSYIDP-TE-------HGM-----------
QPLSVMAIVC----N-GQLH------------YG------------------------VWGDV----------------------------------NG-------------GQ----------------DTGETSLALG
>ref_fungi_KAB2580152.1Ruonan244/82-182
DMDVDCDG-AN-------------------------LG-----------AG--DC-----AN--D-P--T--------------------GQSQTAFQ---------------W-IVA-DK-----------YGI------DDLDANVHSYVVFGND-------------------------DPAFDP-QQ-------HGM-----------
EPLSVMAVVC----G-GQLH------------YG------------------------VWGDT----------------------------------NG-------------AN----------------TVGETSLALA
>ref_fungi_KAF2123123.1Ruonan253/76-178
DMDIDCDG-LN-------------------------AD-----------QG--DC-----HN--D-P--T--------------------GQSITAFQ---------------D-EVS-QF------------GI------KDLDAHIHTYVVFGNE----------------------GDNPSFSPTDA-------ADI-----------QHLSVVAVLC--
--N-NQLL------------YG------------------------VWGDT----------------------------------NG-------------AT----------------SVGESSLALG
>ref_fungi_KAF2727200.1Ruonan260/77-185
DMDIDCDG-AS-------------------------VQ-----------QG--AC-----SN--D-G--T--------------------GQGITAFQ---------------D-QVK-KF------------GI------NDLDSQIHTFVVLGNDNS-----ASEG------------NGGTPFDPAQS-------ANI-----------
KPLSVVAVVC----N-NQLL------------YG------------------------VWGDV----------------------------------NG-------------GI----------------LTGESSVALA
>ref_fungi_ORY14365.1Ruonan275/77-184
DMDIDCDG-LN-------------------------AG-----------EG--DC-----FN--D-P--T--------------------GQSMTAFQ---------------S-DAQ-KF------------GI------DDLDAHIHTYVVLGNDNS-----ASEG------------NGGEKFDP-KS-------AGI-----------
EPLSVVAVVC----G-GKMF------------YG------------------------VWGDI----------------------------------NG-------------GV----------------STGESSLALG
>ref_fungi_KAF2197967.1Ruonan276/72-179
DMDIDCDG-AN-------------------------NG-----------EG--RC-----SN--D-P--S--------------------GQGQTAFK---------------D-TVK-KY------------GI------SDLDSHIHTFVVLGNDNS-----REEG------------NGGQAYDP-QD-------DNI-----------
KPLSVVAVVC----D-GKMF------------YG------------------------VWGDV----------------------------------NG-------------GI----------------STGEASLSMG
>ref_fungi_KAF1967217.1Ruonan260/75-186
DVDIDCDG-AG-------------------------IG-----------TG--DC-----GD--D-P--S--------------------GQSMTAFK---------------D-LVQ-EYS---------NGAV------DDLNTHIHNFVVLGNDNS-----ADEG------------DGGKSFDPTKD-------ADI-----------
QPLSVVAVVC----G-DKLV------------YG------------------------VWGDV----------------------------------NG-------------GK----------------VTGEASLSLA
>ref_fungi_TQB76246.1Ruonan237/78-174
NMDIDCDG-SE---PC-----PN--D-P--S--------------------GQDITAFQ---------------D-RVT-RY------------GI------NDLNANIHPYVVFGND----------E------------GE--TFYP-DQ-------HGM-----------KPLSVMAVVC----
N-NQVF------------YG------------------------IWGDT----------------------------------NG-------------GK----------------STGEASIALA
>ref_plant_XP_023908599.1Ruonan353/189-290
NMDIDCDG-AN-------------------------DK-----------AG--AC-----AN--D-P--S--------------------GQSITAFQ---------------D-QVS-QF------------GI------SDLDANVHPYVVFGNS----------------------GSSPTFDP-TS-------QGM-----------QPLSLMAVVC--
--N-NQLF------------YG------------------------VWGDT----------------------------------NG-------------GT----------------STGEASIAMA
>ref_fungi_RPB15530.1Ruonan204/76-167
DMDVDCDG-AN-------------------------NA-----------HG--AC-----EN--D-P--T--------------------GQSITAFQ---------------D-EVS-QF------------GI------ADLDANLHSYVVLGTG---------------------------GFDP-RK-------EGV-----------SSLSVVAV--------
---F------------YG------------------------VWGDS----------------------------------NA-------------DD----------------LVGEASLSLA
>ref_fungi_RMJ23475.1Ruonan273/81-176
DMDVDCDG---------------------------IDRS-----------AG--SC-----VD--D-T--T--------------------GQDETSFR---------------K-TVT-KY------------NI------PDLNSSVHSYVVFGNQ----------------------DAEPTFDP-WR-------AGM-----------EPLSVMAI-----
------F------------YG------------------------VWGDK----------------------------------NG------------------GV------------YTGEASISLA
>ref_fungi_EAT78515.2Ruonan193/19-130
DMDIDCDG-----A---------------------GKG-----------QG--GC-----SD--D-P--T--------------------GLGDTSFK---------------S-QLQ-DLT----------KGAV------KDLNTNKHTFVVLSNFII-----NDAQ-----------GDSRKPFDI-ET-------AGV-----------
EPLSIVAVIC----G-NQMF------------YG------------------------VYGDQ----------------------------------NG------------------GV------------VTGEASLSLG
>ref_fungi_CUS09169.1Ruonan305/77-178
DMDIDCDG-ANR-------------------------S-----------AG--KC-----LN--D-P--T--------------------GQGQTAFK---------------D-RVA-KFG------------I------EDLDSNKHSYIVFGNQK--------YS---------------PSFEP-TQ-------YGV-----------PELAVAVVVC-
---G-GQFF------------YA------------------------VWGDT----------------------------------NG------------------GT------------LVGEASISLA
>ref_fungi_RPB07873.1Ruonan236/77-174
DMDIDCDG-AN-------------------------QG-----------YG--KC-----AN--D-P--T--------------------GQGMTSFV---------------G-DVV-KH------------GL------SDLDSNTHSFVVLGNH---------------------------GFDP-QS-------LGI-----------KPLSLVAVIC----
G-GQLF------------YA------------------------IWGDS----------------------------------NA-------------TD----------------KVGEASISLA
>ref_fungi_EAS28569.3Ruonan247/82-183
DMDVDCDG-ANR-------------------------S-----------AG--DC-----KN--D-P--S--------------------GQDQTAFK---------------D-EVK-KYG------------I------PDLDSNKHGYVVLGNEG--------SS---------------PSFNP-AE-------YGI-----------
KPLSVVAVVC----N-NRLF------------YG------------------------VWGDT----------------------------------NG------------------GT------------LVGETSVSLA
>ref_fungi_EGD90700.2Ruonan242/71-172
DMDVDCDG-IKR-------------------------S-----------KG--AC-----AN--D-P--S--------------------GQDQTAFK---------------H-EVP-RYG------------I------EDLDSNKHAYVVLGTQG--------SE---------------PSYMP-SA-------SNV-----------
ESLSVVAVVC----N-GTLF------------YG------------------------VWGDT----------------------------------NG------------------GT------------DVGEASVSLA
>ref_fungi_EEQ30784.1Ruonan282/83-184
DADVDCDG-ANR-------------------------S-----------KG--DC-----AN--D-L--T--------------------GQGQTTFK---------------D-ELA-SYG------------I------DDLDSNLHSYVVFGNEG--------KK---------------PSYLP-SD-------SGI-----------EPLSVIAVVC---
-N-NRLF------------YG------------------------IWGDT----------------------------------NG------------------NT------------DTGEVSISLA
>ref_fungi_KAF3483974.1Ruonan288/123-224
DMDVDCDG-ANR-------------------------S-----------KG--DC-----AN--D-P--S--------------------GQGQTSFK---------------D-EVA-KYG------------I------EDLDSNKHSYVVLGNEG--------KS---------------PRYLP-SA-------SGV-----------
ESLSVVAVVC----N-NKLF------------YG------------------------VWGDT----------------------------------NG------------------GT------------DTGEVSISLA
>ref_fungi_KHO00593.1Ruonan241/68-168
NLDIDCDG-AN-------------------------NY-----------AG--DC-----AN--D-P--T--------------------GQSQTAFK---------------A-NVS-QY------------GI------SDLDANIHSYVVLGNE-----------------------GPPAFDP-QS-------RGI-----------QPLSVVAVVC---
-G-EQLF------------FG------------------------VWGDV----------------------------------NG-------------GN----------------LTGETSIALG
>ref_fungi_CZT20497.1Ruonan256/80-195
NMDIDCDG-DQS-----------------------DHG-----------DG--RC-----GK--S-T--D--------------------TQSTTAFK---------------D-QVQ-QYSKQ------TGTEV------SDVNANYIPYVVFGNEG--------SK------------NGYTNFNP-QD-------HGI-----------
QPLSVMAVVC----G-DQLV------------YG------------------------VWADT----------------------------------NG-------------DDGP-P------------LVGEASISLA
>ref_fungi_PBP22935.1Ruonan302/89-206
DMDVDCDG-TQR----------------------PRLGD-----------DG--RC-----GT--S-S--D--------------------MQSMTSFR---------------G-DVG-SYG----------RSI------QDLNAFVHPYVVFGNSA--------SRDL----------PGYVQYDP-RG-------DGV-----------
HPLSVVATVCG---N-GKMF------------YG------------------------IWGDV----------------------------------NG-------------DDAPKA------------IIGEASISMA
>ref_fungi_RDL39916.1Ruonan326/82-196
DMDIDCDG-LQG-G---------------------PGD-----------DG--RC-----GS--S-G--D--------------------TQSQTSFK---------------D-KIA-GYG----------VSGV------KDLNAFVHPYVVFGNVG--------SK------------AGFTNYDP-RA-------NGI-----------
EPLSLMAVVC----G-GKMF------------YG------------------------IWGDE----------------------------------NG-------------DDGPKA------------VIGEASISMA
>ref_fungi_RFU29037.1Ruonan354/81-194
DMDIDCDG-LQK-G---------------------PGD-----------DG--RC-----GS--S-G--D--------------------TQDQTSFE---------------D-TVA-SYS----------KGV------DDLNAFVHPYVVFGNVG--------SK------------SGFVNFDP-RS-------VGV-----------
QPLSLMAVIC----S-GKMY------------YG------------------------IWGDE----------------------------------NG-------------DDGDKS------------MIGEASISIA
>ref_fungi_TVY59621.1Ruonan306/82-192
DMDIDCDG-VQG-G---------------------KGD-----------DG--RC-----GT--S-S--D--------------------TQSQTSFE---------------D-TVA-SYN----------KGV------SDLNAFVHPYVVFGNVG--------SK------------GVF---DP-QS-------KGV-----------
EPLSLMAVVC----G-GKMF------------YG------------------------IWGDE----------------------------------NG-------------DDGPKA------------VIGEASISMA
>ref_fungi_EKD17627.1Ruonan357/83-197
DMDIDCDG-IQN-G---------------------PGN-----------DG--RC-----GS--S-D--D--------------------TQAHTSFE---------------D-TVA-GYN----------KGV------SGLNAFIHTYVVLGNVG--------TN------------AGFVNYDP-RA-------NGV-----------
RELSLVAVVC----GNGKMF------------YG------------------------IWGDE----------------------------------NG-------------DDGPRA------------VIGEASISMA
>ref_fungi_CZT51685.1Ruonan355/83-197
DMDIDCDG-IQN-G---------------------PAS-----------DG--RC-----GK--S-S--D--------------------TQAHTSFE---------------D-TVK-GYN----------KGA------QGLDAYIHTYVVFGNVG--------SN------------AGFVNYDP-RK-------DGV-----------
RELGLIAAVC----GNGKMF------------YG------------------------VWGDE----------------------------------NG-------------DDGPRA------------VIGEASISMA
>ref_fungi_PVH74851.1Ruonan344/86-199
DMDIDCDG-IQH-G---------------------PAD-----------DG--RC-----GS--S-S--D--------------------TQSHTSFE---------------D-TVA-SYN----------KGV------SGLDAFIHPYVVFGNVG--------TN------------PGFVNYDP-RK-------DGV-----------
RELSLMAVVC----G-GKMF------------YG------------------------VWGDE----------------------------------NG-------------DDGPRA------------VIGEASISMA
>ref_fungi_KUJ19604.1Ruonan352/70-183
DMDIDCDG-LQN-G---------------------PGD-----------DG--RC-----GS--S-T--D--------------------TQSQSSFE---------------D-IIS-GYG----------QGV------GELNAFVHPYVVFGNEG--------SS------------GGYQNFNP-QD-------YGV-----------
KPLSLMAVVC----G-GQMF------------YG------------------------IWGDE----------------------------------NG-------------DDGPKA------------MVGEASISMA
>ref_fungi_RGP58888.1Ruonan256/81-194
DMDVDCDG-VQH-G---------------------AGD-----------DG--RC-----GS--S-S--D--------------------TQSQTSFQ---------------D-TVR-SYG----------KGI------KDLNAFVHPYVVFGNEG--------QL------------SGYKNFHP-QE-------HGI-----------
EPLSLMAVVC----G-DKLI------------YG------------------------IWGDT----------------------------------NG-------------DDYPKA------------LVGEASISVA
>ref_fungi_ETS80470.1Ruonan301/80-193
NMDIDCDG-KQH-G---------------------PGN-----------DG--RC-----GY--S-A--D--------------------TQSQTSFQ---------------E-IVA-GYE----------RGI------NDLNPYVHPYVVFGNVG--------TK------------SGYTNFDP-QS-------HGI-----------
EPLSLMAVVC----S-DKLI------------YG------------------------IWGDE----------------------------------NG-------------DDGDLA------------LVGEASISLA
>ref_fungi_AEO60909.1Ruonan312/80-193
DMDIDCDG-EQN-G---------------------KGD-----------DG--RC-----GN--S-Q--D--------------------TQAITSFQ---------------D-IVE-GYK----------KGV------KDLNAYVHPYVVFGNTG--------SK------------SGWKTFDP-QK-------YGI-----------
KPLSVMAVVC----N-NKLI------------YG------------------------VWGDE----------------------------------NG-------------DDGDKP------------MVGEASISLA
>ref_fungi_EGS21767.1Ruonan468/83-196
DMDIDCDG-EQN-G---------------------IGD-----------DG--RC-----NF--D-K--S--------------------YQPITSFQ---------------Y-IVK-KYN----------KGI------NDLNAYVHPYVVFGNSG--------KK------------SGWNTFDP-RA-------YGV-----------
EPLSIMAVVC----N-NKLI------------YG------------------------VWGDT----------------------------------NG-------------DGGKHA------------VVGEASLSLA
>ref_fungi_TPX11611.1Ruonan293/79-189
NMDIDCDG-DRS-G---------------------PGN-----------DG--RC-----NG--D-G--T--------------------SQDITSFQ---------------Y-IVK-GYN----------KGI------NDLNPFVHPYVVFGNVGQ------GNQ-----------------FDP-QA-------HGI-----------
KPLSVMAVVC----G-DRLI------------YG------------------------VWGDE----------------------------------NG-------------NDGPHP------------VVGEASISLA
>ref_fungi_KAF3765643.1Ruonan313/84-203
DMDIDCDG-NQD-----EP---------------SSSAN-----------DG--RC-----GK--S-S--D--------------------TQSDTSYE---------------D-TVK-SYG----------KGI------SNLNPYIHTYVVFGNSND------GNT------------AGYKTFDP-TK-------YGI-----------
EPLSLMAVVC----N-KQVI------------FG------------------------IWGDT----------------------------------NG-------------DDDGSKA------------MVGEAAISTG
>ref_fungi_OAA64764.1Ruonan301/80-193
NMDIDCDG-VQH-G---------------------PAD-----------DG--RC-----QS--S-D--D--------------------TQVQTSFK---------------S-IVR-GYG----------VGV------DDLDANIHPYVVFGNEG--------SR------------SNYPNFDP-KK-------YGV-----------
EPLSVMAVVC----G-NKLI------------YG------------------------IWGDT----------------------------------NG-------------DDGPEA------------MVGEASISLA
>ref_fungi_CEJ80614.1Ruonan337/79-192
NMDIDCDG-IQG-S---------------------AAD-----------DG--RC-----GK--S-T--D--------------------TQSQTSFA---------------G-TVR-SYG----------TSQ------KDLDANIHPYVVFGNEG--------SR------------AGYTTFNP-QS-------YGI-----------
KPLSVMAVVC----N-NKLI------------YG------------------------VWGDE----------------------------------NG-------------DDDAEA------------MIGEASISLA
>ref_fungi_KAG6258325.1Ruonan313/82-195
NMDIDCDG-IQL-S---------------------PAD-----------DG--RC-----GS--S-T--D--------------------MQSVTSFA---------------D-ILR-SYS----------TNA------RDLDANIHPYVVFGNKG--------TR------------EGYKTFDP-RK-------YGV-----------
EPLSVMAVVC----N-NKLI------------YG------------------------VWGDE----------------------------------HG-------------DDDPQA------------MVGEASISLA
>ref_fungi_OIW23025.1Ruonan302/81-194
NMDIDCDG-AQG-G---------------------PAD-----------DG--RC-----GS--S-G--D--------------------TQSITSFA---------------D-TVA-GYG----------KGI------SDLNANVHPYVVFGNAG--------SK------------AGYKTFDP-QA-------YGV-----------
RPLSVMAVVC----G-SKLI------------YG------------------------VWADE----------------------------------NG-------------DDGSHP------------MVGEASISLA
>ref_fungi_PKS06105.1Ruonan289/81-194
NMDVDCDG-AQG-G---------------------PAN-----------DG--RC-----AS--S-R--D--------------------TQAVSSFQ---------------S-TVA-SYG----------RGI------RDLDANIHPYIVFGNEG--------SK------------PGWKTFDP-QE-------VGI-----------
QPLSLMAVVC----G-DKLI------------YG------------------------VWGDT----------------------------------NG-------------DDGPHP------------MVGEAAISVA
>ref_fungi_OTA95616.1Ruonan256/80-193
NMDIDCDG-TQG-S---------------------AAD-----------DG--RC-----GN--S-G--D--------------------TQSITSFA---------------D-TVR-GYG----------KGV------RDLDANIHPYVVFGNVG--------SK------------PGFTEFDP-QE-------HGV-----------
EPLSVMAVVC----G-DKLI------------YG------------------------VWGDE----------------------------------NG-------------DDGEKS------------VVGEASISLA
>ref_fungi_TRX96945.1Ruonan296/81-194

DMDIDCDG-VQG-S---------------------PAD----------DG--RC-----GN--S-G--D-------------------TQSVTSFS--------------D-TVR-GYN---------KGV------RDLDANIHPYVVFGNVG--------SN-----------PGYTNFNP-QD-------HGI----------
EPLSLIAVVC----G-DQLV-----------YG-----------------------IWGDE-------------------------------NG-------------DDGPKA-----------VIGEASISLA
>ref_fungi_KPM38292.1Ruonan322/81-194
NMDIDCDG-VQG-S---------------------AAD----------DG--RC-----GS--S-G--D-------------------TQSVTSFQ--------------D-VVA-GYG---------TGQ------RDLDANVHPYVVFGNAG--------SK-----------PGYKTFDP-RA-------HGI----------
KPLSVMAVVC----G-SKLI-----------YG-----------------------VWGDE-------------------------------NG-------------DDGAQA-----------MVGEASISLA
>ref_fungi_KPM45293.1Ruonan302/81-194
NLDVDCDG-IQG-S---------------------PAD----------DG--RC-----GS--S-G--D-------------------TQSVTSFQ--------------D-IVE-GYK---------TGQ------KDLDANIHPYVVFGNTG--------TK-----------PRWPTFRP-QD-------YGI----------
KPLSVMAVVC----G-KKLI-----------YG-----------------------IWGDE-------------------------------NG-------------DDGEEA-----------MVGEVSISLA
>ref_GH75_BAA12799.1Ruonan304/81-194
NMDIDCDG-VQG-S---------------------PAD----------DG--RC-----GS--S-G--D-------------------TQSITSFQ--------------W-VLE-SYG---------TSQ------KDLDANIHPYIVFGNEG--------TK-----------KGWKTFDP-EK-------HGI----------
KPLSVMAVVC----G-NKMF-----------YG-----------------------IWGDE-------------------------------NG-------------DDGDQP-----------MVGEASISLA
>ref_fungi_ESU08894.1Ruonan307/99-206
NMDIDCDG-IQG-S---------------------SAD----------DG--RC-----GS--S-G--D-------------------TQSVTSFQ--------------D-RLR-TYS---------TKQ------KDLDANIHPYVVFGNLG--------TK-----------KNWPTFDA-QK-------HGI----------
KPLSIIAV-----------F-----------YG-----------------------IWGDE-------------------------------NG-------------DDGEES-----------MVGEAAISLA
>ref_fungi_KYK61821.1Ruonan400/165-278
DMDIDCDG-VQN-G---------------------PAD----------DH--RC-----RS--S-A--D-------------------IQAITAFA--------------E-TVR-SYG---------TGQ------PDLDPNAHAYVVLGNDG--------TK-----------PGWKTFDP-RN-------HGI----------
EPLSVVAVVC----K-DKLF-----------FA-----------------------VWGDT-------------------------------NG-------------DDGPQA-----------VVGEASIALA
>ref_fungi_EHK27511.1Ruonan460/80-192
NMDIDCDG-IQN-G---------------------PAD----------DG--RC-----GS--S-G--D-------------------TQSVTSFS--------------D-TVR-GYG---------TGQ------KDLDANAHPYVVFGNDG--------GR------------GWNVWDP-QS-------VGI----------
EPLSVMAVVC----N-NKLI-----------YG-----------------------VWGDT-------------------------------NG-------------DDGRYP-----------VVGEASISLA
>ref_fungi_PNY26806.1Ruonan419/83-196
NMDIDCDG-VQR-G---------------------PAD----------DG--RC-----GS--S-R--D-------------------TQSRTAFK--------------D-TVA-GYG---------SGQ------KDLDANAHPYVVFGNDG--------SK-----------PGWHRFNP-RQ-------YGI----------
EPLSVMAVVC----G-NKLI-----------YG-----------------------VWGDT-------------------------------NG-------------DDGREA-----------MVGEASIALA
>ref_fungi_PWI75067.1Ruonan782/525-638
NMDIDCDG-IQR-G---------------------PAD----------DG--RC-----GS--S-D--D-------------------TQSQTSFK--------------D-TVA-SYG---------TGQ------RDLDANAHPYVVFGNDG--------SK-----------PGWRKFNP-QQ-------YGI----------
EPLSVMAVVC----N-NKLI-----------YG-----------------------VWGDT-------------------------------NG-------------DDGPQA-----------MVGEASISLA
>ref_fungi_KAH0961975.1Ruonan368/82-195
NLDIDCDG-VQR-G---------------------PAD----------DG--RC-----GS--S-S--D-------------------TQSQSSFR--------------D-ELQ-GYG---------AGQ------KDLNAHVHPYVVFGNNG--------ER-----------PGWRKFDP-TK-------YGV----------
EPLSVMAVVC----D-NKLV-----------YG-----------------------IWGDT-------------------------------NG-------------DDAEEA-----------MVGEASISLA
>ref_fungi_KJZ79490.1Ruonan469/82-195
NMDIDCDG-AQG-G---------------------PAD----------DG--RC-----GK--S-T--D-------------------TQSQTSFR--------------D-ELA-GYG---------AGP------RDLDARIHSYVVFGNDG--------SK-----------RGWKTFDP-KR-------HGV----------
EPLSVMAVIC----K-DQLF-----------YG-----------------------VWGDT-------------------------------NG-------------DDGREA-----------MVGEASISLA
>ref_fungi_CRK42170.1Ruonan400/196-311
NLDIDCDG-IQG-G---------------------PAD----------DG--RC-----GS--S-G--D-------------------TQSQTSFQ--------------E-DLQ-SYG---------ANQ------TDLDANVHPXVVFGNESE------ENK-----------KGWSTFDP-SA-------HGI----------
EPLSIMAVVC----N-DQLY-----------YG-----------------------IWGDT-------------------------------NG-------------DDGDEA-----------MVGEASISLA
>ref_fungi_OLN85471.1Ruonan284/84-195
NMDVDCDG-AQ------------------------GEA----------DG--RC-----AS--S-T--D-------------------TQAETTFR--------------D-ELN-NYR---------TGQ------RDLNASVHPYVVFGNQG--------NK-----------PGWPTFNP-QD-------HGV----------
EPLSVMAVVC----N-NKLV-----------YG-----------------------VWGDT-------------------------------NG-------------DDGAES-----------MVGEASISLA
>ref_fungi_KAF6803952.1Ruonan285/83-196
NMDIDCDG-TQN-G---------------------PGS----------DG--RC-----QS--S-T--D-------------------TQSETTFR--------------D-DLR-GYG---------TGQ------RDLNAFVHPYVVFGNEG--------TK-----------SGWPTFDP-QK-------HGV----------
KPLSVMAVIC----N-NKLL-----------YG-----------------------IWGET-------------------------------NG-------------DDGAQA-----------MVGEASISLA
>ref_fungi_KDN70427.1Ruonan282/82-193
NMDVDCDG-AQ------------------------GGG----------DG--RC-----GS--S-T--D-------------------TQSQTTFR--------------D-VLR-GYS---------VGP------QDLNASLHPYVVFGNDG--------TK-----------SGWPTFDP-TQ-------HGV----------
KPLSVMAVIC----N-DKVV-----------YG-----------------------IWGDT-------------------------------NG-------------DDGPQA-----------MIGEASISLA
>ref_fungi_OHE92055.1Ruonan307/107-218
NMDVDCDG-EQ------------------------AGG----------DG--RC-----GS--S-T--D-------------------TQSETTFH--------------D-ELR-GYG---------TGR------RDLNASVHTYVVFGNEG--------TK-----------SGWPTFDP-AK-------HGV----------
KPLSVMAVIC----N-NKLL-----------YG-----------------------VWGDT-------------------------------NG-------------DDGPQA-----------MVGEASISLA
>ref_fungi_TEA15693.1Ruonan284/82-195
NMDIDCDG-IQN-G---------------------TGY----------DG--RC-----AS--S-T--D-------------------TQSQTTFW--------------E-TLR-GYN---------VGQ------SDLNSNVHPYVVFGNQG--------TK-----------QGWPTFDP-RA-------HDV----------
QPLSVMAVVC----N-KKLV-----------YG-----------------------IWGDT-------------------------------NG-------------DDGPEA-----------MVGEASLSLA
>ref_fungi_RCI08858.1Ruonan297/83-197
NMDIDCDG-DQQAT---------------------TPD----------DN--PC-----AS--S-T--D-------------------TQSQTSFS--------------D-TIK-SFD---------AGI------SDLNPHVHPYVVFGNDG--------TR-----------PDWPVYRP-QS-------HHV----------
QPLSVMAVVC----N-KKLI-----------YG-----------------------IWGDI-------------------------------NG-------------DDGQQP-----------MIGEASISLA
>ref_fungi_KOS16717.1Ruonan379/83-193
NMDIDCDG-QL------------------------SGG----------NG--DC-----GS--S-Q--D-------------------TQGQTSFM--------------D-TLK-GYK---------AGV------SDLNAYTHSYVVFGNEG--------SK-----------SGYVTFNP-QQ-------YGI----------
EPLSVMAVVC----G-NNMF-----------YG-----------------------VWGDT-------------------------------NG-------------DDGP-P-----------MVGEVSISIG
>ref_fungi_CEJ82058.1Ruonan512/82-192
NMDIDCDG-AL------------------------GTG----------DG--SC-----DS--S-T--D-------------------TQGNTTFR--------------D-TVQ-SYN---------KGI------DDLNAYVHSFVVLGNQG--------SK-----------DGYVEFDP-QS-------VGI----------
EPLSIVAVVC----A-DKMI-----------YG-----------------------VWGDT-------------------------------NG-------------DDGL-P-----------LIGEVSVSLG
>ref_fungi_EHK17399.1Ruonan359/81-191
NMDIDCDG-AL------------------------GTG----------DG--SC-----DS--S-E--D-------------------TQGETSFK--------------D-TVA-GYK---------KGI------KDLNAYVHSFVVLGNEG--------KK-----------SGYVTFDP-TS-------VQV----------
EPLSIVAVVC----G-NQMF-----------YG-----------------------VWGDT-------------------------------NG-------------DDGP-P-----------LVGEVSDALG
>ref_fungi_CEJ95169.1Ruonan255/82-192
NMDIDCDG-HQ------------------------SKG----------NG--DC-----AS--S-G--D-------------------TQSQTTWK--------------S-IVK-GYN---------KGI------DDVDAYIHSYVVLGNDG--------SK-----------SGYVTFDP-RK-------YGV----------
DPLSVVAVVC----G-DKMF-----------YG-----------------------VWADT-------------------------------NG-------------DDGP-P-----------LVGEASLSLG
>ref_fungi_KXJ95726.1Ruonan210/1-112
-MDIDCDG-ATR-S---------------------YDG----------DN--RC-----DG--S-T--D-------------------TQSQTSFK--------------D-ELKAKH-----------GI------DDLNAYVHSYVVFGNT--------GSR-----------PGYVNFDP-RD-------HGI----------
SPLSVMAVIC----G-DNLF-----------YG-----------------------VWGDE-------------------------------NG-------------DDGERA-----------AVGESSIALA
>ref_fungi_OAQ59425.1Ruonan251/80-191
DMDVDCDG-ATV-P---------------------IDG----------DT--RC-----NA--S-Q--D-------------------TIPETTFK--------------D-NVK-QF-----------GI------DDLNAYIHSYVVFGNT--------GSK-----------EGFVNFNP-QE-------FGM----------
EPLSVMAVVC----G-DQMF-----------YG-----------------------VWGDT-------------------------------NG-------------DDQPRA-----------AIGEASLSLA
>ref_fungi_ETN37840.1Ruonan258/83-193
DMDVDCDG-ALG-----------------------PGS----------DC--N-------S--G-G--D-------------------TQGQTSFK--------------N-IVS-TY-----------GI------SDLDASLHSFVVFGNDNS------GNL-----------PGFGTFRP-QD-------HGI----------
EPLSIMAVVC----N-GKLL-----------FG-----------------------VWGDE-------------------------------NG-------------NDGQHS-----------VIGEASLGLG
>ref_fungi_KIW55987.1Ruonan249/75-184
NMDIDCDG-AKN-----------------------PLC----------PV--R-------A--G-S--D-------------------TQLWTSFR--------------D-SVR-SY-----------GI------SDLDASIHNYVVLGNADD------DHT-----------PGYTTFRP-QD-------YGV----------
QPLSLMAVVC----N-SQLF-----------YG-----------------------IWGDE-------------------------------NG-------------SDGP-P-----------LVGEASISLA
>ref_fungi_KAG9252431.1Ruonan254/82-194
NMDIDCDG-IAN-G---------------------AGD----------DG--RC-----GN--S-E--D-------------------TQYQTAFQ--------------Y-TVS-SYS---------NGV------SDLNAYVHDYIVFGNYG--------SA-----------DGYTTFDP-QE-------YGV----------
KPLSIGAVVC----G-DKLV-----------YG-----------------------VWGDT-------------------------------NG-------------DDGN-P-----------LVGEASISLA
>ref_fungi_KFH42161.1Ruonan253/80-191
NMDIDCDG-LAD-G---------------------PGN----------DG--RC-----DS--S-D--D-------------------TQYQTAFK--------------H-IVS-SYS---------NGV------EDLNSYVHDYVVFGNYG--------GG-----------D-YINFHP-ED-------HNI----------
HPLSVMAVVC----G-DQLV-----------YG-----------------------VWGDS-------------------------------NG-------------DDGP-P-----------LIGEASLSLA
>ref_fungi_KAF2426282.1Ruonan267/84-200
NMDIDCDG-IQPPNR--------------------PGD----------DG--RC-----GS--S-T--D-------------------TQATTSFQ--------------D-VVQ-TYG-----------I------EDLNAFVHSYVVLGNYND-----AGGK----------PPKYVTFHP-EE-------HGV----------
EPLSVVAVVC----N-NQLL-----------YG-----------------------VWGDA-------------------------------NG-------------DDGP-P-----------LVGEASISLA
>ref_fungi_OAF55565.1Ruonan396/81-195
DMDIDCDGIIKS-----------------------PYS----------DG--RC-----EA--S-Q--D-------------------TQPQSSYI--------------E-QIA-RYN---------VGI------KDVDSYIHSYVVLGNYAD------QGK-----------PGYTTFHP-TD-------YGI----------
EPLSIVAVVC----N-NQLF-----------YG-----------------------VWADQ-------------------------------NG-------------DDGP-P-----------MVGEASLAIA
>ref_fungi_EXF74381.1Ruonan257/83-194
NMDIDCDG-AQ------------------------THG----------DG--RC-----GS--S-T--D-------------------TQSQTSFK--------------S-EIQ-GYK---------AGI------KDLNAYVHPYVVFGNDG--------SK-----------PGWPTFDP-KK-------HGI----------
EPLSIMAVVC----N-NKVI-----------YG-----------------------VWGDT-------------------------------NG-------------DDGNQA-----------MVGEASLSLA
>ref_fungi_KAH0436013.1Ruonan281/83-192
NMDIDCDG-AQ------------------------SSG----------DG--RC-----AS--S-T--D-------------------TQSQTSFK--------------S-RLS-SWG---------YG--------DLNSYIHPYVVFGNEG--------SK-----------PNWPNFDP-KE-------HGI----------
EPLSVMAIVC----N-NQLF-----------YG-----------------------IWGDT-------------------------------NG-------------DDGDQP-----------MVGEASIALA
>ref_fungi_KAA8647854.1Ruonan303/82-188
NMDIDCDG-KQ------------------------THG----------DG--RC-----GS--S-K--D-------------------TQGETAFK--------------D-ELK-KY-----------GI------SDLNAYVHPYVVLGNEG--------NY-----------HP--TFNP-RK-------HGI----------
EPLSVVAVVC----G-DKLV-----------YG-----------------------IWGDT-------------------------------NG-------------DDGK-P-----------LVGEASLATG
>ref_fungi_EAW08981.1Ruonan303/84-191
NMDIDCDG-EQ------------------------SGG----------DG--RC-----KS--S-T--D-------------------TQGQTTFS--------------T-NLH-KF-----------EI------GDLNANIHPYVVLGNEG--------NY-----------SP--TFDP-RS-------VGV-----------QPLSVVAVVC-
---R-DQLV-----------YG-----------------------IWGDT-------------------------------NG-------------DDGEHP-----------MVGEASIALA
>ref_fungi_GFF24824.1Ruonan872/644-752
NMDIDCDG-EQ------------------------SGG----------DG--RC-----KS--S-T--D-------------------TQGQTRWG-------------LR-HPE-KY-----------GV------GDLNANIHPYVVLGNEG--------SY-----------CP--TFDP-RT-------AGV----------
EPLGIVAVVC----G-DELV-----------YG-----------------------IWGDT-------------------------------NG-------------DDNEHP-----------MVGEASLALA
>ref_fungi_OXN07939.1Ruonan926/692-800
NMDIDCDG-EQ------------------------SRG----------DG--RC-----KS--S-T--D-------------------TQGQTRWG-------------LK-HLE-KH-----------GV------RDLNANLHPYVVLGNEG--------GY-----------SP--TFDP-RT-------VGV----------
EPLSIVAVVC----A-DELV-----------YG-----------------------VWGDT-------------------------------NG-------------DDNEHP-----------MVGEASLALA
>ref_fungi_EEA26294.1Ruonan1070/83-189
DMDIDCDG-NQH-----------------------GV-----------DS--RC-----HL--S-G--D-------------------TQPNTAFK--------------D-QLP-KYG-----------I------SDLRADIHPYVVLGNYG--------SY-----------S--PTFHP-ED-------HNI-----------KPLSVVAVVC-
---N-NKLI-----------YG-----------------------IWGDT-------------------------------NG-------------DDGP-P-----------LIGESSLAVG
>ref_fungi_EED22169.1Ruonan459/293-399
DMDIDCDG-SQK-----------------------GI-----------DH--RC-----KS--S-D--D-------------------TQDETAFK--------------D-QVS-KYG-----------I------SDLRADIHPYVVLGNYG--------DY-----------S--PTFDP-EQ-------HGI-----------KPLSLVAVVC-
---N-NKLI-----------YG-----------------------IWGDT-------------------------------NG-------------DDGP-P-----------LVGESSLAVG
>ref_fungi_OJJ80140.1Ruonan243/79-184
NTDIDCDG-DQQ-----------------------NA-----------DP--RC-----LG--S-S--D-------------------TQPATAFR--------------R-QVQ-R----------LSNI------TDLNASIHPYVVLGNIG--------A---------------VTFDP-RD-------HGI-----------EPLSVVAVVC----
G-GQLI-----------YG-----------------------IWGDI-------------------------------NG-------------NDGM-A-----------LVGEGSLALA
>ref_fungi_CEL02029.1Ruonan361/86-198
NMDVDCDG-IQN-ST--------------------GSG----------DG--RC-----TG--L-P--D-------------------TQAQTAFR--------------S-ILQ-A----------GYGI------PDLNTYIHPYVVLGNIG--------NR-----------PWYRTFDP-RT-------ADI----------
YPLSVVAVLC----G-NKLV-----------YG-----------------------VWGDV-------------------------------NG-------------DDGI-P-----------LVGEASLALA
>ref_fungi_PLN86131.1Ruonan320/87-194
NMDIDCDG-QEK-----------------------DSN----------D---RC-----GG--D-D--D-------------------TQYETRYK--------------D-EVK-K-----------MGI------PDLDTYIHPYVVLGNE---------RK-----------DGKATFLP-EK-------ANI----------
EPLSVVAVVC----N-DRLV-----------YG-----------------------VWGDT-------------------------------NG-------------DDGD-P-----------LIGEASLAMA
>ref_fungi_CAP59699.1Ruonan307/102-218
NMDVDCDG-VQG-G---------------------SQD----------DG--RCS---LGR--S-P--D-------------------YQDATAFR--------------D-LIQ-SFK---------VGI------SDLNTYVHPYVVFGNSG--------SK-----------PGWRTFDP-TD-------YGV----------
RPLSVMAVVCP---G-NKLV-----------YG-----------------------IWGDT-------------------------------NG-------------DDGDKP-----------MVGEVSIALA
>ref_fungi_KAF4471231.1Ruonan514/79-192
NMDVDCDG-AQG-G---------------------PQD----------DG--RC-----GE--S-T--T-------------------TIPTTAIK--------------S-IIE-GYN---------VGI------SDLNPHEHSFVVFGNSG--------TK-----------AGWKTFDP-RE-------YGV----------
QKASLMAVVC----G-DRVF-----------YG-----------------------IWSDS-------------------------------NG-------------DHGDRP-----------SVGEASLSLA
>ref_fungi_AEO69828.1Ruonan306/83-210
NMDVDCDG-ANG-GGGGGA---------------AVAAD----------DG--RCRP--STA--N-S--D-------------------TQGTTAFR--------------D-AVA-GYNR---------NI------TDLNPYVHPYVVFGNAA-------GTR------RR----RGWRAFDP-TA-------YGV-----
------RPLSAVAVVCP---G-YRLY-----------FG-----------------------VWGDT-------------------------------NG-------------DDGPKP-----------MVGEASLALA
>ref_fungi_AEO59530.1Ruonan497/272-403
NLDVDCDG-SSG-G---------------------PSD----------DG--RCR---REL--S-P--D-------------------LQNATSFR--------------D-VLA-SYGRA---------GV------AELNPYVHPYVVFGNAP-------ATKTTTASTGRGAKSGAWRAFDP-SE-------YGM-------
----RPLGVMAVVCPT--S-RKLV-----------YG-----------------------VWGDM-------------------------------NG-------------DDGARP-----------MVGEASLALV
>ref_fungi_EAQ93849.1Ruonan558/340-461
NLDVDCDG-TVG-G---------------------PSD----------DG--RCR---REL--S-P--D-------------------LQGATSFR--------------D-TLA-GYGHG--------NAT------ADLNPYVHPYVVFGNAV-------GTR------GR----AGWRAFDP-SA-------HGM----------
RPLSVMAVVCP---D-RRLV-----------YG-----------------------VWGDT-------------------------------NG-------------DDDEKP-----------MVGEGSLALA
>ref_fungi_EAA36355.3Ruonan305/85-212
NMDVDCDG-VQG-G---------------------ASD----------DG--RCS---VGV--S-A--D-------------------YQSTTAFQ--------------D-MVA-AYN---------VSGV------RDLNTYVHPYVVFGNEVEKNDNDNDNK-----------DLRWNVFDP-RA-------YLV----
-------EPLSVMAVVCDG--G-SKLV-----------YG-----------------------VWGDT-------------------------------NG-------------DDGDKS-----------MVGEASLALA

```
>ref_fungi_AEO66412.1Ruonan272/76-189
DMDVDCDG-AQR-A----------------------GSD-----------DG--RC-----NS----------------------------------------------------S-T--D--------------------TQGETAFR---------------S-TVA-GYG----------RGI------ADLDPFVHPYVVFGNEG--------SK------------PGWKTFDP-RA-------YGV-----------
EPLSVVAVVC----G-DQLL------------YA-------------------------IWGDT----------------------------------NG--------------DDGAKP------------MVGEASIALA
>ref_fungi_PHH68142.1Ruonan283/80-192
NMDIDCDG-DASTP----------------------FTN-----------DT--RC-----RS----------------------------------------------------S-L--D--------------------TQSQTTFR---------------D-QLL-PYG------------I------PDLNANIHTYVVFGNTA--------SK------------PGWPTFDP-AS-------HGI-----------
RPLSVMAVVC----G-QRLV------------YG-------------------------IWGDT----------------------------------NG--------------DDGENP------------MVGEASISLA
>ref_fungi_KAA8908972.1Ruonan266/96-203
NMDVDCDG-GIS------------------------GKN-----------DK--RC-----AS----------------------------------------------------S-K--D--------------------TQDITAFQ---------------D-QVS-RH------------GL------NDLDPTIIPYVVFGNTGN-------DKL---------------EFDP-TK-------HGI-----------
KPLSVMAVVC----N-NKLI------------YG-------------------------VWGDT----------------------------------NG-------------EED--EP------------KVGEASISLA
>ref_fungi_OTA97226.1Ruonan255/81-193
NMDTDCDG-DRS-K----------------------N-D-----------DG--RC-----DD----------------------------------------------------A-H--S--------------------VQSATAMR---------------D-YVS-AYK----------NGV------PDLNPFVHDYVVFGNSG--------DK------------DGWITFDP-RD-------HGM-----------
RPLSVMAVVC----N-NKLI------------YG-------------------------VWGDT----------------------------------NG--------------DDGVNP------------RIGEASISIG
>ref_fungi_OTB04574.1Ruonan257/81-194
NMDIDCDG-DRT-G----------------------FLD-----------DT--RC-----GS----------------------------------------------------H-P--T--------------------TQSMTAMK---------------R-YVS-SYS----------NGV------PDLNPFVHDYVVFGNTG--------ST------------PGWVTFDP-RR-------YGM-----------
EPLSLMAIVC----N-DKLI------------YG-------------------------VWGDT----------------------------------NG--------------DDGDNS------------RIGESSISIA
>ref_fungi_GAM88196.1Ruonan319/78-188
NMDIDCDG-DQK------------------------GNG-----------PG-TAC-----AS----------------------------------------------------S-S--D--------------------TQSETAFK---------------D-TVS-QYG------------I------RDLDANIHPYVVFGNQG--------SK------------AGYKNFDP-QA-------HGV-----------
RPLSVMAVVC----N-NKVV------------YG-------------------------IWGDT----------------------------------NG--------------DDGP-P------------LVGEAALSVG
>ref_bact_APR80523.1Ruonan244/95-190
DMDVDCDG--------KS----------------------------------T-AEC-----NP---------------------------------------------------S-T--DP-------------------AYQSETSAT-------------DSN-------------------GA------P-LDAAKLPYVVVP-------------------------LPSSKFDY-GA-------GGL-----------ALGSVIAVIY----K-
GKLA------------YG-------------------------VFGDE----------------------------------GP-------------KD-----------------IIGEASYAMA
>ref_bact_APR82716.1Ruonan283/134-229
DMDVDCDG--------KM----------------------------------S-AQC-----NI---------------------------------------------------D-V--DP-------------------AYQDQTSAT-------------DSN-------------------GD------P-LDAATLPFVVVP-------------------------LPSNKLDY-QA-------VGL-----------DLGSVIAVIY----
K-GKLA------------YG-------------------------VFGDE----------------------------------GP-------------KD-----------------IIGEASYAMA
>ref_bact_TRU57399.1Ruonan369/221-313
DMDIDCDG--------GS----------------------------------T-DTC----------------------------------------------------------K-A--DR-------------------TYQPETSC--------------QSS-------------------GK------P-LDASYLPYIVLP-------------------------KDSNGLAL-NN-------LGL-----------RCGSIAAVIY----N-
GKIV------------YG-------------------------VLGDR----------------------------------GP-------------AG-----------------VIGEASYAMA
>ref_bact_APR86904.1Ruonan419/272-365
DLDVDCDG--------GK----------------------------------G-AAC-----K--------------------------------------------------------A--DP-------------------YYQAETAAT-------------DSK-------------------GN------P-LDASTLPYVVIP-------------------------MASNGFDY-KQ-------AGL-----------KLGSVVAVIY----G-
GKIS------------YG-------------------------ILGDV----------------------------------GP-------------AG-----------------VIGEASYAMA
>ref_bact_TKC99219.1Ruonan231/84-177
DMDIDCDG--------GR----------------------------------G-AAC-----K--------------------------------------------------------A--DP-------------------YYQSETAAV-------------DAR-------------------GK------P-LDASTLPFVVVP-------------------------KPSHGFDF-KA-------QGL-----------KMGSVVAVLY----
K-GNVE------------YG-------------------------ILGDV----------------------------------GP-------------AG-----------------ILGEASYAMA
>ref_bact_SDN22910.1Ruonan373/89-184
DMDIDCDG--------VT----------------------------------T-SHC-----NS---------------------------------------------------S-T--DP-------------------WYQDQTSFQ-------------TST-------------------GN------W-FTSDVTRYFVIP-------------------------LPSSRFSY-QN-------AGI-----------TPGSVAAVVY----N-
GKVA------------YA-------------------------VFADE----------------------------------GP-------------ST-----------------IIGEGSYALA
>ref_bact_WP_168115188.1Ruonan225/78-173
DMDIDCDG--------VS----------------------------------T-SNC-----NS---------------------------------------------------R-T--DP-------------------WYQPQTAYR-------------TSK-------------------GG------Y-FQSDATHFFVIP-------------------------ASSSRFSY-SS-------AGI-----------RPGTVAAVIY----R-
GAVV------------YA-------------------------VFADV----------------------------------GP-------------SN-----------------VIGEASYATA
>ref_bact_TML25165.1Ruonan230/79-174
DMDVDCDG--------VK----------------------------------T-SHC-----NS---------------------------------------------------H-T--DC-------------------CYQGDTSFH-------------TSK-------------------GK------P-FQADLTHFYVIP-------------------------LPSSRFSY-TK-------AGI-----------KPGSIAALVY----N-
NKVL------------YA-------------------------VFADE----------------------------------GP-------------SN-----------------IIGEASYATN
>ref_bact_WP_030441780.1Ruonan219/73-168
DMDIDCDG--------VR----------------------------------T-SNC-----NE---------------------------------------------------N-T--DP-------------------WYQNQTSFE-------------TSA-------------------GK------S-FQADKTHYFVIP-------------------------LPSSRFSY-SS-------NGI-----------KPGSVAAIIY----H-
NKVV------------YA-------------------------VFADE----------------------------------GP-------------SN-----------------IIGEASYATA
>ref_bact_WP_168079847.1Ruonan218/73-168
GMSIDCDG--------VA----------------------------------T-PRC-----NA---------------------------------------------------G-T--DP-------------------AYQALTLYR-------------GAD-------------------GR------Y-LTADEARYFVIP-------------------------LPSSRFDY-RS-------AGI-----------APGNVGAIVY----D-
GKVV------------YG-------------------------IFADV----------------------------------GP-------------ET-----------------AIGEASYATA
>ref_bact_WP_203916775.1Ruonan563/106-201
DMAIDCDG--------EV----------------------------------T-DEC-----NP---------------------------------------------------D-T--DS-------------------TFSEGTNFK-------------TST-------------------GK------Y-FTADVTHYFVIP-------------------------LPSSRFDY-QN-------AGI-----------KPGNVGAVIY----N-
GKLV------------YA-------------------------VFADL----------------------------------GP-------------SD-----------------SIGEASYATA
>ref_bact_OLY91822.1Ruonan455/239-383
HAKICADG-A-------PD------AYKI--------GN-----------KG----TDDLRNA-------------GKPPSPNASTTKNLDRWSWWALVV--DEH------------N--NP---VIQTQSD-----SNPGYFVSTTAHVN------PQF-K-KTD-------------PR------------AYVNANEIPYIVLPKK--------------------------------------N--
--AGSA----------KLGDFAYVVDRK--T-GITC------------PA-------------------------IYSEV----------------------------------GP-------------KD-----------------ALGEISIKAA
>ref_bact_CCI25442.1Ruonan319/123-258
KMSIDADG-A-------PN------AYHP--------DN-----------IG----LDDLKNA-------------GY------------PNTSWWKNILVPD---------PQNP-N--RA---YEQPSG------PYQGYFVSMTALQD------GTK-A-KTD-------------PS------------RYVDSTRIPYIVLPGG---------------------------------G-------
SAGA----------KLGDFAVVFNGK--N-GKIV------------NG-------------------------IYADV----------------------------------GP------------SN------------------KIGEGSIALA
>ref_bact_TCT02366.1Ruonan230/29-167
YVSLDADG-T-------PR------AYHP--------DN-----------TG----LDDLANA-------------GY------------PHKG-WRGVLVTD---------PADP-A--RP---LVREGG------PYAGYFISKTSLHD------PDR-E-ETD-------------PL------------KYVDSETIPYIVLPGA---------FY---------------------AQ-------
AGTG----------YVGNLALARRSD--G-AMET------------AA-------------------------IIADI----------------------------------GP------------PKA-----------------PLGEMSLRLA
>ref_bact_VXC90397.1Ruonan226/30-168
HVTLDADG-S-------PR------AYHP--------QD-----------IG----IDANANA-------------GY------------PHRG-WRGALVVD---------PADS-D--RP---YVQPTG------DYAGYFVSKTSLRN------NSG-A-ATA-------------PS------------TYVNAETIPYIVFPGA---------FY---------------------GV-------
KGTG----------RYGDLAMVRSLA--N-GRVT------------AA-------------------------IVADG----------------------------------GP------------FKA-----------------PLGEMSIALA
>ref_bact_PHS66042.1Ruonan268/51-201
EVRVDADG-A-------PN------AYHP--------DDVSLHCTQGEGFKG----LDCPANG-------------GY------------PKHSWWRSAIVAG---------PDNP-A--QA---YVQPEG------TFKGFLVSRTSLQD------EQK-A-VTD-------------PQ------------RYVDSRSVPYLVFPGD---------FY---------------------
KK-------KGTG----------ALGDFGYALNLS--N-GQSS------------PF-------------------------IIADI----------------------------------GP------------ARA-----------------MLGEMSIALA
>ref_bact_AET95071.1Ruonan269/51-201
HAKVDADG-A-------PN------AYHP--------DDVGLNCIRDTGFRG----LDCPANG-------------GY------------PSQDWWRSAIVPD---------PHNK-N--VG---YIQPSG------EFKGFFVSQTSLKD------AQK-S-DLD-------------PT------------KYVNAATVPYLVFPGK---------FN---------------------
RL-------SGTG----------TLGDAGYAFNLD--N-GKVS------------PF-------------------------IVAEI----------------------------------GP------------SDA-----------------TLGEMSIALG
>ref_bact_AUT48628.1Ruonan269/51-201
HAQVDADG-A-------PN------AYHP--------DDVGLNCKTGTGFKG----LDCPANG-------------GY------------PNGDWWPSAIVPD---------PKNK-K--KG---YVQPDG------EFKGFFVSQTSLRD------GAR-S-DLD-------------PA------------KYVNAAAVPYLVFPGA---------FH---------------------
QK-------AGTG----------TLGDLGYAVNLD--N-GKTS------------AF-------------------------IVAEV----------------------------------GP------------SDA-----------------KLGEMSIALA
>ref_bact_PXM09777.1Ruonan270/57-206
KFSLDVDG-A-------PN------AYHP--------QDKGKNCKSSTDFLG----LDCLQNA-------------GY------------KNGN-WKSIIIPA---------PINS-D--EG---YVQPSG------EYKGYFVSQTSLKD------HSK-I-NTD-------------IN------------KYVNSLKIPYIVIPST---------LY---------------------SM-------
KGTG----------MLGDVGYAVNVT--N-GLES------------PF-------------------------IIAET----------------------------------GP------------KKS-----------------NLGEISFFLA
>ref_bact_WP_145339878.1Ruonan269/56-205
SFSLDVDG-A-------PN------AYHP--------QDKGKNCRTNKNFIG----VDCLQNA-------------GY------------KGGD-WKSIILPD---------PKKP-D--EG---YVQPDG------IYEGFFISQTSLKD------NKK-R-DTD-------------IS------------KYVNPIEIPYVAIPGE---------FY---------------------KR-------
KGTG----------KLGDVGYAINIK--T-GKSS------------PF-------------------------ILADI----------------------------------GP------------KNA-----------------SLGEISLFLA
>ref_bact_WP_027993824.1Ruonan270/54-202
RTAIDADG-A-------PK------AYHP--------DDVGKPCGATG--AG----LDCPSNA-------------GY------------PNKSWWPTVLASD---------PTNP-S--KA---FVQPSG------PAKGFFVSKTALQD------SANGN-ERD-------------TA------------RYVDASTIPYLVFPRP---------FF---------------------QL---
----VGTG----------KPGDLGMAFHLE--T-QKQT------------AF-------------------------IVADI----------------------------------GP------------DE-----------------PLGESSIALF
>ref_bact_QBM74625.1Ruonan283/54-202
HKLVDADG-A-------PN------AYHP--------ADVPNTCPETG--RG----IDCLAAA-------------GY------------PDGSWWQTVLVAD---------PADP-T--RP---AIQQDG------PYKGFFVSMTSLRN------TAFAG-PAS-------------TA------------SYVDAAAIPYIVLPAP---------IY---------------------RT---
----DGMG----------EMGDIGFATNLD--T-GQTT------------PF-------------------------VIADE----------------------------------GP------------VE-----------------RLGEASVAFW
>ref_bact_PIT05158.1Ruonan232/31-171
HAAVDADG-A-------PN------AYHP--------DD-----------IG----LDALANA-------------GY------------PHTNWWRDVLVPD---------PDHP-A--RA---YIQPSG------EYEGYFVAMTSLRA------PSG-A-RTD-------------PA------------TYVDATRFPYVVIPTG---------FE---------------------RL-------
PHVA----------RAGDVGFATHVP--T-GVTS------------TF-------------------------IVGDA----------------------------------GGG-----------SAA-----------------RLGEGSIALF
>ref_bact_KQN25083.1Ruonan266/52-201
HSRVDADG-A-------PD------AYHP--------DD---RMRSRPPYLG----LDNPQNA-------------GW------------PQTGWWPDVLTRD---------PANP-K--VP---YRQQSG------PYRGYFVSHTALRV------RGG-S-ETD-------------PA------------TFADSRTIPYIVLPGSD--------FP---------------------RR--
-----RGTG----------APGDVGWAWNLA--T-GKGT------------AF-------------------------VIGDT----------------------------------GGG-----------AGA-----------------RLGEASIAFF
>ref_bact_WP_168605515.1Ruonan269/53-205
HSRIDADG-A-------PN------AYHP--------AD---LQRKKAPYLG----LDNPANA-------------GWG---------LNSHASWWPDVLVAE---------PEHP-S--RP---YVQRSG------PFAGYFVSQTALRV------RGG-V-PTD-------------PK------------TYTDSNTIPYMVLPGSA--------FP---------------------
KL-------KGTG----------APGDVGIVWNLA--N-GKST------------PF-------------------------VIGDT----------------------------------GGG-----------ADA-----------------NLGEGSIALF
>ref_bact_EXJ36305.1Ruonan297/92-231
HKAVDADG-A-------PN------AYHP--------KN-----------KG----LDYLANA-------------GY------------PNKSWWRSVLVED---------PKNP-G--RA---YQQPDG------PFEGYFVSKTSLQD------SSK-E-ETD-------------SS------------RYVDATSIPYLVFPES---------FY---------------------LR-------
KGTG----------RLGDLGYAFNVA--T-GDAS------------PF-------------------------VIADI----------------------------------GP------------REA-----------------KLGEVSIALA
>ref_bact_GAC14840.1Ruonan259/52-191
HKAIDADG-A-------PN------AYHP--------EN-----------IG----LDNYLNA-------------GY------------PDSDWWPSVLVED---------PNHP-N--KP---YVQPDG------EFKGYFISKTSLFD------KQK-S-KLD-------------PS------------RYVDATHFPYLVYPGK---------FY---------------------SK-------
KGTG----------RLGDIGIAINLE--T-NDIQ------------SF-------------------------VVADV----------------------------------GP------------SNA-----------------SLGEMSMALA
>ref_bact_PME68446.1Ruonan249/47-185
YKAIDADG-A-------PN------AYHP--------DN-----------IG----LDHLANA-------------GY------------PNKG-WKNVLVVD---------PKQP-Q--KP---FVQTTG------DFKGYFLSKTTLED------KSK-P-PTD-------------SK------------RYVNSVEIPYIVFPGN---------FY---------------------SL-------
KGTG----------LKGDIGYAYNAK--T-KKSS------------AF-------------------------VFADI----------------------------------GP------------KSA-----------------PLGEMSIALA
>ref_bact_TNB47111.1Ruonan231/29-167
HMSIDADG-A-------PN------CYHP--------NDV----------SG----LDKLSYS-------------GY------------PDD--WSGVLVPD---------PADR-T--KP---YTQKRG------TYKGFFVSQTALCD------PAK-D-ETD-------------PA------------RYVDARHIPYWVFPPE---------FP---------------------PI-------
KGTG----------RLGDVGVAKNMT--T-GVTS------------SF-------------------------IIADV----------------------------------GP------------PNH-----------------PLAEVSIALA
>ref_bact_ASQ90483.1Ruonan282/63-202
HMTIDADG-A-------PD------AYHP--------DN-----------TG----LDHLKHA-------------GY------------PGTSWWKNVLVTD---------PKDP-E--KP---YVVPDG------PYQGYYLSMTSLRD------MTK-V-PTD-------------PS------------RYVDASRVPYLVFPST---------FS---------------------RL-------
KGVG----------ALGDLGVAVNLD--T-GVYS------------PF-------------------------IIADI----------------------------------GP------------EKH-----------------PLGEVSIQLA
>ref_bact_ABB23846.1Ruonan286/63-202
FMTIDADG-A-------PN------AYHP--------NN-----------SG----LDDLANA-------------GY------------PRHSWWNTVLVPD---------PADG-S--RP---YIMKSG------PYAGYYISMTTLKD------LRY-A-ETD-------------SR------------RYVDASRIPYIVFPET---------FA---------------------AM-------
DGVG----------QIGDYGVAVNLD--N-GSSS------------GF-------------------------VVADI----------------------------------GP------------RRH-----------------PLGEISIALA
>ref_bact_TCD48936.1Ruonan285/62-201
FMTIDADG-A-------PE------AYHP--------KN-----------TG----LDDLSNA-------------GY------------PRTSWWDTVLVPD---------PADN-S--RP---YILKSG------PHAGYYVSMTALKD------LRW-A-DTD-------------SR------------RYVDASRVPYIVFPET---------FA---------------------AL-------
EGVG----------QIGDYGVAVNLA--S-GCSS------------GF-------------------------VVADI----------------------------------GP------------RRH-----------------PLGEISIALA
>ref_bact_KAA6232620.1Ruonan275/70-209
FMTIDADG-A-------PD------AYHP--------RD-----------EG----LDMLKHS-------------GY------------PDKMWWKTVLVTD---------PADR-Q--RP---FVMPEG------PYAGYYLSMTALKN------MKK-A-KTD-------------PL------------RYVDASKVPYIVFPVT---------FA---------------------RK------
-RDVG----------ALGDFGVAVNLD--N-GMKS------------GF-------------------------VVADI----------------------------------GP------------ERH-----------------KLGEVSIALA
>ref_bact_PWB83328.1Ruonan269/56-198
KMSIDADG-A-------PD------AYHPK---NSPKSG-----------WG----RDYLANA-------------GF-------------GRNCNVIVC-------------KTE-K--HPEKGYFKTEEG-----PYAGYYVSASSLKD------SSV-EDRGD-------------YR------------RYVDAARVPYIAIPGSA---------A---------------------KK-----
--MGA----------QTGDLAYVVNMN--N-GERS------------PA-------------------------IFADI----------------------------------GT-------------EK-----------------TLGEGSIGLA
>ref_bact_RYX99046.1Ruonan644/443-569
KIAIDADG-S-------AR------AYHP-----DAGNP-----------DA----LDDIGNAN------------SY------------SKKYIQGQSR-NN-----------HV-G--K---------------GPRDGFYVSATSLQR------GD----DWD-------------AD------------AYVDAEFVPYIVLPDDFA--------P---------------------GV-------R-------
-------LGDLCTVVNLK--N-NRYC------------SA-------------------------IFADT----------------------------------NP---------------------------------DVGEASIRVA
>ref_bact_RAZ89077.1Ruonan242/48-172
KLAIDADG-S-------PT------AYHP--------GD-----------IG---SYDYLANVS--KNDLH-----GI----------------------------------QGED-G--AV-------------GPAPGFYVSGTSLSN------PAY-K-PSD-------------TR------------HWVDAATVPYFVINRQEFPQ---------------------------------------FNV-----------
PLGCVATVVDLQ--S-RKRT------------GA-------------------------ILADT-------------------------------------------------GR-----------------AVGEGSIALA
>ref_bact_WP_089837271.1Ruonan229/37-175
FLHVDVDG-A-------PN------AYGP---------------------PG-RKALDFEKNAH--EGAKLSGKVVGY--------------------------------LTEKHS-R--KP---VLQGPND-----PYPGYYISTTGFYD------RSV-DDETN-------------PR------------RYVNAAKINYVVLGDFGRR-------------------------------------------
HHV-----------KLGDFVAVYSSR--T-KKSV------------YA-------------------------IVGDE----------------------------------G--------------NP-----------------SGCEGSLALV
>ref_bact_QDI08867.1Ruonan252/55-192
RMSVDVDG-A-------PN------AYGP---------------------KN-KKTLDYELNAH--VGAKKSGAIVGY--------------------------------LVDMDG-N---P---IIQGKSD-----PAPGFYISKTGYVD------VKN-NNKFD-------------PR------------RYVNATEINYTLLADSAKK-------------------------------------------
KGV-----------KTGDFCVVHSVN--N-NKTV------------FA-------------------------IVGDT----------------------------------G--------------NS-----------------NGEEGSLALL
>ref_bact_AEU38989.1Ruonan246/39-178
RMDVDVDG-A-------PT------AYGP---------------------PS-KPTLDYLRNAH--LRGIPGAEIVGY--------------------------------LTDDDNPN--IP---VVQGPHD-----PAPGYYISQTAYTD------PAL-TNARD-------------LR------------RYVDATRINYVVLGREATR-------------------------------------------
KGA-----------RLGDFVAVHSQR--T-GHSV------------YG-------------------------IVGDD----------------------------------G--------------NP-----------------SGNEGSLHLL
>ref_bact_ADW70543.1Ruonan259/67-207
KMNVDVDG-A-------PN------AYGP---------------------PG-KKALDIDAHARAPKESEHPGAIVGY--------------------------------MTEYEG-G--PP---TKQGPHD-----PYPGYFVSQTDFAD------LSN-KRMED-------------PR------------RYVDAARINYLVQGRVAKQ-------------------------------------------
---TGV-----------ALGDFATVYSCR--T-GRTA------------FA-------------------------IVGDS----------------------------------G--------------NE-----------------SGAEGSLALV
>ref_bact_SNT33139.1Ruonan269/76-216
```

KMDVDVDG-A-------PN------AYGP--------------------PG-KKALDIEKHAHSPKDSEHPGAVVGY--------------------------------MTEYEG-G--PP---TVQGKND-----PYPGYYVSQTDFAD------KDN-KRMED------------PR-----------RYVDATKINYVVQGRVART--------------------------------
-----SGV-----------TMGDFATVYSCR--T-GKSV-----------YA------------------------IVADS--------------------------------G-------------NE----------------SGAEGSLALV
>ref_bact_PHM10734.1Ruonan298/99-234
GMSINADG-A-------PN------AYH-------PAD-----------KG----IDFLANA------------GY------------SDNWWALAV--D----------KN-G--NP---FIQSSTD-----PYPGYYISTTALFD------SGF-V-KQD------------PR-----------RYVDSTKIPYIVLPGNGD-------FR--------------------KA-------TGV--
---------KLGDFAVVYNTN--N-EKLA-----------FA------------------------IYADV--------------------------------GP------------KN----------------QIGEGSIALS
>ref_bact_BBM83306.1Ruonan536/343-474
GMSVDADG-A-------PK------AYH-------REKG----------KG----LDYLANA------------GY------------PGNWWGI-V--T----------KN-G--VP---VVQKSSD-----PAPGYYVSQTAMFS------SAK-S-IYD------------PR-----------RYVNASTIPYFVLPA----------R--------------------KS-------MGA-
----------KLGDIAAVVNTR--S-GKVA-----------YA------------------------MYSDV--------------------------------GP------------KN----------------HIGEGSIALA
>ref_bact_WP_085986336.1Ruonan308/117-244
GMSIDADG-S-------PR------AYHP--------KN----------TG----IDDLKHA------------GK------------GGKWWAIAT-------------KN-G--KP---VVQKS----------GYYVSMTSLQD------FRY-A-PWD------------QR-----------RYVNAETIPYIVLPPKV---------K--------------------QS-------GRV------
-----KLGDVAVVYNTR--N-KRWA-----------YA------------------------IYADT--------------------------------GA------------NT----------------RIGEGSIALA
>ref_bact_PKN36066.1Ruonan290/88-228
GMTIDADG-S-------PH------AYHP--------SN----------IG----IDD-NEN------------GK----------DEQGNWVGIVL--AN--------KK-------P---YIQNSTD-----PAPGYYVSATSLYD------MTK-R-RTD------------PR-----------RYVDSEKIPYIALPRQVMHPD--GRCQ--------------------RD-------
AKA-----------CLGDITIVRNIE--N-GRYA-----------FA------------------------ILADQ--------------------------------AP------------SS----------------GIGEGSIALA
>ref_bact_WP_079914689.1Ruonan242/46-178
IMDIDADG-S-------PR------AYNP--------QN----------TG----LDDLSHA------------GR------------PGHWVGIVT--HN--------GKPS-G--RP---VIQKRTD-----PAPGFYVSQTALSD------FNK-R-FTD------------PN-----------AYVNSEKIPFIVLPQ----------R--------------------RS-------FGA------
-----VLGDMAVVYNIK--N-GRLA-----------FA------------------------VYADT--DV----------------GIGEGSIALA
>ref_bact_TAE09863.1Ruonan283/84-220
KMSIDADG-S-------PR------AYHP--------QD----------KG----LDKLEYA------------GR------------QGNWWALVT--DN--------GQSN-G--NP---LIQQSGD-----PYPGYYISMTATVY------RGF-P-DNS------------PA-----------RYIDAEKIPYFVLNQEV---------M--------------------RS-------
TST-----------QKGDIGYVVYNRR--T-KKGC-----------YA------------------------IFGDV--------------------------------GG------------YG----------------KLGEGSIYLA
>ref_bact_TAE66675.1Ruonan321/61-198
GMTIDADG-S-------PR------AYHP--------DDS---------KA----LDDLKHA------------GS------------EGNWWALVT--DN--------GEKN-G--NP---IIQTEKD-----PAKGYYVSMTSLNN------PNY-P-ITS------------PL-----------RYADAEKIPYFVLNPLV---------M--------------------EQ-------
TGA-----------KVGDFGYVVYNEK--T-GKGS-----------FA------------------------IFGDV--------------------------------GN------------NQ----------------KLGEGSIYLA
>ref_bact_PYK28999.1Ruonan230/30-165
GMEIDADG-A-------YH------AYN-------PDGK----------SG----LDYLANA------------GH------------PGNWWALVT--DN--------GQPS-G--TP---LVQTDDD-----PAPGFYISTTSLED------ANC-D-PKD------------PR-----------RYVNAEAINFIVLPG----------R--------------------LG-------
LGA-----------KLGDFAVVIRSY--T-GAYD-----------YA------------------------VYADV--------------------------------GP------------AN----------------KIGEASIALA
>ref_bact_PXX41379.1Ruonan230/31-166
GMAIDADG-A-------AH------AYH-------PQTG----------MG----LDYLGNA------------GV------------HGNWWALVT--DN--------GMPS-G--KP---LLQKASD-----PAPGFYISKTSLED------KDR-A-LQD------------PR-----------RYVDAEVIPFFVLPG----------K--------------------FK-------
FGA-----------KLGDLGIAINTG--N-GKIC-----------GC------------------------IHADN--------------------------------GP------------SG----------------KIGEASIALA
>ref_bact_QLA12155.1Ruonan321/127-262
GMNIDADG-A-------FT------AYH-------PLPG----------KG----LDHLGNA------------GR------------PGNWWGIVT--HN--------TKRS-G--KP---VLQDATD-----PAPGYYVSGTSLQD------ATK-E-NND------------PT-----------RYVNSEAIPFFVLPG----------D--------------------SR-------IPA-
----------TMGDFGYVVNMD--T-AMSS-----------GC------------------------IFADV--------------------------------GP------------DN----------------KIGEGSIALA
>ref_bact_EKO39873.1Ruonan284/80-215
KMQINADG-A-------PK------AYN-------KDTS----------LG----LDNLANA------------GS------------AGNWWGIVT--DT--------GNAD-G--KP---ILQEKGD-----PAPGFYVSGTTLQD------STK-L-DED------------QK-----------RYVNSEAIPFYVLPK----------N--------------------IQ-------
IPM-----------KIGDFGFVVNRS--N-NAGS-----------GC------------------------IFADV--------------------------------GP------------ED----------------SIGEGSIALA
>ref_bact_WP_083634555.1Ruonan306/96-231
TMQINADG-A-------PT------AYH-------KNLD----------LG----LDYLANA------------GS------------KGNWWGLVT--DT--------GDSH-G--KP---ITQDAGD-----PAPGFYVSGTTLQD------STK-E-HGD------------QN-----------RYVDSETIPFYVLPK----------K--------------------IN-------
IPM-----------QIGDFGFVINRK--N-NTSS-----------GC------------------------IFADV--------------------------------GP------------ED----------------SIGEGSIALA
>ref_bact_RZK58430.1Ruonan237/47-179
GMAIDADG-A-------PK------AYH-------QNST----------LA----LDYLENA------------GK------------PGKWWALEV--D----------RK-G--QP---LLQKESD-----PAPGFYISKTSLFY------LNK-P-TTD------------PS-----------RYINSETVPFIAIPS----------G--------------------FA-------SDF--------
---QTGDLALVINKK--N-GKRS-----------YA------------------------IVADI--------------------------------GP------------RD----------------KIGEGSIFLA
>ref_bact_WP_138484733.1Ruonan237/47-182
GMMIDADG-A-------PK------AYH-------KDNR----------MA----LDFLANA------------GK------------PGNWWALVT--DT--------GKRD-G--QP---VTQSSGD-----PAPGYYISMTSLAN------GAK-K-TTD------------PS-----------RYVNSSGVPYIAIPP----------K--------------------FS-------
ASF-----------KLGDIALVVNKK--N-GKRC-----------YA------------------------IFADT--------------------------------GP------------AN----------------KIGEGSVFLA
>ref_bact_OYW53258.1Ruonan427/227-366
GMTICADG-A-------PR------AYH-------ADNS----------KA----QDYLANA------------GR------------PGAWYGICT--N---------KQT-G--EP---LEQGPDD-----PAPGYYVSTTALNN------AGA-K-AGT------------PA-----------LYVDAETIPYIVLPGGRYS-----GFC--------------------SA-------
RKL-----------RLGDVGLAYNRT--N-GHLS-----------YA------------------------QCCEV--------------------------------GP------------GR----------------ALGEGSIALA
>ref_bact_TBC68721.1Ruonan466/267-405
SFAIDADG-A-------PK------AYH-------KDSS----------IA----LDFLANA------------GR------------PGNWWALVV--N----------QN-G--EP---VVQGDGD-----PAPGFYISKTSLEN------PGF-E-AAD------------PR-----------RYVDAATIPYVVLPGVRYQ-----QFT--------------------QD-------
GNI-----------RLGDLGAAYNIA--T-GKLS-----------FT------------------------IFADT--------------------------------GP------------RT----------------KLGEGSIALA
>ref_bact_WP_024279069.1Ruonan426/227-365
KMAIDADG-A-------PK------AYH-------SNNA----------VA----LDYLANA------------GR------------PGNWWALVT--N----------AN-G--VP---RVQDARD-----PAQGYFVSKTALVY------DDT-D-PAR------------PE-----------HYVDAAKVPFFVLPGGRYK-----SFS--------------------KD----
---TPI-----------RLGDVGVAYNLK--T-KKMS-----------YA------------------------IFADI--------------------------------GP------------GD----------------KIGEGSIALA
>ref_bact_WP_194406846.1Ruonan426/227-365
RFAVDADG-A-------PK------AYH-------QNNA----------IA----LDFLANA------------GR------------PGNWWALVT--D----------AH-G--VP---RVQDNAD-----PAPGYYVATTSLTN------PGF-P-ADR------------PS-----------HYIDASTIPFVVLPGGRFR-----HFT--------------------SA-------
KVL-----------RLGDLGVAYNKL--N-GKLC-----------FC------------------------QFAET--------------------------------GP------------AD----------------KIGEGSIALA
>ref_bact_EDM79506.1Ruonan285/79-213
GMAIDADG-S-------PR------AYH-------PRSS----------VG----LDYLANA------------GR------------PGHWWALVV--D----------PD-G--AP---VVQGEGD-----PAPGYYVSMTALED------RGK-A-ETD------------PR-----------RYVDAERIPYVVLPAKA---------R--------------------TE-------
IGI-----------WLGDLALVVDPE--S-GEQA-----------FA------------------------LFADI--------------------------------GP------------NG----------------HLGEGSIALA
>ref_bact_PYP92796.1Ruonan244/49-182
GMTIDADG-A-------PN------AYGP--------KN----------KG----LDLTANAR-----------GA------------QG-WAALVT--S----------QK-G--AP---VRQKKG------PYRGFYISTTSLEQ------PDV-PDIAN------------PK-----------RYIDARTISYIVLPRNF---------A--------------------DR-------FGI-----
------SLGDLAIVFNET--T-GRSA-----------YA------------------------IFADI--------------------------------GP------------RG----------------RIGEGSIALA
>ref_bact_PYS23496.1Ruonan236/46-176
DIEIDADG-S-------PR------AYHP-----TNDK----------IA----LDFKAN-------------GF------------P---FAVVH-------------VN-G--KP---HIQGPND-----PAPGFFVSMTSLED------TSK-E-KTD------------PR-----------RYVDSVEIPYIVLPSGL---------I--------------------GN-------GKA----------
KLGDFAAVINKK--N-NKSS-----------FA------------------------IFADV--------------------------------GP------------KK----------------KLGEGSIALA
>ref_bact_RZB38342.1Ruonan264/71-205
GMAINADG-A-------PN------TYHP--------ED----------KG----MDFLANA------------GT------------PGNWRSLVT--HN--------GRTS-G--VP---VVQGPLD-----PFPGFYISTTALFD------PTK-P-ITE------------PG-----------CYVDSRRVPYFVLPM----------G--------------------RS-------GGA---
--------RLGDLGVVINLR--K-DLKS-----------SA------------------------IYADI--------------------------------GP------------CN----------------KIGEGSIRLA
>ref_bact_RFM26919.1Ruonan224/31-163
GMAIDADG-S-------PM------AYN-------ADSA----------KG----LDKLANA------------GS------------PGNWFGVMT--D----------AN-G--NP---IVQGPSD-----PAPGFYISQTSLQD------KTK-K-STD------------PR-----------RYADASSIPFIVLPS----------R--------------------KS-------FNA------
-----KLGDFCIVYNQK--T-RQVA-----------GG------------------------VYADI--------------------------------GP------------RT----------------KIGEASIAMA
>ref_bact_PYU86859.1Ruonan251/51-192
GVAVDFDG-A-------SR------CYGPH-----------------GIVG---ALDAFANAR------------------------HAGNWWGVVT--NT--------GHAA-G--QP---IVQSGVA--PAQPDRGFYISQTSLID------PMY-P-IDD------------VR-----------RYTDATKVPYVALPPAHMRG----------------------------------
--TGL-----------RIGDFCLAINMI--N-GRFS-----------YA------------------------VYADAKR--------------------------------QP--------------------------------NLGESSMRLV
>ref_bact_PZR11156.1Ruonan328/137-269
GMSVDADG-S-------PR------AYN-------PAN-----------TG----LDHLANA------------GK------------PGNWWGIAT--N----------SS-G--KP---YVQGAKD-----PAPGYYVSTTALTN------PKF-P-AND------------PR-----------RYVDSEKVPFIAIPPQ---------L--------------------RE-------QGV---
--------KLGDLVAVRNDK--T-GKTV-----------FA------------------------VVADI--------------------------------GP------------KD----------------HQGEGSIKLA
>ref_bact_WP_018970930.1Ruonan405/206-344
GLNVDADG-S-------PF------AYHP-----KPDSD----------KG----LDYLPNA------------GS------------PGNWWGIAT--EN--------GNDS-G--TP---VVQGPND-----PAPGYYVSTTSLED------PNY-P-ATS------------PQ-----------AYVNASEIPFIVLPLS----------H--------------------NN-------
FGG-----------AMGDLGAVLNLD--N-GKLA-----------YV------------------------IAADE--------------------------------GP------------PN----------------SLGEGSVALA
>ref_bact_APV40793.1Ruonan212/31-175
DADIDADG-A-------NGQYGAQAAYMV--------GD----------KG----SDYLENGG--MYEED-----GKV---------YCYA-PGKDNIAIVGD----------D-G--HP---RIYDGGIV----G------CKTSYKD------PTK-S-VSD------------LT-----------AYIDAATIPYIVVPVQVI--------K--------------------LT--
-----EGI-----------VLGCLARAT--Y--N-GQSV-----------DC------------------------VVADV--------------------------------SS------------SK----------------KVGELSIAAA
>ref_bact_KML46109.1Ruonan211/30-174
DADIDADG-A-------NGQNGEQAAYMV--------GD----------QG----SEFLANGG--MRMVD-----GKV---------ICAH-EWARSIVLLDT----------D-N--EP---KVFAGGVI----A------STTWYRN------RAR-L-ASD------------PA-----------SYVDAQTIPYVVVPPLIV--------T--------------------
SV-------IGV-----------VRGCRARVT--W--Q-GKSV-----------DC------------------------VVADL--------------------------------GP------------AN----------------RIGEISIAAA
>ref_bact_TDR36594.1Ruonan211/30-174
DADIDADG-A-------NGQGGGPAAYRT--------DD----------SG----TEALANGG--MKIVG-----GKV---------VCAR-AWARSIVILDA----------D-N--EP---KVFPGGVI----A------SMTWYRH------PGV-P-ASD------------PT-----------AYVDSETVPYIVVPPLIV--------Q--------------------KT--
-----AGV-----------VRGCRARVR--F--N-GKTV-----------DC------------------------VVADR--------------------------------GP------------AA----------------KIGELSIAAA
>ref_bact_TQK01128.1Ruonan394/213-357
DADIDADG-A-------NGQNGQAVAYRA--------DD----------TG----TEKLANGG--MRIDG-----GKV---------ICEK-AWARDVVILGA----------D-N--EP---KVFRDGVI----A------STTWYRH------PGK-A-PDD------------PS-----------AYVDAETVPYIVVPPLVV--------Q--------------------
KT-------VGI-----------VRGSKARVT--W--N-GKSV-----------DC------------------------VVADK--------------------------------GP------------SD----------------KIGELSIAAA
>ref_bact_ABS65322.1Ruonan200/26-159
GGAIDADG-ANG----QNG--AK-FAYRE-------D-D----------KG-L---DALQNA------------GY-----------PNGGWRNVLV-----------ERGD-G--EP------------LSDADGNIYSKTTYAW--------------------------------KGRPI--PKRYVDAATIPYVVVNPLVR-----------------------------RH------
AVGV-----------VIGCRALIT--Y--Q-QRSV-----------VA------------------------VVADVS--------------------------------GA--------------------------------TRIGELSIAAA
>ref_bact_TGD96588.1Ruonan200/26-160
GAAIDADG-ANG----QKN--VKHFAYRR-------D-D----------HG-L---DLLQNA------------GY-----------PDGDWRGVLL-----------DRGD-G--EP------------LDDGHGNLYSQTTYAW--------------------------------KGRPI--ATRYVDAKTVPYVVVNPIVR-----------------------------HH------
AVGV-----------VIGCHAQVS--F--Q-GKTI-----------EA------------------------VVADVS--------------------------------GA--------------------------------TRIGELSIRAA
>ref_bact_ROS27566.1Ruonan351/34-130
DADTDSDG-S-------PD-----------------------------------AEIIDPKF--G--TL---------------------QTSLSH------ENG---WKG------------KG-----------KFVNARIIPYFVLPNNWFKV------------------------------------TGIKIS---------LGDIAKLSYK-----
NASI------------YA------------------------IYADA--------------------------------GP------------KE----------------IIGEASISAV
>ref_bact_AEF46206.1Ruonan422/39-137
DADTDADG-S-------PD-----------------------------------AKEI-DAT---G--QI---------------------ETSLRKKR---KDGTG---WQG------------EG-----------DYVNARVIPYYVIPGNWRKV------------------------------------SGIDVK---------MGDLAKLSYN-
----DRSV------------YA------------------------ICADV--------------------------------GG------------EK----------------YIGEASIAAI
>ref_bact_WP_025160223.1Ruonan420/39-134
DADTDADG-S-------PD-----------------------------------AEDI-DPT---G--QK---------------------ETSLRR------GNG---WRG------------EG-----------DYVNARIIPYFVIPGNWKKI------------------------------------TGVAVN---------MGDMAKISYR-----
NNHI------------YA------------------------ICADV--------------------------------GG------------KD----------------SIGEASIAAV
>ref_bact_CRM05779.1Ruonan179/34-129
NASTDTDG-A-------PN-----------------------------------APAI-DPT---G--QL---------------------ETSLRR------SNG---WSG------------EG-----------EYVNSLVIPYFVLPGNWKEV------------------------------------TGIPCQ---------LGDIAKLTYK-----
AYSV------------YA------------------------IFADV--------------------------------GG------------VK----------------RIGEASVCAN
>ref_bact_PZQ37708.1Ruonan186/33-128
DAAVDADG-A-------PN-----------------------------------ADVI-DDY---G--QL---------------------ETSLRR------SRG---WRG------------EG-----------EFVNALTIPYFVLPGNWKAI------------------------------------TGIKCS---------LGDIAKLSLN-----
DAVV------------FA------------------------IYADV--------------------------------GG------------HD----------------SIGEASVKAI
>ref_bact_TRU31229.1Ruonan411/34-129
DADTDADG-S-------PD-----------------------------------ADQI-DDT---G--QL---------------------QTALGR------DNG---WKG------------DN-----------KYVNARIIPYYVLPGNWKEV------------------------------------TNVSCK---------LGDIAKVSYK---
--NKTV------------YA------------------------IYADV--------------------------------GP------------DE----------------IIGEASIATV
>ref_bact_CCI20451.1Ruonan412/34-129
DADTDADG-S-------PD-----------------------------------AVAIGNGS---G--QL---------------------QTSLGK------PR----WKG------------EG-----------DYVNARKIPYYVLPGNWQEV------------------------------------TGVNCK---------LGDIAKITYK----
-NKEI------------YA------------------------IYADN--------------------------------GP------------DE----------------IIGEASIAAV
>ref_bact_BAY09338.1Ruonan322/43-142
DMDADADG-S-------PR-----------------------------------ARIIDPKY---G--QL---------------------ATSLIK------PK----WLG------------ES-----------NYVNAETIPYFVLPANFNSV------------------------------------CGLPYEL-----RELLGDLALIRWQ----
-NREI------------FA------------------------IFADV--------------------------------GS------------RK----------------RIGEGSIKTI
>ref_bact_WP_024968724.1Ruonan304/43-139
DMDTDADG-S-------PR-----------------------------------ARTIDPKY---G--QL---------------------PTSLRK------SKG---WRG------------DA-----------EYVNAETIPYYVLPGNFASV------------------------------------SGVTCK---------LGDLALVRWQ---
--GQEI------------LA------------------------IYADQ--------------------------------GP------------SD----------------KIGEGSIKLV
>ref_bact_PIR18588.1Ruonan493/348-437
KMAIDADG--------AG--------------------------------G-AW---K-S--DA-----------------TGQSKTSLR------------YKN-----------------GD------S-LNPQELPFIVVP-----------------------LD---FHK-TH------PNV-----------GLGDYAAVTY----G-
VKTL------------YS------------------------IIGDK--------------------------------GP------------EG----------------VLGEGSMSLA
>ref_bact_SOD78690.1Ruonan258/98-190
KMGLDADG--------SP--------------------------------LS-KKT-----PG--L-T--D-------------------QPETSFRF-----------NVS-----------------GH------PSVDSDKVPFIVIPL----------------------GGFDT-----A------FGV-----------KTGDLAAVVF----N-
DKLV------------FA------------------------LIADK--------------------------------GP------------KC----------------KLGEGSIELH
>ref_bact_SHL04569.1Ruonan240/68-168
KMAIDTDGPSAG------------------------------------PG-RLN------------------GKA----------------------------------L--DPS-----------------SGQNDTSLH------------LAN-----------------GKG-------LPSEIIPYIVLPELQS--------------------GSSRTFNP---------AVAL------------GDVAIVIF----K-
DKTA------------AA------------------------VCGDL--------------------------------GP------------FN----------------KIGEASIRVH
>ref_bact_EDY21054.1Ruonan229/62-158
KLAIDADGVAAD------------------------------------PG-RRC------------------GTQ----------------------------------L--DPL-----------------DGQNDTSFH------------FPN-----------------GCP-------LSSERHPFIVLP--------------------------LGVFRA---------ATGL-----------SIGDLAVVIY----R-
DILT------------AA------------------------ICGDL--------------------------------GP------------SH----------------KIGEGSIRVH
>ref_bact_AWI30090.1Ruonan226/79-174
DLDIDCDG--------LG--------------------------------S-PEC-----NP--D-T--DE-----------------YWQPETAYW------------DSE-----------------GY------P-LDSADLPFIVVP-----------------------AISSVWDY-NY-------SRI-----------RGGTVAAVIY----R-
NRLV------------YA------------------------VVGDV--------------------------------GP------------TD----------------LIGEASHATA

>ref_bact_TQL78980.1Ruonan379/85-180
DFDVDCDG--------QR------------------------------S-SKC-----NE--N-T--DP------------------WYQPQTAFT------------QSD------------------GK------Y-LNSATLPHIVVP----------------------GVSSRWNY-KN-------SGI----------GGGTVAAVVY----
Q-DKVV------------YA------------------------VVGDV--------------------------------GP------------TA----------------GIGEGSYALA
>ref_bact_ADD41528.1Ruonan379/84-179
DFDIDCDG--------QR------------------------------T-DEC-----NE--N-T--DP------------------SWQNETAFP------------QSD------------------GQ------P-LNSATLPHIVVP----------------------LPSDKWDY-KG-------EGI----------EGGTVAAVTY----E-
DKVV------------YA------------------------VVGDL--------------------------------GP------------KP----------------IIGEGSYALA
>ref_bact_SHF41511.1Ruonan233/85-180
DMDIDCDG--------QA------------------------------T-AEC-----NG--R-T--DP------------------SFQPQTACV------------RSD------------------GR------P-LVASRLPYIVVP----------------------GVSSRWSY-KE-------SGI----------GCGTVGAVVH----K-
DKVV------------YG------------------------VIGDV--------------------------------GP------------KS----------------IIGEASYALA
>ref_bact_WP_019547414.1Ruonan250/100-195
DLDVDCDG--------QP------------------------------G-DEC-----NE--N-T--DP------------------AFQPETACV------------QSD------------------GE------P-LNSATLPHVVVP----------------------LPSDVWDH-AA-------SDV----------NCGTVGAVVY----
E-DKVV------------YG------------------------VIGDK--------------------------------GP------------DS----------------IIGEASYAMA
>ref_bact_WP_165237426.1Ruonan391/92-187
DLDVDCDG--------KR------------------------------T-AEC-----NE--N-T--DP------------------WWQNQTACV------------ESD------------------GQ------P-LNSATLPHIVIP----------------------LKSSTWDP-AA-------SGL----------GCGTVGAVIY----Q-
DKVV------------YG------------------------VIGDR--------------------------------GP------------SG----------------IIGEASYAAA
>ref_bact_RZQ59371.1Ruonan219/71-166
DFDIDCDG--------QV------------------------------T-AQC-----NK--N-T--DP------------------WFQPETHWQ------------QSD------------------GK------H-LNSAALPFIVVP----------------------SANSTWNF-NN-------HNI----------TGGTVAAVVY----
G-NRVA------------YA------------------------VVGDS--------------------------------GP------------VN----------------MIGEGSYKLA
>ref_bact_SDM91431.1Ruonan228/82-177
DFDIDCDG--------QV------------------------------T-TKC-----NK--Q-T--DP------------------YFQPETAWQ------------QSD------------------GK------H-LNSAALPFVVVP----------------------GISSTWSW-KA-------AGI----------TGGTVAAVVH----
K-DKVA------------YA------------------------VVGDI--------------------------------GP------------TK----------------IIGEGSYRLA
>ref_bact_WP_163509224.1Ruonan224/78-173
DLDVDCDG--------QR------------------------------S-TQC-----NK--Q-T--DP------------------DYQPDTAYH------------QSN------------------GK------P-LNAAKLPYIVLP----------------------APSSIWNF-TR-------SGI----------GGATVAAVVY----K-
NKVA------------YA------------------------VVGDT--------------------------------GP------------TG----------------IIGEGSYKLA
>ref_bact_WP_164600061.1Ruonan381/86-181
DFDVDCDG--------QR------------------------------T-TQC-----NE--N-T--DP------------------SFQPATSWT------------QSD------------------GR------P-LNSAGLPFIVVP----------------------LSSSIWNY-QT-------AGI----------AGATIAAVVY----Q-
DKVA------------YA------------------------VVGDS--------------------------------GP------------TG----------------IIGEGSYKLA
>ref_bact_WP_185001427.1Ruonan220/74-169
DLDIDCDG--------QR------------------------------T-AKC-----NP--S-T--DP------------------YWQNATAFP------------QSD------------------GK------P-LNAETLPFIVLP----------------------LASSTWNY-RN-------SGI----------TGGTVAAAVY----R-
DKVV------------YG------------------------VVGDL--------------------------------GP------------SA----------------IIGEASYRMA
>ref_bact_SDM91410.1Ruonan228/82-177
DFDVDCDG--------QK------------------------------T-PKC-----NK--S-T--DP------------------SWQNATAWP------------QSN------------------GK------P-LNAEKLPFVVVP----------------------GISSKWNY-KN-------SGI----------GGGTVAAVVY----
K-DKVA------------YA------------------------VVGDV--------------------------------GP------------KA----------------IIGEGSYALA
>ref_bact_AGM06707.1Ruonan377/77-172
GMTIDCDG--------QR------------------------------T-EKC-----NA--S-T--DP------------------SWQGQTAWA------------QPD------------------GK------P-LNAEKLPYIVVP----------------------GISSTWSY-AG-------SGI----------TGATVAAVVY----Q-
GKVA------------YA------------------------VVGDV--------------------------------GP------------TG----------------AIGEGSYALA
>ref_bact_SFA73062.1Ruonan378/77-172
GMTIDCDG--------QR------------------------------T-TNC-----NK--S-T--DP------------------YFQPQTSFP------------QSD------------------GK------P-LIADKLPFVVVP----------------------QATSTWNH-DQ-------SNV----------PGGTVVAVVY----K-
GKVV------------YA------------------------VVGDR--------------------------------GP------------KD----------------AIGEGSYALA
>ref_bact_TQJ04299.1Ruonan378/77-172
GMTIDCDG--------QR------------------------------T-AKC-----NS--S-T--DP------------------TYWHQTAWQ------------QPD------------------GK------Y-LNAEKLPYVVVP----------------------ISTSTWDH-YD-------SGI----------TGGTVVAVVY----
E-GRVV------------YG------------------------VVGDR--------------------------------GP------------RD----------------AIGEASYALA
>ref_bact_AHH99783.1Ruonan220/73-168
DMDIDCDG--------QP------------------------------S-THC-----SR--S-T--DP------------------DFQAETAFN------------DSH------------------GK------A-LRAETLPYVVVP----------------------GVSSIWNY-RD-------SGI----------RGGTVVAVIY----D-
NKLT------------YA------------------------VVGDV--------------------------------GP------------AK----------------AIGEASYATA
>ref_bact_WP_165234508.1Ruonan228/81-176
DMDIDCDG--------QP------------------------------T-ERC-----NK--K-T--DR------------------WFQPETAFQ------------QSD------------------GR------H-LNSAGLPFIVVP----------------------LPSKIWDY-RG-------AGI----------GGGTIAAVIH----K-
GEVQ------------YA------------------------VVGDL--------------------------------GP------------KK----------------IIGEASYASA
>ref_bact_WP_161694136.1Ruonan225/78-173
DLDIDCDG--------RP------------------------------S-QVC-----NK--R-T--DP------------------YFQPETAFQ------------DSR------------------GG------Q-LDSAALPYVVVP----------------------GASKMWNH-RD-------AKV----------WGGGLAAVIY----
R-GKVQ------------YA------------------------VVGDT--------------------------------GP------------TD----------------LIGEASYATA
>ref_bact_WP_187813211.1Ruonan293/147-242
DLDIDCDG--------RP------------------------------S-RVC-----NK--R-T--DP------------------FFLPTTAFQ------------DSA------------------GR------H-LDSATLPYVVVP----------------------GPSKVWDH-RR-------WGV----------HGGGVVAVIH----R-
GKVE------------YA------------------------VVGDT--------------------------------GP------------TD----------------LIGEASYATA
>ref_bact_SCK41613.1Ruonan228/81-176
DMDIDCDG--------RI------------------------------T-KAC-----NS--R-T--DP------------------SFQSQTAFP------------TSA------------------GK------P-LDSARLPYVVVP----------------------GPGPLWDY-RK-------SGI----------RGGGVAAVIH----D-
GKVR------------YA------------------------VVGDT--------------------------------GP------------TG----------------IIGEASYAAA
>ref_bact_PVC84861.1Ruonan259/112-207
DMDIDCDG--------RK------------------------------T-KAC-----NT--R-T--DP------------------YFQPQTAFQ------------TSR------------------GK------P-LDSARLPFVVVP----------------------GPGPIWDY-RK-------SGL----------TGGTVVAVIH----K-
GEVR------------YA------------------------VIGDT--------------------------------GP------------AG----------------IIGEGSYALA
>ref_bact_EHM26394.1Ruonan293/147-242
DMDIDCDG--------RR------------------------------T-KVC-----NR--K-T--DP------------------YFLPETAFQ------------SSR------------------GE------P-LDSAVLPHVVVP----------------------GPGKVWDY-RK-------SGL----------TGGSVVAVVY----
R-DRVR------------YG------------------------VIGDT--------------------------------GP------------TG----------------IIGEASYAMA
>ref_bact_SCK60819.1Ruonan317/171-266
DMDIDCDG--------RR------------------------------T-KVC-----NK--K-T--DP------------------YFQPQTAFT------------SSR------------------GK------P-LDSAALPFVVVP----------------------APSRTWDY-RK-------SGL----------TGGSVVAVIY----E-
GRVR------------YA------------------------VIGDT--------------------------------GP------------AG----------------IIGEGSYAMA
>ref_bact_TXS18782.1Ruonan225/78-173
DMDIDCDG--------QV------------------------------S-TVC-----NT--R-T--DP------------------SFQPQTAFQ------------GSD------------------GK------Y-LDSSEVPYVVVP----------------------GPGPLWNY-TT-------SGI----------KGGGVVAVIH----G-
DKVR------------YA------------------------VVGDT--------------------------------GP------------TG----------------IIGEGSYALA
>ref_bact_TRO60880.1Ruonan274/127-222
DMDIDCDG--------QV------------------------------T-AEC-----NK--R-T--DP------------------WFQDQTAFH------------QSD------------------GR------P-LNSEDLPYIVVP----------------------GPSGIWNY-AS-------SGI----------RGGSVAAVIH----G-
DEIQ------------YA------------------------VVGDT--------------------------------GP------------TK----------------IIGEASYATA
>ref_bact_PRY40270.1Ruonan238/91-186
DLDIDCDG--------KV------------------------------T-AKC-----NG--D-T--DP------------------WFQDDTAFH------------DSK------------------GA------P-LQADKLPYVVVP----------------------SPSGTWDY-RN-------SGI----------SGGGIVAVIY----N-
NKVE------------YA------------------------VVGDT--------------------------------GP------------TA----------------IIGEASYATA
>ref_bact_WP_185001842.1Ruonan221/75-170
DLDIDCDG--------QI------------------------------T-PEC-----NK--N-T--DP------------------WFQPDTAFH------------QSD------------------GK------P-LISARLPYVVVP----------------------SPSGTFDY-RK-------HGI----------VGGGVVAVIY----N-
NKVQ------------YA------------------------VVGDT--------------------------------GP------------AS----------------IIGEASYAAA
>ref_bact_POX46163.1Ruonan237/90-185
DMDIDCDG--------RP------------------------------T-AKC-----ST--S-T--DP------------------WFQDDTAFH------------QLD------------------GR------P-LRADRLPYVVVP----------------------GVSDIWDY-RA-------AGV----------KGGGLVAVIH----
D-DQVE------------YA------------------------VVGDV--------------------------------GP------------KK----------------IIGEASYATA
>ref_bact_AWT46396.1Ruonan244/97-192
DLDVDCDG--------QR------------------------------T-ERC-----NE--R-T--DP------------------WYQDDTAYH------------DSA------------------GR------P-LRADRLPYVVIP----------------------GPSGVWDH-RS-------AGV----------EGGSVVAVIR----
G-DRVH------------YA------------------------VVGDT--------------------------------GP------------KE----------------IIGEASHATA
>ref_bact_WP_108952784.1Ruonan248/102-197
DLDVDCDG--------LR------------------------------T-RLC-----NE--R-T--DP------------------YFRPDTAFH------------QAD------------------GM------P-LRADRLPYVVVP----------------------EPGRAWDH-AT-------AGV----------DAGDPVVLLH----
G-DRIA------------YA------------------------VVGDL--------------------------------GP------------AG----------------IIGEASYAAA
>ref_bact_ANS69194.1Ruonan235/88-183
DMDIDCDG--------QR------------------------------T-ARC-----NE--N-T--DP------------------WFQPDTAFH------------QSD------------------GR------P-LRSDTLPYVVVP----------------------SISETWNY-PG-------AGI----------GGGGVVAVIH----G-
GRVQ------------YA------------------------VVGDT--------------------------------GP------------KR----------------IIGEASYATA
>ref_bact_KOG42964.1Ruonan231/84-179
DMDIDCDG--------RR------------------------------T-AKC-----NE--R-T--DP------------------WFHPDTAFH------------QSD------------------GR------P-LRSDTLPYVVVP----------------------GASALWKP-AA-------SGI----------RGGGVVAVIH----D-
GKVR------------YA------------------------VVGDT--------------------------------GP------------SQ----------------IIGEASYAAA
>ref_bact_GCB44466.1Ruonan265/118-213
DMDIDCDG--------QR------------------------------T-VRC-----NE--D-T--DP------------------WFQGDTAFR------------QSD------------------GE------P-LKSDTLPYIVVP----------------------SPSRKWNY-RA-------AGI----------KGGGVAAVIH----R-
DQVQ------------YA------------------------VVGDT--------------------------------GP------------RK----------------IIGEASYATA
>ref_bact_SHI24002.1Ruonan275/128-223
DMDVDCDG--------QR------------------------------T-AKC-----NE--S-T--DP------------------WYQDDTAFH------------QSD------------------GK------A-LRADSLPYVVVP----------------------SSSSLWNY-GS-------AGI----------KGGGVVAVIY----
Q-NKVE------------YA------------------------VVGDT--------------------------------GP------------TK----------------IIGEASYATA
>ref_bact_WP_161180469.1Ruonan231/82-177
DMDVDCDG--------LR------------------------------T-DKC-----NK--D-R--DP------------------WYQDDTAFH------------QSD------------------GK------P-LRADALPYIVVP----------------------GSSALWDY-TK-------SGV----------KGGGVVAVVH--
--G-NKVE------------YA------------------------VVGDT--------------------------------GP------------SR----------------IIGEASYAAA
>ref_bact_WP_109279675.1Ruonan232/85-180
SMTIDCDG--------QR------------------------------T-AQC-----SE--A-T--DK------------------HFRNDTAVH------------RSD------------------HQ------P-FDAAVTPYVVLP----------------------WHSAIWNY-TT-------AGI----------RPGDVVAVIY----Q-
GQVE------------YA------------------------VFGDT--------------------------------GK------------EE----------------KIGEASYATA
>ref_bact_GDY31502.1Ruonan250/103-198
DMDIDCDG--------QR------------------------------T-SQC-----NE--N-T--DC------------------CFQADTSFH------------QSD------------------GR------P-LNAAKLPYVVIP----------------------LPSGRWDY-QK-------AGL----------QGGDVVAVIY----
N-GHVE------------YA------------------------VFGDE--------------------------------GP------------SG----------------IIGEASYATA
>ref_bact_QUQ62609.1Ruonan238/91-186
GMNIDCDG--------QT------------------------------T-AQC-----NK--N-T--DC------------------CYQNDTSFH------------LAN------------------GK------P-LSAAVTPYVVIP----------------------LPSSRFKY-GS-------NGI----------KGGDILAVVY----N-
NQVE------------YA------------------------VFGDE--------------------------------GP------------SD----------------IIGEASYATA
>ref_bact_QUQ66075.1Ruonan388/241-336
GMAVDCDG--------QR------------------------------T-ATC-----NE--N-T--DC------------------CFYNDTSFH------------QSD------------------GK------P-FDAAQVPYVVIP----------------------LPSSRWNY-GD-------AGV----------EGGDVLAVIY----
H-GHVE------------YA------------------------VFGDE--------------------------------GP------------DD----------------SIGEASYATA
>ref_bact_VVJ24105.1Ruonan233/86-181
DMDIDCDG--------QR------------------------------S-SQC-----NE--D-T--DC------------------CYQDDTAFH------------QSD------------------GK------P-LNAAKLPYIVVP----------------------SSSSIWKY-SS-------SQL----------KGGGSCAVIY----N-
GKVE------------YA------------------------VIGDT--------------------------------GP------------TQ----------------IIGEASYATA
>ref_bact_TDP98023.1Ruonan229/81-176
DMDIDCDG--------QR------------------------------T-TQC-----NE--D-T--DC------------------CFYPDTAFH------------TSD------------------DQ------P-LNAAELPYIVLP----------------------SPSGTWDY-GD-------YGL----------DGGSVVAIVY----N-
GHVE------------YA------------------------VVGDT--------------------------------GP------------TG----------------IIGEASYAAA
>ref_bact_TDP94022.1Ruonan362/86-181
DMDIDCDG--------VR------------------------------T-TQC-----NE--N-T--DC------------------CFYPDTAFH------------TST------------------DQ------P-LNAAQLPYIVLP----------------------QPTSNWDY-RN-------YGI----------DGGSVVAVIY----Q-
GRVE------------YA------------------------VVGDT--------------------------------GP------------TG----------------IIGEASYATA
>ref_bact_WP_084580438.1Ruonan356/78-173
DMDVDCDG--------VR------------------------------T-SQC-----NE--Q-T--DC------------------CFYPDTAFH------------TST------------------DQ------P-LNAAQLPYIVLP----------------------SPSGIWDY-RT-------AGI----------EGGAVVAVIY----N-
NQVT------------YA------------------------VVGDT--------------------------------GP------------TG----------------IIGEASYATA
>ref_bact_TDV54840.1Ruonan229/82-177
DMDIDCDG--------VR------------------------------T-TQC-----NE--D-T--DC------------------CFYPDTAFH------------TST------------------DQ------P-LNAAQLPFVVVP----------------------SPSSTWDY-RN-------YGI----------RGGGAVAIIY----G-
NKVE------------YA------------------------VVGDT--------------------------------GP------------TA----------------IIGEASYRAA
>ref_bact_WP_184833869.1Ruonan486/338-433
DMDIDCDG--------IR------------------------------T-AQC-----NE--D-T--DC------------------CFLPDTACH------------ASN------------------GS------A-LNAAQLPYVVVP----------------------SSSSIWNY-AN-------FQI----------GCGTVVAVIY----N-
NQVL------------YA------------------------VVGDT--------------------------------GP------------TQ----------------IIGEASYATA
>ref_bact_SNS42910.1Ruonan610/461-556
DMDIDCDG--------IR------------------------------T-TQC-----NE--D-T--DC------------------CFLPETACE------------TSG------------------GA------A-LNAAQLPYVVVP----------------------SSSGIWNY-TT-------KGI----------GCGTVVAVIY----N-
GQIE------------YA------------------------VVGDT--------------------------------GP------------SE----------------IIGEASYRTA
>ref_bact_WP_157247302.1Ruonan577/428-523
DMDIDCDG--------VR------------------------------T-AQC-----NE--D-T--DC------------------CFQPQTFCT------------ASG------------------GG------Y-LNAARLPYMVVP----------------------SPSGTWDY-RS-------RGI----------GCGTVVAIVY----
N-GQVE------------YT------------------------VMGDT--------------------------------GP------------AA----------------IIGEASYAAA
>ref_bact_ETK32074.1Ruonan349/200-295

DMDIDCDG--------VR----------------------------------T-TQC-----NE---N-T--DC--------------------CFQADTACH------------TSG--------------------DS------P-LNAAGLPYVVVP--------------------------STSSIWKY-TS-------VGI-----------GCGSVVAVIY----N-
GKIE-------------YA--------------------------VVGDT-------------------------------------GP-------------TQ------------------IIGEASYKTA
>ref_bact_TML28961.1Ruonan363/214-309
DMDIDCDG--------KT----------------------------------T-TQC-----NK---N-T--DC--------------------CYQNDTFCH------------DSH--------------------DA------P-LDSAHLPYIVVP--------------------------SESGIWNY-TK-------FGI-----------RCGQVVGVIY----N-
GKLT-------------YA--------------------------VMGDT-------------------------------------GP-------------TG------------------IIGEGSYNLA
>ref_bact_SDX78569.1Ruonan356/209-304
DMDIDCDG--------QR----------------------------------T-TQC-----NE---N-T--DC--------------------CFQPDTAFH------------QSD--------------------GK------P-LNAATLPYVVVP--------------------------SSSSTWDY-TK-------FGI-----------QGGGVVAVIY----N-
NQVL-------------YA--------------------------VVGDT-------------------------------------GP-------------SS------------------IIGEASYATA
>ref_bact_KAA2261911.1Ruonan233/86-181
DMDIDCDG--------QR----------------------------------T-AQC-----NE---K-T--DC--------------------CFQPDTAFP------------QSD--------------------GK------P-LNAAKLPYVVMP--------------------------SPSAIWDY-RK-------SGI-----------QGGSVVAVIY----N-
NKVT-------------YA--------------------------VVGDT-------------------------------------GP-------------NK------------------IIGEASYATA
>ref_bact_AXE82115.1Ruonan217/70-165
DMDVDCDG--------QV----------------------------------T-DLC-----NA---D-T--DP--------------------AFQNDTAFH------------QSN--------------------GD------P-LIADQLPYVVVP--------------------------GRSKIWNF-AR-------SGI-----------RGGGVVAVIY----
N-DTVQ-------------YA--------------------------VVGDI-------------------------------------GP-------------KN------------------IIGEASYATA
>ref_bact_CEL21509.1Ruonan224/77-172
DMDIDCDG--------QR----------------------------------T-ARC-----NE---Q-T--DP--------------------WFQPETAFE------------RSD--------------------GK------P-LIADQTHFVVLP--------------------------SESSTWRF-SS-------SGL-----------AGASACAVIY----N-
NKVL-------------YA--------------------------SVGDT-------------------------------------GP-------------QD------------------IIGEASYATA
>ref_bact_WP_037308310.1Ruonan228/82-177
DMDIDCDG--------QP----------------------------------T-ARC-----NK---N-T--DP--------------------WFQPDTAYP------------RSD--------------------GK------A-LIADQTPYIVVP--------------------------SISSTWNF-EK-------AGL-----------KGAGSCAVIY----N-
NKVL-------------YT--------------------------VIGDT-------------------------------------GP-------------KD------------------IIGEASYATA
>ref_bact_RZS36610.1Ruonan232/82-177
DMDIDCDG--------QR----------------------------------T-DKC-----NE---N-T--DP--------------------SFYPDTAFH------------QSD--------------------GK------P-LVADTLPFVVLP--------------------------GKSDIWDY-AA-------SGL-----------KGSGSCVIVY----G-
DKVL-------------YG--------------------------VVGDI-------------------------------------GP-------------KE------------------IIGEASYAAA
>ref_bact_GIL30293.1Ruonan229/82-177
DMDIDCDG--------QT----------------------------------T-DAC-----NS---D-T--DP--------------------WYQDDTAFH------------QSD--------------------GQ------P-LDAAGLPYVVIP--------------------------QNNDIFRY-SD-------HDI-----------DGGAVVAVIH----G-
DQVE-------------YA--------------------------VFGDT-------------------------------------GP-------------KN------------------IIGESSYATA
>ref_bact_OKJ75028.1Ruonan275/124-219
DLDIDCDG--------RA----------------------------------T-AAC-----NA---A-T--DP--------------------SFHGDTAFH------------ASD--------------------GR------P-LNAAELPYVVVP--------------------------APSGTWDY-AK-------SGI-----------RGGGVVAVVH----
G-GKVE-------------YA--------------------------VVGDV-------------------------------------GP-------------ER------------------IIGEASYATA
>ref_bact_WP_169133653.1Ruonan247/100-195
DMDIDCDG--------VE----------------------------------T-EQC-----NK---D-A--DP--------------------AYQDDTAFH------------TSD--------------------DQ------P-LNAEKMPYVVVP--------------------------SKSDIWNF-AD-------SGI-----------KGGGIVAVIS----G-
DKVT-------------YA--------------------------AVGDT-------------------------------------GP-------------EE------------------IIGEASYATA
>ref_bact_ROQ60187.1Ruonan279/132-227
DMDVDCDG--------QV----------------------------------T-TAC-----NE---D-T--DP--------------------WFQDDTAFH------------TSG--------------------DQ------P-LNAEKLPYVVVP--------------------------SSSSIWNY-GS-------AGI-----------KGGGVVAVIY----
N-GKVE-------------YA--------------------------VVGDT-------------------------------------GP-------------AK------------------IIGEASYATA
>ref_bact_THA79300.1Ruonan267/120-215
DMDVDCDG--------RV----------------------------------T-KEC-----NA---D-T--DP--------------------GFQGDTAFH------------TSD--------------------DR------P-LDARNLAYVVVP--------------------------SRSTLWDF-GS-------AGI-----------KGGGVVAVVH----
A-GRVE-------------YA--------------------------VVGDT-------------------------------------GP-------------AG------------------IIGEASYATA
>ref_bact_TXS39132.1Ruonan293/146-241
DLDIDCDG--------QV----------------------------------T-KAC-----NV---D-T--DP--------------------WFQGDTAFH------------TSG--------------------DR------P-LNAEELPYVVVP--------------------------SRSSLWDF-SD-------SGI-----------KGGGVVAVVH----
A-GKVE-------------YA--------------------------VVGDT-------------------------------------GP-------------AG------------------IIGEASYATA
>ref_bact_PJN02609.1Ruonan239/92-187
DLDVDCDG--------RA----------------------------------T-AAC-----NK---A-T--DP--------------------WFQPDTRFH------------QSN--------------------DK------P-LDAEKVSYVVVP--------------------------NASPLWNY-SA-------SGI-----------KGGGVAAVVY----
G-NRVE-------------YA--------------------------VVGDT-------------------------------------GP-------------KS------------------VIGEASYATA
>ref_bact_WP_030019196.1Ruonan239/92-187
DMDIDCDG--------QV----------------------------------T-AKC-----NK---N-T--DP--------------------WFQPETRWN------------QSD--------------------GK------P-LNAEKLPYVVAP--------------------------SVSGIWNF-ES-------AGI-----------KGGGIVAVIY----K-
NKVE-------------YA--------------------------VVGDS-------------------------------------GP-------------EK------------------IIGEASYAAA
>ref_bact_TFI20732.1Ruonan289/142-237
DLDIDCDG--------QV----------------------------------T-ARC-----NK---R-T--DP--------------------WFHGDTAFH------------QSN--------------------GE------P-LNAEKLPYIVVP--------------------------APSKIWDY-RA-------AGV-----------RGGGVAAVVY----
K-DRVE-------------YA--------------------------VVGDT-------------------------------------GP-------------AA------------------LVGEASYAAA
>ref_bact_KIF66552.1Ruonan252/105-200
DMDIDCDG--------QV----------------------------------T-AHC-----NS---A-T--DR--------------------WFQNSTAFS------------QSD--------------------GK------P-LNSEKLPYVVVP--------------------------APGGTWDY-TA-------SGI-----------NGGGIAAVIH----D-
GRVQ-------------YA--------------------------VVGDT-------------------------------------GP-------------AG------------------IIGEASYATA
>ref_bact_QIQ06330.1Ruonan253/102-197
DMDIDCDG--------QV----------------------------------T-AHC-----NT---T-T--DP--------------------WFQNQTAFS------------QSD--------------------GK------P-LNSEKLPFVVVP--------------------------APSERWTY-TA-------SGI-----------RGGGVVAVVH----
G-GRVQ-------------YA--------------------------VVGDT-------------------------------------GP-------------VG------------------IIGEASYAAA
>ref_bact_WP_078849624.1Ruonan172/24-119
DMDIDCDG--------KV----------------------------------T-RRC-----NE---D-T--DG--------------------SFQDMTAFT------------RSD--------------------GA------P-LDAAKLPYLVVP--------------------------DPGDTWDY-RS-------SGI-----------RGGGLAAVVY----
R-GRVE-------------YA--------------------------VVGDT-------------------------------------GP-------------AG------------------LIGEASYATA
>ref_bact_WP_107069048.1Ruonan202/55-150
DLDIDCDG--------QA----------------------------------T-ARC-----NP---T-T--DD--------------------SFQSMTAFS------------QSD--------------------GK------P-LNAEKLPYIVVP--------------------------GAGENWDY-RS-------SNI-----------KGGGVAAVIH----G-
DSVQ-------------YA--------------------------VVGDT-------------------------------------GP-------------AG------------------IIGEASYATA
>ref_bact_TQK51019.1Ruonan249/102-197
DMDIDCDG--------QR----------------------------------T-SAC-----NT---R-T--DP--------------------WFQSATAFQ------------QSD--------------------GL------P-LDAERLPFIVVP--------------------------TPSGIWDY-RT-------SDI-----------GGGTVAAVIY----G-
DRVQ-------------YA--------------------------VVGDT-------------------------------------GP-------------SG------------------IIGEASYATA
>ref_bact_SCF99708.1Ruonan272/125-220
DMDIDCDG--------QV----------------------------------T-DRC-----NT---R-T--DP--------------------YFQSMTAYT------------ESS--------------------GR------A-LNAKELPYIVVP--------------------------TPSAIWNY-RS-------SGI-----------SGGSVAAVIH----G-
DRVQ-------------YA--------------------------VVGDT-------------------------------------GP-------------PG------------------IIGEGSLATA
>ref_bact_WP_046496713.1Ruonan292/145-240
DMDIDCDG--------QV----------------------------------T-DRC-----NK---R-T--DP--------------------YFQSATSYT------------ESS--------------------GR------A-LNAKELPYIVVP--------------------------TPSRIWNY-RN-------SGI-----------SGGSVAAVIH----G-
DRVQ-------------YA--------------------------VVGDT-------------------------------------GP-------------PG------------------IIGEGSFAAA
>ref_bact_WP_129767759.1Ruonan309/162-257
DMDIDCDG--------QT----------------------------------T-AHC-----NS---A-A--DP--------------------YYQDSTAYL------------QSD--------------------GR------A-LNPEELPYIVVP--------------------------LSSSIWNF-RS-------SGI-----------RGGSVAAVIH----R-
DKVE-------------YA--------------------------VVGDT-------------------------------------GP-------------PG------------------IIGEASYATA
>ref_bact_ATW51487.1Ruonan249/92-187
DMDIDCDG--------RR----------------------------------T-ERC-----NR---D-T--DP--------------------SYLPTTSFT------------ESD--------------------GE------Y-LNAEKLPFVVVP--------------------------VPSRIWDY-RS-------SGI-----------RGGSVVAMIH----D-
GKVV-------------YG--------------------------VVGDT-------------------------------------GP-------------SG------------------IIGEASYAAA
>ref_bact_AXG76441.1Ruonan259/112-207
DMDIDCDG--------QI----------------------------------T-ERC-----NL---D-T--DP--------------------YFQNSTAYT------------ESD--------------------GR------T-LNAETLPYIVVP--------------------------MPSEIWNY-RA-------SGI-----------RGGSVAAVIH----A-
NKVV-------------YA--------------------------VVGDT-------------------------------------GP-------------PG------------------IIGEASYAAA
>ref_bact_KAA0930286.1Ruonan230/83-178
NLDVDCDG--------QV----------------------------------T-ARC-----NR---R-S--DP--------------------WFQAGTAFP------------QSD--------------------GR------P-LNSEKLPYVVVP--------------------------SPGRLWRY-AD-------SGV-----------RGGSVAAVMY----
E-GRLR-------------YA--------------------------VVGDT-------------------------------------GP-------------AD------------------LIGEASYATA
>ref_bact_WP_119295503.1Ruonan315/168-263
DMDIDCDG--------QV----------------------------------S-RQC-----NK---K-K--DP--------------------LFQAATAFQ------------QSD--------------------GK------P-LDSARLPYVVVP--------------------------GASKIWKH-ED-------SGI-----------KGGAVAAVIH----
D-GHLT-------------YA--------------------------VVGDT-------------------------------------GP-------------TD------------------IIGEASYATA
>ref_bact_WP_079185200.1Ruonan262/114-209
DMDIDCDG--------RP----------------------------------G-RYC-----NK---E-T--DP--------------------SFTAMTAFQ------------QSD--------------------GR------Y-LSAEELPFIVVP--------------------------ARGSRWKH-SD-------HGV-----------HGGSVVAVIH----E-
DKVL-------------YA--------------------------VVGDT-------------------------------------GP-------------TG------------------IIGEASYAAA
>ref_bact_OON72185.1Ruonan340/193-288
DLDVDCDG--------RP----------------------------------G-RHC-----DR---R-T--DP--------------------YFQNTTAYA------------QSD--------------------GR------S-LSADELPYIVVP--------------------------GAGSRWNP-SA-------SGV-----------RGGSVAAVIH----Q-
GRVV-------------YA--------------------------VVGDT-------------------------------------GP-------------AD------------------IIGEASWATA
>ref_bact_WP_106432938.1Ruonan299/152-247
DLDVDCDG--------RP----------------------------------G-RHC-----NR---R-T--DP--------------------LFLNATAHQ------------QSD--------------------GR------H-LSAEKLPYVVVP--------------------------GVSRIWNP-AR-------SGI-----------RGGTVAALVH----R-
GRVL-------------YA--------------------------VVGDT-------------------------------------GP-------------TD------------------LIGEASYAAA
>ref_bact_WP_161301096.1Ruonan269/119-214
DLDVDCDG--------RP----------------------------------G-RRC-----NA---E-T--DP--------------------MFADTTAFQ------------QSD--------------------GR------Q-LSAEQLPYIVVP--------------------------GSSDIWSP-AR-------SGI-----------RAGTVAALVH----R-
GRVV-------------YA--------------------------VVGDT-------------------------------------GP-------------TE------------------LIGEASYAAA
>ref_bact_WP_078490082.1Ruonan271/122-217
DLDIDCDG--------RP----------------------------------G-PVC-----NA---A-S--GP--------------------YFQGTTAWN------------GSD--------------------GR------P-LSADEVPYVVVP--------------------------GPSARWRP-AA-------SGV-----------TGGTLAVLVH----
G-GRVR-------------YA--------------------------VVGDT-------------------------------------GP-------------TD------------------VIGEASYAAA
>ref_bact_QES40097.1Ruonan260/113-208
DMDIDCDG--------RP----------------------------------G-KAC-----NR---R-T--DP--------------------LFQPATAFQ------------QSD--------------------GR------H-LDAENLPYVVVP--------------------------GPSRRWNH-TE-------HGV-----------TGGTVAAVVH----
R-DKVH-------------YA--------------------------VVGDT-------------------------------------GP-------------TD------------------LIGEASYATA
>ref_bact_TGB03022.1Ruonan250/103-198
DMDVDCDG--------RP----------------------------------G-KAC-----NR---R-T--DP--------------------LFLHGTAFQ------------QSD--------------------GR------Q-LDAERLPYVVVP--------------------------GASRRWNH-AA-------HGV-----------DGGSVAAVIH----
-R-GKVL-------------YA--------------------------VVGDT-------------------------------------GP-------------TD------------------LIGEASYAAA
>ref_bact_WP_098240884.1Ruonan327/180-275
DMDIDCDG--------RP----------------------------------T-KAC-----NR---R-T--DP--------------------LFFHATAFQ------------QSD--------------------GR------Q-LDAEHLPYVVVP--------------------------GASRRWKH-AA-------HGI-----------TGGTVAAVIH----
R-DKVQ-------------YA--------------------------VVGDT-------------------------------------GP-------------TD------------------LIGEASYATA
>ref_bact_TXS57519.1Ruonan237/83-178
DLDIDCDG--------VP----------------------------------T-THC-----NS---R-T--DR--------------------YFQPTTAFQ------------QSD--------------------GR------H-LNAEELPFVVVP--------------------------GVSGVWNH-RS-------SGV-----------RGGSVAALVY----R-
GRVV-------------YA--------------------------VVGDT-------------------------------------GP-------------AD------------------IIGEASYAAA
>ref_bact_ARP73549.1Ruonan251/86-181
DLDIDCDG--------RP----------------------------------T-VRC-----NR---R-T--DP--------------------LFTAATAFQ------------QSD--------------------GR------Q-LNAEALPYIVIP--------------------------APSRIWDH-RA-------AGV-----------RGGSVAAVVY----R-
GRLQ-------------YA--------------------------VVGDT-------------------------------------GP-------------RD------------------VIGEASYATA
>ref_bact_WP_079147025.1Ruonan240/93-188
DLDVDCDG--------RP----------------------------------T-ARC-----NP---R-T--DP--------------------SFTAATAFQ------------QPD--------------------DR------H-LSAEFVPYIVIP--------------------------APSGIWDH-RA-------HGI-----------GGGSVAAVIY----R-
GRVQ-------------FA--------------------------VVGDT-------------------------------------GP-------------RG------------------LIGEASYATA
>ref_bact_WP_106432297.1Ruonan246/98-193
DLDIDCDG--------RP----------------------------------T-AHC-----NH---R-T--DP--------------------TFAATTAFR------------QSD--------------------GR------M-LNAEKLPYIVVP--------------------------PAGPRWSH-RA-------HGI-----------KGGSVAAVLY----R-
DRVV-------------YA--------------------------VVGDT-------------------------------------GP-------------RD------------------VIGEASYATA
>ref_bact_SED85472.1Ruonan228/81-176
DLDIDCDG--------QR----------------------------------T-AHC-----NR---A-A--DP--------------------AFTAATAYQ------------QSN--------------------GR------Q-LNAEKLPYVVVP--------------------------GPSRRWNH-RK-------SGV-----------RGGAIAAVIY----
K-GRVQ-------------YA--------------------------VVGDT-------------------------------------GP-------------RD------------------IIGEASYATA
>ref_bact_WP_161333774.1Ruonan296/95-190
DLDIDCDG--------RP----------------------------------T-AHC-----NR---R-T--DP--------------------MFAATTAFQ------------QSD--------------------GR------H-LNAEKLPYIVIP--------------------------APSRRWNP-WR-------HGV-----------RGGAVAALVY----
R-DRVR-------------YA--------------------------VVGDT-------------------------------------GP-------------RD------------------LIGEASYATA
>ref_bact_WP_153542822.1Ruonan266/119-214
DLDIDCDG--------RS----------------------------------T-ARC-----NR---R-T--DP--------------------QFAGMTAYQ------------QSD--------------------GR------H-LNAETLPYIVVP--------------------------APSRIWNP-RK-------HGV-----------RGGSVAAVVY----R-
GRVQ-------------YA--------------------------VVGDT-------------------------------------GP-------------RD------------------LIGEASYATG
>ref_bact_WP_079071240.1Ruonan344/197-292
DLDIDCDG--------RP----------------------------------G-ARC-----NP---R-T--DP--------------------QFAATTAYP------------QSD--------------------GR------Y-LNAETLPYIVVP--------------------------APSPLWDP-GE-------LGV-----------RGGAVAAVVY----R-
GRVQ-------------YA--------------------------VVGDT-------------------------------------GP-------------SD------------------LIGEASYATA
>ref_bact_QIS69545.1Ruonan298/151-246
DLDIDCDG--------RP----------------------------------T-ARC-----NR---R-T--DP--------------------VFAPATAVV------------GSD--------------------GR------R-LNAETLPYIVVP--------------------------APSRRWDH-RA-------SGV-----------RGGSVAAVVY----G-
DRVR-------------YA--------------------------VVGDT-------------------------------------GP-------------RD------------------LIGEASYATA
>ref_bact_WP_171158894.1Ruonan269/122-217
DLDVDCDG--------RP----------------------------------T-THC-----NS---A-T--DP--------------------VFAPTTAFQ------------QSD--------------------GL------D-LNAETLPYIVVP--------------------------APSSRWDY-RA-------HGI-----------RGGSVAAVVH----G-
DRVQ-------------YA--------------------------VVGDT-------------------------------------GP-------------SD------------------LIGEASYATA

>ref_bact_WP_093892974.1Ruonan301/154-249
DLDIDCDG--------QA------------------------------T-VHC-----NG--Q-A--DP------------------LYSPSTAYV------------QSD------------------GQ------P-LNAETLPYIVVP------------------------AASSRWSP-GA-------SGV-----------RGGSVAAVVY----Q-
DRVQ------------YA------------------------VVGDT--------------------------------GP------------SD----------------LIGEASYATA
>ref_bact_WP_051866101.1Ruonan245/89-184
DLDIDCDG--------RP------------------------------G-AHC-----NR--R-T--DP------------------WFSRATAYQ------------QSD------------------GR------P-LSAETLPYIVVP------------------------APSSRWKP-RE-------HGV-----------RGGSVAAVVH----G-
DRVQ------------YA------------------------VVGDA--------------------------------GP------------AD----------------LIGEASYATA
>ref_bact_AWE48754.1Ruonan231/84-179
DMDIDCDG--------RP------------------------------T-ARC-----SR--R-T--DP------------------HFSSATAFT------------QSD------------------GR------Y-LSAEKLPYIVVP------------------------TPSTIWDY-RD-------HGI-----------RGGSLAAVIH----Q-
GRVR------------YA------------------------VVGDV--------------------------------GP------------YD----------------IIGEASYAAA
>ref_bact_SEB78192.1Ruonan330/183-278
DMDIDCDG--------QS------------------------------T-GHC-----NS--A-T--DP------------------YFSGSTAFQ------------QSD------------------GR------Y-LSAEHLPYIVVP------------------------TPSGIWDY-RA-------DSI-----------RGGSVAAVVY----G-
GRVQ------------YA------------------------VVGDV--------------------------------GP------------RD----------------IIGEASYATA
>ref_bact_KUO05092.1Ruonan255/88-183
DMDIDCDG--------RP------------------------------S-DHC-----NR--R-T--DP------------------YFSPVTAYQ------------QSD------------------GR------H-LNAERLPYIVVP------------------------TPSGIWDH-EA-------DGV-----------HGGAVAAVVY----
R-GRVR------------YA------------------------VVGDV--------------------------------GP------------RD----------------IIGEASYATA
>ref_bact_PJM96866.1Ruonan301/128-223
DMDIDCDG--------RP------------------------------T-ARC-----NR--S-T--DP------------------QFSPTAAYE------------QSN------------------GH------R-LDAARLPYVVLP------------------------APSRIWDH-RA-------HGV-----------GGGSVAAVVY----
G-DRVQ------------YA------------------------VVGDI--------------------------------GP------------RE----------------VIGEASYATA
>ref_bact_WP_112468756.1Ruonan286/92-187
DMDIDCDG--------RP------------------------------T-ARC-----NH--R-T--DP------------------HFSPGAAYT------------QSN------------------GH------R-LNAERLPYIVLP------------------------AASRIWDH-RA-------HGV-----------GGGSVAAVVY----
R-GRVQ------------YA------------------------VVGDI--------------------------------GP------------RG----------------IIGEASYATA
>ref_bact_WP_152772654.1Ruonan267/76-171
DMDIDCDG--------RP------------------------------G-PRC-----NR--A-T--DP------------------WFWPATAYK------------QSD------------------GR------Y-LNAETLPFIVLP------------------------APSDIWDH-RA-------HGV-----------RGGSVAAVIY----R-
DRVQ------------YA------------------------VVGDI--------------------------------GP------------RQ----------------IIGEASYATA
>ref_bact_WP_152861548.1Ruonan258/111-206
DIDIDCDG--------RP------------------------------G-SRC-----NH--T-T--DP------------------WFWPSTAYK------------QSD------------------GR------Y-LNAETLPFIVVP------------------------APSGIWDH-RA-------HGV-----------GAGSVAAVIH----E-
GRIQ------------YA------------------------VVGDI--------------------------------GP------------HH----------------LIGEASYATA
>ref_bact_PTM93123.1Ruonan231/84-179
DMDIDCDG--------QP------------------------------G-PRC-----NR--R-T--DP------------------LFSAATAYQ------------QSD------------------GR------A-LSAERLPYVVVP------------------------AADSVWNP-VA-------YGV-----------RGGTVAAVVY----
R-DRVQ------------YA------------------------VVGDI--------------------------------GP------------RG----------------LIGEASYATA
>ref_bact_WP_107104455.1Ruonan360/87-182
DMDIDCDG--------RP------------------------------G-PEC-----NS--R-T--DP------------------LFSGATAYQ------------QSD------------------GR------Y-LSAETLPYIVVP------------------------GVSDLWDH-RD-------HGV-----------RGGSVAAVVY----
R-DRVQ------------YA------------------------VVGDT--------------------------------GP------------SG----------------IIGEASYATA
>ref_bact_WP_079142171.1Ruonan331/87-182
DMDIDCDG--------SP------------------------------S-LRC-----NS--S-T--DP------------------LFSGATAYQ------------QSD------------------GR------Y-LSAETLPYIVVP------------------------GVSALWDY-RA-------HGV-----------GGGSVAAVVY----G-
DRVE------------YA------------------------VVGDI--------------------------------GP------------RE----------------IIGEASYATA
>ref_bact_AVV46401.1Ruonan330/87-182
DMDIDCDG--------RP------------------------------G-PHC-----NS--G-T--DP------------------LFSGTTAFQ------------QSD------------------GR------Y-LSADSLPYIVVP------------------------GASDVWDH-RD-------HGV-----------RGGSVAAVVY----
R-DRVA------------YA------------------------VVGDT--------------------------------GP------------RE----------------IIGEASYAAA
>ref_bact_SMF44955.1Ruonan316/87-182
DMDIDCDG--------RP------------------------------G-THC-----NS--T-T--DP------------------LFSDATAFT------------QSD------------------GR------Y-LSAETLPYIVVP------------------------GASDIWNY-RD-------HGV-----------RGGSVAAVVY----K-
DRVQ------------YA------------------------VVGDV--------------------------------GP------------SD----------------IIGEASYATA
>ref_bact_WP_107122262.1Ruonan316/87-182
DMDIDCDG--------RP------------------------------G-AHC-----NS--G-T--DP------------------LFSDTTAYT------------QSD------------------GR------Y-LSAETLPYIVVP------------------------QASDRWNP-RD-------HGV-----------RGGAVAAVVY----
R-DRVQ------------YA------------------------VVGDV--------------------------------GP------------PD----------------IIGEASYATA
>ref_bact_SHI73447.1Ruonan338/87-182
DMDIDCDG--------RP------------------------------G-TQC-----NS--G-T--DP------------------LFSDTTAYQ------------QSD------------------GR------Y-LSAETLPYIVVP------------------------GVSDIWNH-RD-------HGV-----------RGGSVAAVVY----R-
DRVQ------------YA------------------------VVGDT--------------------------------GP------------RE----------------IIGEASYATA
>ref_bact_PKT71840.1Ruonan236/89-184
DMDIDCDG--------RA------------------------------G-PLC-----NS--R-T--DP------------------LFSGTTAFQ------------QPD------------------GR------Y-LSAETLPYIVVP------------------------APSDIWNY-RD-------HGI-----------GPGSVAAVIY----Q-
DRVQ------------YA------------------------VVGDV--------------------------------GP------------QD----------------IIGEASYATA
>ref_bact_TQJ86073.1Ruonan291/144-239
DLDVDCDG--------RA------------------------------G-RRC-----NR--R-T--DP------------------QFSSLTAYQ------------QSD------------------GR------H-LSAERLPYIVVP------------------------AVSRLWNH-RD-------HGV-----------RGGSVAAVVY----
R-DRVQ------------YA------------------------VVGDI--------------------------------GP------------RD----------------VIGEASYATA
>ref_bact_WP_107122566.1Ruonan321/174-269
DMDIDCDG--------RP------------------------------S-RRC-----NR--R-T--DP------------------SFSAATAFQ------------QSD------------------GR------P-LDAGHLPYIVVP------------------------APSRISNY-RD-------HGV-----------RGGSVAAVVY----R-
DRVQ------------YA------------------------VVGDV--------------------------------GP------------RD----------------IIGEASYATA
>ref_bact_GAP49853.1Ruonan334/184-279
DMDIDCDG--------RP------------------------------G-PRC-----NR--R-T--DP------------------YFSDSTAYA------------QSD------------------GR------P-LSAERLPFIVVP------------------------APSRIWDY-RE-------HGV-----------GGGSVVAVVH----R-
NRVQ------------YA------------------------VVGDT--------------------------------GP------------QD----------------VIGEASYATA
>ref_bact_PWI06406.1Ruonan257/109-204
DMDIDCDG--------QP------------------------------G-VRC-----NT--R-T--DP------------------HFSATTAFT------------QSD------------------GR------P-LKSETLPFVVVP------------------------APSTLWDY-RA-------HGV-----------RGGSVVAVVY----R-
DRVQ------------YA------------------------VVGDT--------------------------------GP------------AG----------------IIGEASYATA
>ref_bact_WP_167990378.1Ruonan226/79-174
DMDIDCDG--------RP------------------------------G-TYC-----NA--R-T--DP------------------HFSASTAFA------------QSD------------------GR------P-LSSEQLPFIVVP------------------------APSTLWDY-RA-------SGV-----------RGGSVAAVIH----G-
DRVQ------------YA------------------------VVGDT--------------------------------GP------------AG----------------IIGEASYATA
>ref_bact_WP_121740414.1Ruonan340/192-287
DMDIDCDG--------RP------------------------------G-RHC-----NA--A-T--DP------------------HFSSSTAFA------------QSD------------------GR------A-LSSEKLPYVVVP------------------------APSSLWDY-RE-------HGV-----------RGGSVAAVVY----R-
DRVS------------YA------------------------VVGDT--------------------------------GP------------AD----------------IIGEASHAAA
>ref_bact_QER88154.1Ruonan383/235-330
DMDIDCDG--------RP------------------------------G-RRC-----NA--R-T--DP------------------SFSSSTAFA------------QSD------------------GR------A-LSSEKLPYVVVP------------------------APSDLWDY-RD-------DGV-----------RGGSVAAVVY----R-
DKVS------------YA------------------------VVGDT--------------------------------GP------------TD----------------IIGEASHAAA
>ref_bact_PNV35918.1Ruonan344/196-291
DMDIDCDG--------RP------------------------------S-RRC-----NA--R-T--DP------------------YFSSSTAFA------------QSD------------------GR------A-LSSEKLPYVVVP------------------------APSGIWDH-RD-------HGV-----------RGGSVAAVVY----R-
DRVS------------YA------------------------VVGDT--------------------------------GP------------TD----------------IIGEASYAAA
>ref_bact_WP_189558782.1Ruonan353/205-300
DMDIDCDG--------RP------------------------------G-RRC-----NA--R-T--DP------------------HFSASTAFA------------QSD------------------GR------P-LSAEKLPYVVVP------------------------APSAVWNH-RS-------DGV-----------RGGSVAALVY----
R-DRVR------------YA------------------------VVGDT--------------------------------GP------------AG----------------IIGEASHAAA
>ref_bact_GGP62105.1Ruonan274/126-221
DMDIDCDG--------RP------------------------------G-RRC-----NA--R-T--DP------------------YFSASTAFP------------QSD------------------GR------P-LSSEELPYVVVP------------------------APSTVWNY-RA-------HGV-----------RGGSVAAVIH----R-
DRVR------------YA------------------------VVGDV--------------------------------GP------------AD----------------IIGEASYAAA
>ref_bact_TQL18735.1Ruonan286/138-233
DMDIDCDG--------RP------------------------------G-PRC-----NS--G-T--DP------------------YFSASTAYA------------QSD------------------GR------P-LSSEETPYVVVP------------------------APSDVWDH-RS-------HGV-----------RGGSVVAVVY----R-
DRVR------------YA------------------------VVGDT--------------------------------GP------------AD----------------IIGEASHATA
>ref_bact_WP_078914010.1Ruonan307/159-254
DMDIDCDG--------RP------------------------------G-PRC-----NA--R-T--DP------------------YFSSSTAFV------------QSD------------------GR------P-LSSEETPYVVVP------------------------APSTVWNH-RS-------HGV-----------RGGSVVAVVY----R-
DRVR------------YG------------------------VVGDT--------------------------------GP------------AD----------------VIGEASYAMA
>ref_bact_PWJ08435.1Ruonan313/166-261
DMDIDCDG--------RP------------------------------G-RHC-----NV--L-N--DP------------------YFSAATAFA------------QSD------------------GR------P-LSSEDLPFVVVP------------------------APSAVWDY-RA-------HGV-----------RGGSVVAVVY----
Q-DRVQ------------YG------------------------VVGDT--------------------------------GP------------AD----------------VIGEASYAMA
>ref_bact_WP_155059586.1Ruonan319/172-267
DLDIDCDG--------RP------------------------------G-PHC-----NA--R-A--DP------------------HFSDTTAYQ------------QSD------------------GS------Q-LDAERLPYIVVP------------------------VPGSIWDH-RA-------HGV-----------AGGAVAAVVH----
G-DRVQ------------YA------------------------VVGDT--------------------------------GP------------HD----------------LIGEASYATA
>ref_bact_TKT09716.1Ruonan299/88-183
DLDIDCDG--------RP------------------------------G-RHC-----NA--R-T--DP------------------LFAPGTAFQ------------QSD------------------GR------D-LSAETLPYVVVP------------------------APSDIWDH-RR-------DGV-----------HGGSVAAVVY----G-
DRVQ------------YA------------------------VVGDV--------------------------------GP------------DD----------------IIGEASYATA
>ref_bact_KAA0942012.1Ruonan269/122-217
DMDIDCDG--------RP------------------------------G-RHC-----NA--R-T--DP------------------LFSENTAYQ------------QSD------------------GR------Q-LSAERLPYVVVP------------------------APSRRWNH-RK-------HDV-----------RRGTVAAVVH----
G-DRVG------------YA------------------------VVGDI--------------------------------GP------------DD----------------IIGEASYALA
>ref_bact_WP_190854307.1Ruonan306/159-254
DLDIDCDG--------RP------------------------------G-PHC-----NE--R-T--DP------------------LFSDTTAYQ------------QSD------------------GR------Q-LSAERLPYVVVP------------------------APSHRWNH-QE-------HDV-----------RRGTVAAVIH----R-
DRVR------------YA------------------------VVGDV--------------------------------GP------------DD----------------IIGEASYALA
>ref_bact_BAC68998.1Ruonan257/110-205
DMDIDCDG--------RP------------------------------G-LLC-----NE--S-T--DP------------------SFSDATAFQ------------QSD------------------GR------Q-LNAETLPYIVVP------------------------TAGDIWDH-RE-------HGV-----------RGGSVAAVIY----Q-
DRVQ------------YT------------------------VVGDT--------------------------------GP------------HD----------------IIGEASYATA
>ref_bact_WP_055612472.1Ruonan253/94-189
DLDIDCDG--------RR------------------------------G-PYC-----NE--S-T--DP------------------LFADATAFQ------------QSD------------------GN------Q-LDAATLPYVVVP------------------------AASHIWDH-EE-------HGV-----------RSGTVAAVIH----G-
DRVQ------------YA------------------------VVGDL--------------------------------GP------------EG----------------LIGEASYATA
>ref_bact_ELP70556.1Ruonan250/81-176
DMDIDCDG--------QA------------------------------G-PRC-----NA--R-T--DP------------------YFADTTAYQ------------QSD------------------GR------Y-LDPERLPFIVVP------------------------VASGIWNY-RD-------HGI-----------RGGSVAAVIY----K-
DRVQ------------YA------------------------VVGDT--------------------------------GP------------QD----------------IIGEASYAAA
>ref_bact_GAQ65826.1Ruonan314/153-248
DMDIDCDG--------RP------------------------------S-PHC-----NS--D-T--DP------------------WFQPTTSFL------------QSD------------------GR------Y-LRSESLPFVVVP------------------------VASRIWDH-RV-------HGI-----------RGGSVAAVVY----K-
DRVQ------------YA------------------------VVGDT--------------------------------GP------------QN----------------IIGEASYATA
>ref_bact_SBT89661.1Ruonan231/84-179
DLDIDCDG--------QP------------------------------G-HRC-----NS--E-T--DP------------------YFHDATAFQ------------QSD------------------GR------Y-LDAERLPYVVVP------------------------APSRVWDY-RD-------HGI-----------RGGSVVALVH----
E-DRVR------------YA------------------------VVGDV--------------------------------GP------------QD----------------IIGEASYAAA
>ref_bact_KOV53892.1Ruonan253/106-201
DMDIDCDG--------RP------------------------------G-DRC-----NS--S-T--DP------------------YFSAGTAFQ------------QSD------------------GR------H-LDAETLPYVVVP------------------------VPSAVWDH-RD-------HGV-----------RGGSVVAVVH----
G-DRVQ------------YA------------------------VVGDT--------------------------------GP------------HD----------------IIGEASYATA
>ref_bact_WP_104532217.1Ruonan340/193-288
DMDIDCDG--------QP------------------------------G-ARC-----NH--R-T--DR------------------SFTAATAYP------------RSD------------------GR------P-LDAERLPYIVVP------------------------GAGRLWDH-RA-------HGV-----------QGGSVAAVIH----
G-DRVR------------YA------------------------VVGDT--------------------------------GP------------RG----------------IIGEASYAAA
>ref_bact_WP_130335819.1Ruonan260/112-207
DMDIDCDG--------RP------------------------------G-TRC-----NR--R-T--DP------------------DFSPATAFP------------QSD------------------GR------P-LDAERLPYIVIP------------------------PAGPLWDH-RA-------HGV-----------RGGSVAAVLY----G-
DRVR------------YA------------------------VVGDT--------------------------------GP------------RD----------------IIGEASYATA
>ref_bact_WP_100086067.1Ruonan301/136-231
DMDIDCDG--------RP------------------------------G-RHC-----NR--R-T--DP------------------HFARSTAFQ------------GAD------------------GH------R-LDAEKVPYIVVP------------------------EPSRIWNH-RA-------DGI-----------RGGSVAAVIH----G-
DRLE------------YA------------------------VVGDT--------------------------------GP------------RD----------------IIGEASYAAA
>ref_bact_AIR96662.1Ruonan349/134-229
DMDIDCDG--------RP------------------------------G-RRC-----NR--R-T--DP------------------SFSAVTAFT------------GSD------------------GR------R-PDAERLPYVVVP------------------------APSRVWDH-RA-------HGV-----------RGGSVAAIVY----R-
DRVR------------YA------------------------VVGDV--------------------------------GP------------GH----------------IIGEASYAAA
>ref_bact_WP_053913342.1Ruonan283/136-231
DLDIDCDG--------RP------------------------------G-ARC-----NL--G-T--DP------------------HFSPATAYS------------ASG------------------GG------P-LDAERLPYIVVP------------------------EPSGIWDH-RE-------HGV-----------HGGSVAAVVH----G-
DRVR------------YA------------------------VVGDV--------------------------------GP------------RD----------------VIGEASYATA
>ref_bact_WP_161228481.1Ruonan352/205-300
DLDVDCDG--------RP------------------------------G-EHC-----NA--D-T--DP------------------HFTDATAYT------------GPD------------------GR------P-LDAERLPYVVVP------------------------EPSEIWDH-RE-------HGI-----------GGGSVAAVVY----E-
DRVG------------YA------------------------VVGDV--------------------------------GP------------RD----------------LIGEASYAAA
>ref_bact_CAC01568.1Ruonan388/241-336

DLDVDCDG--------RP------------------------------------G-DRC-----NS--G-T--DP-------------------HFSPATAYT-------------GSD-------------------GR------P-LDAERLPYVVVP-------------------------GPSDTWDH-RE-------DGV-----------RGGSLAALVH----
G-DRVR------------YA-------------------------VVGDV------------------------------------GP-------------SD------------------LIGEASYAAA
>ref_bact_CAJ89917.1Ruonan400/253-348
DLDVDCDG--------RP------------------------------------G-DRC-----NT--R-T--DP-------------------HFTPATAYT-------------RSD-------------------GT------P-LDAERLPYVVVP-------------------------QPSRIWDH-RA-------DGV-----------HGGSVAALVH----
G-DRVR------------YA-------------------------VVGDV------------------------------------GP-------------RD------------------IIGEASYAAA
>ref_bact_WP_107482063.1Ruonan295/148-243
DLDIDCDG--------RS------------------------------------G-DRC-----NA--R-T--DP-------------------HFTAATAYT-------------RSG-------------------GG------P-LDAEKLPYVVVP-------------------------RPSGIWNH-RA-------DGV-----------HGGSVVALVH----
G-DRVG------------YA-------------------------VVGDI------------------------------------GP-------------RD------------------VIGEASYAAA
>exp_virus_3300009181_____Ga0114969_10000130_90/20-159
GMMIDGDG-A-------RN-------IY-------AP--------------RGFGSPLDFIANA-------------GE-------------PGNWYGIVT--DN--------GKAS-G--KP---VIQKKGD-----PFPGYYVSATSYEF------PGF-S-RGD-------------PY------------RYVDSATVPYIVLPSHWR--------A--------------------EA-------
HGV------------VLGCKAQIEDTH--T-GNVI------------DS-------------------------VVADF------------------------------------GP-------------RG------------------KLGEASIAAA
>exp_virus_3300027754_____Ga0209596_1000127_18/20-159
GMMIDGDG-A-------RN-------IY-------AP--------------RGFGSPLDFIANA-------------GE-------------PGNWYGIVT--DN--------GKAS-G--KP---VIQKKGD-----PFPGYYVSATSYEF------PGF-S-RGD-------------PY------------RYVDSATVPYIVLPSHWR--------A--------------------EA-------
HGV------------VLGCKAQIEDTH--T-GNVI------------DS-------------------------VVADF------------------------------------GP-------------RG------------------KLGEASIAAA
>exp_virus_3300009159_____Ga0114978_10000954_18/18-159
GMAIDADG-ANGHDGSGRA------AY-------GP--------------KG-TNPLDYLANA-------------GK-------------EGNWWGIVT--------------KN-G--IP---VIQKRRD-----PAEGYYISTTSYQR------KPF-C-DTD-------------PD------------RYLDSATENFIVVPSHWR--------S--------------------EI-------
KGV------------VLGCKAEVKNTK--T-GKVV------------VG-------------------------IVGDF------------------------------------GP-------------KT------------------KLGEASMAIA
>exp_virus_3300027782_____Ga0209500_10002238_10/18-159
GMAIDADG-ANGHDGSGRA------AY-------GP--------------KG-TNPLDYLANA-------------GK-------------EGNWWGIVT--------------KN-G--IP---VIQKRRD-----PAEGYYISTTSYQR------KPF-C-DTD-------------PD------------RYLDSATENFIVVPSHWR--------S--------------------EI-------
KGV------------VLGCKAEVKNTK--T-GKVV------------VG-------------------------IVGDF------------------------------------GP-------------KT------------------KLGEASMAIA
>exp_virus_3300027754_____Ga0209596_1000555_48/23-167
GMRIDADG-ANGHDGSGRA------AYAD------GK--------------NG-TQPLDYLANA-------------GG-------------AGNWWGIVV--D-----------KQ-G--NP---IKQTADD-----PAPGYYISTTSYTH------PGK-L-ATD-------------PA------------RYLDAAVDRFIVLPRHWR--------A--------------------
EA-------RGV------------VLGCHCVVYDLR--T-TKSC------------DA-------------------------VVGDF------------------------------------GP-------------KD------------------KLGEGSIALA
>exp_virus_3300000730_____fpDRAFT_1008667_26/50-184
ARGLHVEG-QER-----VPL------VAYHP---------DD-----------EGPRRLWNHTEPK-------------GLD------------------------------------APENMT--GPK--VVQTSG-------PGAGAYLSETALVD------GRY-P-EGD-------------PR------------RYFDSSAVPGWVIPRGAF--
PGM----------DVALGDLAEITH----N-GVSV------------WA-------------------------MAYDV------------------------------------GP-------------ASS------------------KLLEMSVKTC
>exp_virus_3300032954_____Ga0335083_10000404_39/48-195
GAAIDADG-A-------PN------AYAL---------PS------------SG----LHGLDNIL--NALDDPGHVVYDS---------HGDP-IGPWAGVIVGP------------N-G--KP---IIQTSGPW----AG--FCLSPTALSD------HNF-P-ETD-------------YR------------RFVDATRIAYISVPPQLR--------Q--------------------M------
---GV------------CLGDGALVADRD--T-GLSI------------QA-------------------------LVADI------------------------------------GP-------------RG------------------HLGEVSVACA
>exp_virus_3300032893_____Ga0335069_10000845_57/21-152
GYEVDADG-C-------PR------AYHP---------GG-----------GG-----LDALGNAR-------------AD-------------DGAWVGVVT--N------------AM-G--SP---LVQGPDD-----PAPGYYISQTALQD------RSR-A-FDD-------------PA------------RYVDSSMVPYISIPPE----------L--------------------ER-------RGV-
-----------KLGDLVMVAYKE-----RAV------------AA-------------------------IVADV------------------------------------GP-------------HR------------------KLGEGSIALA
>exp_virus_3300027655_____Ga0209388_1000050_4/27-160
GYTINADG-S-------PH------AYAP---------DA-----------SG-LTALDYLANA-------------G-------------CPGNWWGIAC--D-----------SS-G--TP---YVQSPWH-----AAPGFYVSTTALCN------SKY-P-DHH-------------PS------------RYVDSERYCFAVLPG--------------------------------------G-------
TSYA-----------QLGDVGLAYNQA--T-GDNM------------YF-------------------------AVGDV------------------------------------GP-------------SD------------------QIGEGSMLLG
>exp_virus_3300021478_____Ga0210402_10000601_21/24-156
GATVNGDG-S-------PH------CYHP---------DD-----------GE---GLDYLANA-------------G-------------SPGNWWGIYT--D------------SS-D--RP---VVQSIYH-----PAPGYYISTTALVN------PAY-P-ENH-------------PD------------AYIDSERYPFCVVPGMF------------------------------------G-------QGW---
--------QLGDVGFCLNEK--T-DDNM------------YC-------------------------ATCDI------------------------------------GP-------------QN------------------HIGEVSMLLA
>exp_virus_3300026324_____Ga0209470_1001151_22/28-160
SFAVNADG-S-------PH------AYGP---------ND-----------SG-----LDYTDNA-------------G-------------KPGNWWGIYAPPD-----------GK-G--LP---ILQAAYH-----PAPYYYISTTALVD------PNY-P-ENH-------------PA------------RYVDSERYGFYVIPG------------------------------------G-------ESFC---
-------RLGDVGLALNTK--T-GDNM------------YF-------------------------AMADI------------------------------------GP-------------KD------------------QIGEGSMLLA
>exp_virus_3300021404_____Ga0210389_10001107_14/36-168
GAMVNADG-S-------PN------CYGP---------DN-----------SG----IDYTANG-------------G-------------TPGGNWWGGPT--D-----------SK-G--MP---VIQKIYE-----PSPGMYVGGTALIN------PTF-P-ESS-------------QY------------RYVNSEEIPFFVMPGTH-------------------------------------Y-------TGA----
-------KTGDCGLVLNLA--T-NDNC------------FA-------------------------VFGDV------------------------------------GP-------------KD------------------KIGEISIRLA
>exp_virus_3300020582_____Ga0210395_10003499_9/36-168
GAMVNADG-S-------PN------CYGP---------DN-----------SG----IDYTANG-------------G-------------TPGKNWWGGPT--D-----------SK-G--MP---IIQKIYE-----PSPGMYVGGTSLIN------PTF-K-ESS-------------QY------------RYMDSESIPFFVMPGTH-------------------------------------Y-------TGA-----
------KPGDCGLVLNLA--T-GDNC------------YG-------------------------IFADV------------------------------------GP-------------KD------------------KIGEISIRMA
>exp_virus_3300021403_____Ga0210397_10000139_99/44-176
GAMVNADG-S-------PN------CYGP---------DN-----------SG----IDYTANG-------------G-------------TPGKNWWGGPT--D-----------SK-G--MP---IIQKIYE-----PSPGMYVGGTSLIN------PTF-K-ESS-------------QY------------RYMDSESIPFFVMPGTH-------------------------------------Y-------TGA-----
------KPGDCGLVLNLA--T-GDNC------------YG-------------------------IFADV------------------------------------GP-------------KD------------------KIGEISIRMA
>exp_virus_3300021479_____Ga0210410_10001803_30/44-176
GAMVNADG-S-------PN------CYGP---------DN-----------SG----IDYTANG-------------G-------------TPGKNWWGGPT--D-----------SK-G--MP---IIQKIYE-----PSPGMYVGGTSLIN------PTF-K-ESS-------------QY------------RYMDSESIPFFVMPGTH-------------------------------------Y-------TGA-----
------KPGDCGLVLNLA--T-GDNC------------YG-------------------------IFADV------------------------------------GP-------------KD------------------KIGEISIRMA
>exp_virus_3300021405_____Ga0210387_10000245_25/44-176
GAMVNADG-S-------PN------CYGP---------DN-----------SG----IDYTANG-------------G-------------TPGKNWWGGPT--D-----------SK-G--MP---VIQKIYE-----PSPGMYVGGTSLIN------PTF-K-ESS-------------QY------------RYMDSESIPFFVMPGTH-------------------------------------Y-------TGA----
-------KPGDCGLVLNLA--T-GDNC------------YG-------------------------IFADV------------------------------------GP-------------KD------------------KIGEISIRMA
>exp_virus_3300021405_____Ga0210387_10000062_108/36-168
GAMVNADG-S-------PS------CYGP---------NN-----------TG----IDYTANG-------------G-------------TPGGNWWGGPT--D-----------SK-G--MP---VIQKIYE-----PKPGMYVGGTALIN------PTF-P-ESS-------------QY------------RYVNSEEIPFFVMPGTH-------------------------------------Y-------TGA----
-------KTGDCGLCLNLA--T-GDNC------------YG-------------------------IFADV------------------------------------GP-------------KD------------------KIGEISIRMA
>exp_virus_3300026285_____Ga0209438_1001046_4/31-164
GMAIDCDG-S-------PN------AYGP---------NN-----------SG-----LDYTANG-------------GD-----------DQGGNWWGGPT--G-----------SN-G--KP---LVQKIYE-----PSPGMYVSGTAHLD------PAY-T-DDS-------------QY------------RYTDSGSIPFFVLPVNH-------------------------------------A-------
NGA-----------KLGDCGLAYNEQ--T-GDNC------------YA-------------------------IYADV------------------------------------GP-------------SS------------------KIGEASMRLA
>exp_virus_3300026285_____Ga0209438_1000629_3/31-164
GMTIDADG-A-------PN------CYGP---------NN-----------SG----IDYTANG-------------GD-----------DSGGDWWGGPT--D-----------SN-G--YP---IKQKIYD-----PYPGYYVSGTAHTN------PQY-D-SES-------------PY------------CYLDSSCIPFFVLPGQH-------------------------------------A-------NGA---
--------RLGDVGLVYNEK--T-GDNC------------YA-------------------------IYGDI------------------------------------GP-------------SS------------------KIGEGSMRLA
>exp_virus_3300026304_____Ga0209240_1000230_3/31-164
GMTIDADG-A-------PN------CYGP---------NN-----------SG----IDYTANG-------------GD-----------DSGGDWWGGPT--D-----------SN-G--YP---IKQKIYD-----PYPGYYVSGTAHTN------PQY-D-SES-------------PY------------CYLDSSCIPFFVLPGQH-------------------------------------A-------NGA---
--------RLGDVGLVYNEK--T-GDNC------------YA-------------------------IYGDI------------------------------------GP-------------SS------------------KIGEGSMRLA
>exp_virus_3300026551_____Ga0209648_10005312_14/31-164
GMTIDADG-A-------PN------CYGP---------NN-----------SG----IDYTANG-------------GD-----------DSGGDWWGGPT--D-----------SN-G--YP---IKQKIYD-----PYPGYYVSGTAHTN------PQY-D-SES-------------PY------------CYLDSSCIPFFVLPGQH-------------------------------------A-------NGA---
--------RLGDVGLVYNEK--T-GDNC------------YA-------------------------IYGDI------------------------------------GP-------------SS------------------KIGEGSMRLA
>exp_virus_3300033158_____Ga0335077_10000474_60/29-164
PLAVDADG-N-------PQ------AYHP------DGSP-----------PG----LDYLANA-------------GS-------------PGNWWGIAC--D-----------SK-G--NP---YVQSQEN-----VAPGFYVSTTALVA------PGF-H-DSH-------------PA------------KYVDSGLIPFIVLPSKP---------T--------------------FS-------PEQ---
--------TLGDLAMVFNNK--T-GKCS------------WA-------------------------LYADI------------------------------------GP-------------AN------------------QLGEGSMALC
>exp_virus_3300021560_____Ga0126371_10000592_23/29-164
PLAVDADG-H-------PE------AYHP------GGSP-----------PG----LDYLGNA-------------GS-------------PGNWWGIAC--D-----------AK-G--KP---YIQGGDH-----PAPGFYVSTTALQD------NTI-Q-SHN-------------PA------------RYVDSGRIPFIVLPSSP---------K--------------------FS-------DKQ---
--------TLGDLAMCFNTQ--T-GNLS------------WA-------------------------IYADI------------------------------------GP-------------AN------------------AIGEGSMELC
>exp_virus_3300021560_____Ga0126371_10004477_11/31-166
KLNVDADG-H-------PQ------CYHP------AGSP-----------PG----LDFLANA-------------GK-------------PGNWWGIAC--D-----------SK-G--KP---YIQTNEM-----EAPGFYVSTTALVD------SAF-K-ENH-------------PA------------KYVNAGKVPYIVLPSSP---------K--------------------FS-------
PKQ-----------KLGDLAMCFNTQ--N-GKKS------------WA-------------------------IYADV------------------------------------GP-------------KN------------------AIGEGSMHLS
>exp_virus_3300032783_____Ga0335079_10001352_24/27-166
GCAVDADG-S-------PR------AYHPP----TPEHP-----------SGRPPALDVIANA-------------GH-------------PGDWWGLAC--D-----------KN-G--LP---YKQGPGD-----PAPGFYVSTTALID------PTM-P-ESD-------------PR------------RYVDAETVPYASIPN---------LQP--------------------LL-------
ARV------------GPGDLGVALYKQ-----LTV------------PF-------------------------IIADV------------------------------------GP-------------RD------------------EIGEGSPALL
>exp_virus_3300009692_____Ga0116171_10006394_12/16-148
GMAIDADG-A-------PN------AYHA---------NN-----------IG----SDDLANA-------------GY-------------EGHWWGLAI--N-----------KAT-G--KP---YIQTGTD-----PCPGYYVSTTSLVN------PLR-D-DSE-------------PA------------RYVDSNTVPYIALGSNA---------C--------------------TA-------
YHM-----------APGAIARVEYRD-----LVV------------LA-------------------------VLADI------------------------------------GP-------------SG------------------KWGEGSIALA
>exp_virus_3300027741_____Ga0209085_1000149_41/28-169
GMQVDADG-S-------PK------AYH---------ATDP----------HGG-----LDDWSSA-------------GY-------------PNGHYQDILV-CD-----------EA-G--KP---IVQTGLS--AGDPAKGYFISATSLQR------PNY-S-KQD-------------VR------------RYVDAAVIPYAVINGALK--------K--------------------MV------
--EPK-----------FLGCRVEVENMR--L-GRTY------------NA-------------------------VCADV------------------------------------GP-------------AN------------------KLGEGSIALA
>exp_virus_2088090014_____GPIPI_17305223_22/34-170
DLDDDVDG-SE-------------RSYRL---------DND-----------PK-LALDDIHASA-------------GY-------------PHSSWWDVLV-------------RDPE-NRDKP-------------YVDKNGYCISMTSYQR-------------------------------------DGLSFYDRNRYVDAVNVPYSVAPGIIR------------------------------KL------
CKGV-----------LLGCASKITRIT--D-GAAI------------DC-------------------------VMADFS------------------------------------GY---S-IGEASDKAC
>exp_virus_3300028590_____Ga0247823_10000343_31/31-177
DADIDADG-A-------NGQHGKQPAYMV---------GN-----------RG----SEHLANGG--MITRG-----GKV----------VGNS-SWFRDIVICGA-----------D-G--QP---REFPGGVI----A-------SKTAYRF------PDM-A-VDD-------------PA------------AYVDSETVPYIVVPPIVR--------Q--------------------AT--
-----RGK-----------VMGCRARATNLT--N-GKQS------------WG-------------------------VVADI------------------------------------GP-------------RT------------------KTGEVSIEMA
>exp_virus_3300028592_____Ga0247822_10000132_31/31-177
DADIDADG-A-------NGQHGKQPAYMV---------GN-----------RG----SEHLANGG--MITRG-----GKV----------VGNS-SWFRDIVICGA-----------D-G--QP---REFPGGVI----A-------SKTAYRF------PDM-A-VDD-------------PA------------AYVDSETVPYIVVPPIVR--------Q--------------------AT--
-----RGK-----------VMGCRARATNLT--N-GKQS------------WG-------------------------VVADI------------------------------------GP-------------RT------------------KTGEVSIEMA
>exp_virus_3300032605_____Ga0316232_1002407_8/20-158
EMTIDADG-S-------PR------AY-------GP--------------KG-TSPLDDLDNA-------------GH-------------DGNWWGIAT--DN--------GASD-G--RP---LIQGQHD-----PFPGYYISTTAYQV------PGF-A-LTD-------------PH------------AYLDSEKVPFIVVPGRLV--------R--------------------AV-------
PGV------------VLGCKARITYPR--T-GKTV------------EA-------------------------VVGDR------------------------------------GP-------------ND------------------HIGEASMATA
>exp_virus_3300032668_____Ga0316230_1001206_42/20-158
EMTIDADG-S-------PR------AY-------GP--------------KG-TSPLDDLDNA-------------GH-------------DGNWWGIAT--DN--------GASD-G--RP---LIQGQHD-----PFPGYYISTTAYQV------PGF-A-LTD-------------PH------------AYLDSEKVPFIVVPGRLV--------R--------------------AV-------
PGV------------VLGCKARITYPR--T-GKTV------------EA-------------------------VVGDR------------------------------------GP-------------ND------------------HIGEASMATA
>exp_virus_3300032722_____Ga0316231_1001296_42/20-158
EMTIDADG-S-------PR------AY-------GP--------------KG-TSPLDDLDNA-------------GH-------------DGNWWGIAT--DN--------GASD-G--RP---LIQGQHD-----PFPGYYISTTAYQV------PGF-A-LTD-------------PH------------AYLDSEKVPFIVVPGRLV--------R--------------------AV-------
PGV------------VLGCKARITYPR--T-GKTV------------EA-------------------------VVGDR------------------------------------GP-------------ND------------------HIGEASMATA
>exp_virus_3300032677_____Ga0316227_1000899_11/26-160
ELCIDADG-S-------PR------AY-------GPN-G-----------QG-----LDHLENA-------------GY-------------PGNWWGIVT--D-----------KE-G--YP---VLQGAND-----PCPGFYISTTSLQH------KEF-E-TTN-------------PH------------RYIDSEQVPFIVVPGPVA---------R--------------------LA-------RGI-----
-------VLGCRAEILCQS--T-GARV------------SA-------------------------VVADI------------------------------------GP-------------AT------------------HLGEASIAAA
>exp_virus_3300025778_____Ga0208388_1000019_59/25-160
EACIDADG-S-------PR------AY-------GPN-D-----------TG-----LDYTANA-------------GH-------------LGNWWALVT--D-----------LS-G--NP---VIQGEGD-----PAPGFYVSTTSLVA------NPL-KPKTS-------------PL------------HWVNSEEIPYIVVPGPLA--------R--------------------AA-------QGV-
-----------VLGCHATITDSK--T-RNTI------------AA-------------------------VVADI------------------------------------GP-------------AT------------------HLGEMSIAAA
>exp_virus_3300003813_____Ga0007879_1000018_47/26-163
RADVDADG-S-------PR------AY-------APT-K-----------SG-LPTLDYLINA-------------ED-------------GDRWCGIVT--D-----------DD-G--YP---VIQGEND-----PAPGYYISTTTLQH------KQC-A-TSD-------------PA------------RYVDSENVSYVVINRAIA--------K--------------------AC-------
KGV------------VLGSRCTIKDTI--S-GILV------------EA-------------------------VVADT------------------------------------GP-------------AR------------------KLGEISIRAA
>exp_virus_3300003814_____Ga0007877_1000002_55/26-163
RADVDADG-S-------PR------AY-------APT-K-----------SG-LPTLDYLINA-------------ED-------------GDRWCGIVT--D-----------DD-G--YP---VIQGEND-----PAPGYYISTTTLQH------KQC-A-TSD-------------PA------------RYVDSENVSYVVINRAIA--------K--------------------AC-------
KGV------------VLGSRCTIKDTI--S-GILV------------EA-------------------------VVADT------------------------------------GP-------------AR------------------KLGEISIRAA
>exp_virus_3300006101_____Ga0007810_1000012_55/26-163
RADVDADG-S-------PR------AY-------APT-K-----------SG-LPTLDYLINA-------------ED-------------GDRWCGIVT--D-----------DD-G--YP---VIQGEND-----PAPGYYISTTTLQH------KQC-A-TSD-------------PA------------RYVDSENVSYVVINRAIA--------K--------------------AC-------
KGV------------VLGSRCTIKDTI--S-GILV------------EA-------------------------VVADT------------------------------------GP-------------AR------------------KLGEISIRAA
>exp_virus_3300006104_____Ga0007882_10000071_14/26-163
RADVDADG-S-------PR------AY-------APT-K-----------SG-LPTLDYLINA-------------ED-------------GDRWCGIVT--D-----------DD-G--YP---VIQGEND-----PAPGYYISTTTLQH------KQC-A-TSD-------------PA------------RYVDSENVSYVVINRAIA--------K--------------------AC-------
KGV------------VLGSRCTIKDTI--S-GILV------------EA-------------------------VVADT------------------------------------GP-------------AR------------------KLGEISIRAA
>exp_virus_3300006110_____Ga0007871_1000025_23/26-163
RADVDADG-S-------PR------AY-------APT-K-----------SG-LPTLDYLINA-------------ED-------------GDRWCGIVT--D-----------DD-G--YP---VIQGEND-----PAPGYYISTTTLQH------KQC-A-TSD-------------PA------------RYVDSENVSYVVINRAIA--------K--------------------AC-------
KGV------------VLGSRCTIKDTI--S-GILV------------EA-------------------------VVADT------------------------------------GP-------------AR------------------KLGEISIRAA
>exp_virus_3300006117_____Ga0007818_1000028_21/26-163
RADVDADG-S-------PR------AY-------APT-K-----------SG-LPTLDYLINA-------------ED-------------GDRWCGIVT--D-----------DD-G--YP---VIQGEND-----PAPGYYISTTTLQH------KQC-A-TSD-------------PA------------RYVDSENVSYVVINRAIA--------K--------------------AC-------
KGV------------VLGSRCTIKDTI--S-GILV------------EA-------------------------VVADT------------------------------------GP-------------AR------------------KLGEISIRAA
>exp_virus_3300025381_____Ga0208871_1000019_6/26-163
RADVDADG-S-------PR------AY-------APT-K-----------SG-LPTLDYLINA-------------ED-------------GDRWCGIVT--D-----------DD-G--YP---VIQGEND-----PAPGYYISTTTLQH------KQC-A-TSD-------------PA------------RYVDSENVSYVVINRAIA--------K--------------------AC-------
KGV------------VLGSRCTIKDTI--S-GILV------------EA-------------------------VVADT------------------------------------GP-------------AR------------------KLGEISIRAA
>exp_virus_3300025391_____Ga0208740_1000034_55/26-163
RADVDADG-S-------PR------AY-------APT-K-----------SG-LPTLDYLINA-------------ED-------------GDRWCGIVT--D-----------DD-G--YP---VIQGEND-----PAPGYYISTTTLQH------KQC-A-TSD-------------PA------------RYVDSENVSYVVINRAIA--------K--------------------AC-------
KGV------------VLGSRCTIKDTI--S-GILV------------EA-------------------------VVADT------------------------------------GP-------------AR------------------KLGEISIRAA
>exp_virus_3300025400_____Ga0208387_1000027_6/26-163
RADVDADG-S-------PR------AY-------APT-K-----------SG-LPTLDYLINA-------------ED-------------GDRWCGIVT--D-----------DD-G--YP---VIQGEND-----PAPGYYISTTTLQH------KQC-A-TSD-------------PA------------RYVDSENVSYVVINRAIA--------K--------------------AC-------
KGV------------VLGSRCTIKDTI--S-GILV------------EA-------------------------VVADT------------------------------------GP-------------AR------------------KLGEISIRAA

>exp_virus_3300025403_____Ga0208745_1000041_13/26-163
RADVDADG-S-------PR------AY-------APT-K-----------SG-LPTLDYLINA------------ED------------GDRWCGIVT--D----------DD-G--YP---VIQGEND-----PAPGYYISTTTLQH------KQC-A-TSD------------PA-----------RYVDSENVSYVVINRAIA-------K--------------------AC-------
KGV-----------VLGSRCTIKDTI--S-GILV-----------EA----------------------VVADT--------------------------------GP------------AR----------------KLGEISIRAA
>exp_virus_3300025416_____Ga0208877_1000289_14/26-163
RADVDADG-S-------PR------AY-------APT-K-----------SG-LPTLDYLINA------------ED------------GDRWCGIVT--D----------DD-G--YP---VIQGEND-----PAPGYYISTTTLQH------KQC-A-TSD------------PA-----------RYVDSENVSYVVINRAIA-------K--------------------AC-------
KGV-----------VLGSRCTIKDTI--S-GILV-----------EA----------------------VVADT--------------------------------GP------------AR----------------KLGEISIRAA
>exp_virus_3300025423_____Ga0208746_1000050_6/26-163
RADVDADG-S-------PR------AY-------APT-K-----------SG-LPTLDYLINA------------ED------------GDRWCGIVT--D----------DD-G--YP---VIQGEND-----PAPGYYISTTTLQH------KQC-A-TSD------------PA-----------RYVDSENVSYVVINRAIA-------K--------------------AC-------
KGV-----------VLGSRCTIKDTI--S-GILV-----------EA----------------------VVADT--------------------------------GP------------AR----------------KLGEISIRAA
>exp_virus_3300025430_____Ga0208622_1000032_55/26-163
RADVDADG-S-------PR------AY-------APT-K-----------SG-LPTLDYLINA------------ED------------GDRWCGIVT--D----------DD-G--YP---VIQGEND-----PAPGYYISTTTLQH------KQC-A-TSD------------PA-----------RYVDSENVSYVVINRAIA-------K--------------------AC-------
KGV-----------VLGSRCTIKDTI--S-GILV-----------EA----------------------VVADT--------------------------------GP------------AR----------------KLGEISIRAA
>exp_virus_3300025648_____Ga0208507_1000129_46/26-163
RADVDADG-S-------PR------AY-------APT-K-----------SG-LPTLDYLINA------------ED------------GDRWCGIVT--D----------DD-G--YP---VIQGEND-----PAPGYYISTTTLQH------KQC-A-TSD------------PA-----------RYVDSENVSYVVINRAIA-------K--------------------AC-------
KGV-----------VLGSRCTIKDTI--S-GILV-----------EA----------------------VVADT--------------------------------GP------------AR----------------KLGEISIRAA
>exp_virus_3300025783_____Ga0208258_1000037_53/26-163
RADVDADG-S-------PR------AY-------APT-K-----------SG-LPTLDYLINA------------ED------------GDRWCGIVT--D----------DD-G--YP---VIQGEND-----PAPGYYISTTTLQH------KQC-A-TSD------------PA-----------RYVDSENVSYVVINRAIA-------K--------------------AC-------
KGV-----------VLGSRCTIKDTI--S-GILV-----------EA----------------------VVADT--------------------------------GP------------AR----------------KLGEISIRAA
>exp_virus_3300003814_____Ga0007877_1000132_8/25-160
GLSVDADG-A-------PN------CY-------SPDPR-----------VG----LDDLGNA------------GH------------AGNWWGVVT--N----------DY-G--SP---ILQGPSD-----PFPNFYISTTSLQN------KNY-G-RTD------------PR-----------RYVNADVVPYIVVPVIIA-------R--------------------EA-------TGV-
----------VLGCVATVEDIR--T-NRKV-----------SG----------------------VVADL--------------------------------GP------------NN----------------HIGEASVAMA
>exp_virus_3300006104_____Ga0007882_10001047_17/25-160
GLSVDADG-A-------PN------CY-------SPDPR-----------VG----LDDLGNA------------GH------------AGNWWGVVT--N----------DY-G--SP---ILQGPSD-----PFPNFYISTTSLQN------KNY-G-RTD------------PR-----------RYVNADVVPYIVVPVIIA-------R--------------------EA-------TGV-
----------VLGCVATVEDIR--T-NRKV-----------SG----------------------VVADL--------------------------------GP------------NN----------------HIGEASVAMA
>exp_virus_3300006105_____Ga0007819_1000232_18/25-160
GLSVDADG-A-------PN------CY-------SPDPR-----------VG----LDDLGNA------------GH------------AGNWWGVVT--N----------DY-G--SP---ILQGPSD-----PFPNFYISTTSLQN------KNY-G-RTD------------PR-----------RYVNADVVPYIVVPVIIA-------R--------------------EA-------TGV-
----------VLGCVATVEDIR--T-NRKV-----------SG----------------------VVADL--------------------------------GP------------NN----------------HIGEASVAMA
>exp_virus_3300025430_____Ga0208622_1000101_25/25-160
GLSVDADG-A-------PN------CY-------SPDPR-----------VG----LDDLGNA------------GH------------AGNWWGVVT--N----------DY-G--SP---ILQGPSD-----PFPNFYISTTSLQN------KNY-G-RTD------------PR-----------RYVNADVVPYIVVPVIIA-------R--------------------EA-------TGV-
----------VLGCVATVEDIR--T-NRKV-----------SG----------------------VVADL--------------------------------GP------------NN----------------HIGEASVAMA
>exp_virus_3300025648_____Ga0208507_1000958_17/25-160
GLSVDADG-A-------PN------CY-------SPDPR-----------VG----LDDLGNA------------GH------------AGNWWGVVT--N----------DY-G--SP---ILQGPSD-----PFPNFYISTTSLQN------KNY-G-RTD------------PR-----------RYVNADVVPYIVVPVIIA-------R--------------------EA-------TGV-
----------VLGCVATVEDIR--T-NRKV-----------SG----------------------VVADL--------------------------------GP------------NN----------------HIGEASVAMA
>exp_virus_3300025789_____Ga0208499_1000259_22/25-160
GLSVDADG-A-------PN------CY-------SPDPR-----------VG----LDDLGNA------------GH------------AGNWWGVVT--N----------DY-G--SP---ILQGPSD-----PFPNFYISTTSLQN------KNY-G-RTD------------PR-----------RYVNADVVPYIVVPVIIA-------R--------------------EA-------TGV-
----------VLGCVATVEDIR--T-NRKV-----------SG----------------------VVADL--------------------------------GP------------NN----------------HIGEASVAMA
>exp_virus_3300022602_____Ga0248169_101248_4/25-160
GLTVDADG-A-------PN------CY-------SPDPR-----------VG----LDDLGNA------------GH------------TGNWWGIVT--N----------DY-G--SP---ILQGPSD-----PFPNFYISTTSLQN------KNY-A-RTD------------PR-----------RYVNADVVPFIVVPVIIA-------R--------------------EA-------TGV---
--------VLGCVATVEDIR--T-NRKV-----------QG----------------------VVADL--------------------------------GP------------NN----------------HIGEASVAMA
>exp_virus_3300004806_____Ga0007854_10000095_18/55-191
GLTIDADG-C-------PR------CY-------APE-S----------SG-LPTLDYISNA------------GN------------TNNWWGIVT-------------KN-G--VP---VIQGKND-----PYPGYYISTTSYIH------KEF-A-TDD------------PR-----------RYLDSEKVPFVVLPSPIF-------K--------------------MV-------KPI------
-----VLGSKVKVTNLK--N-GKIA-----------EG----------------------LVGDV--------------------------------GP------------AN----------------HLGEASMAMA
>exp_virus_3300006109_____Ga0007870_1000022_19/55-191
GLTIDADG-C-------PR------CY-------APE-S----------SG-LPTLDYISNA------------GN------------TNNWWGIVT-------------KN-G--VP---VIQGKND-----PYPGYYISTTSYIH------KEF-A-TDD------------PR-----------RYLDSEKVPFVVLPSPIF-------K--------------------MV-------KPI------
-----VLGSKVKVTNLK--N-GKIA-----------EG----------------------LVGDV--------------------------------GP------------AN----------------HLGEASMAMA
>exp_virus_3300006111_____Ga0007848_1000027_18/55-191
GLTIDADG-C-------PR------CY-------APE-S----------SG-LPTLDYISNA------------GN------------TNNWWGIVT-------------KN-G--VP---VIQGKND-----PYPGYYISTTSYIH------KEF-A-TDD------------PR-----------RYLDSEKVPFVVLPSPIF-------K--------------------MV-------KPI------
-----VLGSKVKVTNLK--N-GKIA-----------EG----------------------LVGDV--------------------------------GP------------AN----------------HLGEASMAMA
>exp_virus_3300006120_____Ga0007867_1000028_67/55-191
GLTIDADG-C-------PR------CY-------APE-S----------SG-LPTLDYISNA------------GN------------TNNWWGIVT-------------KN-G--VP---VIQGKND-----PYPGYYISTTSYIH------KEF-A-TDD------------PR-----------RYLDSEKVPFVVLPSPIF-------K--------------------MV-------KPI------
-----VLGSKVKVTNLK--N-GKIA-----------EG----------------------LVGDV--------------------------------GP------------AN----------------HLGEASMAMA
>exp_virus_3300025390_____Ga0208743_1000010_121/55-191
GLTIDADG-C-------PR------CY-------APE-S----------SG-LPTLDYISNA------------GN------------TNNWWGIVT-------------KN-G--VP---VIQGKND-----PYPGYYISTTSYIH------KEF-A-TDD------------PR-----------RYLDSEKVPFVVLPSPIF-------K--------------------MV-------KPI------
-----VLGSKVKVTNLK--N-GKIA-----------EG----------------------LVGDV--------------------------------GP------------AN----------------HLGEASMAMA
>exp_virus_3300025395_____Ga0208109_1000076_60/55-191
GLTIDADG-C-------PR------CY-------APE-S----------SG-LPTLDYISNA------------GN------------TNNWWGIVT-------------KN-G--VP---VIQGKND-----PYPGYYISTTSYIH------KEF-A-TDD------------PR-----------RYLDSEKVPFVVLPSPIF-------K--------------------MV-------KPI------
-----VLGSKVKVTNLK--N-GKIA-----------EG----------------------LVGDV--------------------------------GP------------AN----------------HLGEASMAMA
>exp_virus_3300025410_____Ga0208875_1000369_6/55-191
GLTIDADG-C-------PR------CY-------APE-S----------SG-LPTLDYISNA------------GN------------TNNWWGIVT-------------KN-G--VP---VIQGKND-----PYPGYYISTTSYIH------KEF-A-TDD------------PR-----------RYLDSEKVPFVVLPSPIF-------K--------------------MV-------KPI------
-----VLGSKVKVTNLK--N-GKIA-----------EG----------------------LVGDV--------------------------------GP------------AN----------------HLGEASMAMA
>exp_virus_3300025838_____Ga0208872_1000043_121/55-191
GLTIDADG-C-------PR------CY-------APE-S----------SG-LPTLDYISNA------------GN------------TNNWWGIVT-------------KN-G--VP---VIQGKND-----PYPGYYISTTSYIH------KEF-A-TDD------------PR-----------RYLDSEKVPFVVLPSPIF-------K--------------------MV-------KPI------
-----VLGSKVKVTNLK--N-GKIA-----------EG----------------------LVGDV--------------------------------GP------------AN----------------HLGEASMAMA
>exp_virus_3300028590_____Ga0247823_10000072_83/21-156
ELTVDGDG-S-------PR------CY-------GP------------PG-TKPLDFLANA------------GN------------PGNWWGIAT--D----------SK-G--IP---IIQGKDD-----PYPGYYVSTTALKN------KGF-K-HGD------------PR-----------REIDSEKVPFIVVPSPLI-------K--------------------KV-------RGV-----
------VLGSQAKIIDTR--T-GKFV-----------MA----------------------VVADI--------------------------------GP------------AK----------------HLGEASIAAA
>exp_virus_3300027573_____Ga0208454_1000129_10/32-170
KMSIDGDG-G-------YR------TYHE------NDD-----------VA----LDYLANA------------GY------------PGNWWGVAT--DT--------GEGD-G--DP---VYQKSTD-----PCPGYYVSPTAYEH------SDY-A-HTD------------PR-----------RYLDSESVIFSVTPPQLR-------Q--------------------LV-------
GPV-----------VLGCRVRMENLE--N-GLVA-----------WG----------------------LVGDV--------------------------------GP------------KD----------------KLGEGSIAAA
>exp_virus_3300028590_____Ga0247823_10000159_19/25-157
GMTIDADG-G-------RR------TYAP------PNSGL----------KA----LDYLANA------------GE------------PGNWYGLVT--DS----------K-G--KP---IVAKS----------GYYISPTTYER------KEF-S-RTN------------PE-----------RYLDPESEFFAVFPKQLR-------K--------------------MV-------KPV-----
------VLGCRVIVQDLR--N-GRTR-----------EG----------------------VAGDA--------------------------------GP------------ST----------------HLGEASIAYA
>exp_virus_3300005526_____Ga0073909_10000096_22/26-166
EMTIDADG-C-------SR------AY-------GP-------------EG-TKPLDYLANA------------GY------------PGNWWGVVT--D----------NS-G--EP---YVQSGSDGPHKAPWPGYYVSTTAYLV------SGY-D-KYD------------VR-----------RYVDSEKVPFAVVPGSVR-------T--------------------AI-----
--PPK-----------FMGCKVIVTDMK--T-GVEL-----------DC----------------------ACCDV--------------------------------GP------------SN----------------HLGEASMAAA
>exp_virus_3300005526_____Ga0073909_10000042_8/28-166
EFTVDGDG-S-------PR------CY---GPE---------GTDPLDWLANAGYPGNWWGICTHNQQSS-G--YP---I-LQGGTN----PWPGYHVSTTAYVN------SKY-Y-YAD------------PR-----------RYLDSENVLFAVIPGNVR-------K--------------------AV-------
AGI-----------CKGCRARVTDKR--T-GKAV-----------DC----------------------VIGDI--------------------------------GP------------SD----------------HMGEGSIRLA
>exp_virus_3300000553_____TBL_comb47_HYPODRAFT_10001047_26/37-126
DMDIDSDG-G--PNV---------DHDP-CWQPDTTLH-F----------N-G--HA---I--NSQIVPYVVAPIGLF-------E--------------------KV-------KGI----------GLGCKCIITHLK--N-KLSC--
----------DA-----------------------VCADL--------------------------------GP------------AT----------------KDGEGSAALA
>exp_virus_3300020699_____Ga0214221_1000072_41/37-126
DMDIDSDG-G--PNV---------DHDP-CWQPDTTLH-F----------N-G--HA---I--NSQIVPYVVAPIGLF-------E--------------------KV-------KGI----------GLGCKCIITHLK--N-KLSC--
----------DA-----------------------VCADL--------------------------------GP------------AT----------------KDGEGSAALA
>exp_virus_3300020700_____Ga0214225_1000035_38/37-126
DMDIDSDG-G--PNV---------DHDP-CWQPDTTLH-F----------N-G--HA---I--NSQIVPYVVAPIGLF-------E--------------------KV-------KGI----------GLGCKCIITHLK--N-KLSC--
----------DA-----------------------VCADL--------------------------------GP------------AT----------------KDGEGSAALA
>exp_virus_3300020703_____Ga0214227_1000027_17/37-126
DMDIDSDG-G--PNV---------DHDP-CWQPDTTLH-F----------N-G--HA---I--NSQIVPYVVAPIGLF-------E--------------------KV-------KGI----------GLGCKCIITHLK--N-KLSC--
----------DA-----------------------VCADL--------------------------------GP------------AT----------------KDGEGSAALA
>exp_virus_3300020706_____Ga0214217_1000118_28/37-126
DMDIDSDG-G--PNV---------DHDP-CWQPDTTLH-F----------N-G--HA---I--NSQIVPYVVAPIGLF-------E--------------------KV-------KGI----------GLGCKCIITHLK--N-KLSC--
----------DA-----------------------VCADL--------------------------------GP------------AT----------------KDGEGSAALA
>exp_virus_3300025316_____Ga0209697_10002550_20/42-131
DMDIDSDG-G--PNV---------DKDP-CWQPDTTLR-H----------A-G--GS---I--NAQTVPYVVCPIGVL-------D--------------------LV-------HEI----------GLGCRCVVTHTK--N-
RREA-----------VA-----------------------VLADL--------------------------------GP------------RA----------------KTGEGSPALA
>exp_virus_3300019888_____Ga0193751_1000386_16/31-118
DMDVDVDG-S--PNW---------RIDP-CGQPDTTLH-K----------D-G--EP---I--NSDAVPGIVLPPECI-------K--------------------FV-------SDI----------VLGCKALVTYR-----GRSS----
--------DA-----------------------VVFDV--------------------------------GP------------HN----------------KLGEGSAALA
>exp_virus_3300020062_____Ga0193724_1000025_14/37-124
DCDEDKDG-S--PDW---------HNDP-TGASGTSLH-Y----------N-G--KP---L--NGNIIPFIVIPPEIA-------K--------------------LT-------PDR----------FLGCYGTLEWH-----AKIA-----
-------PV-----------------------VVGDA--------------------------------GP------------HD----------------KVGEASSAAL
>exp_virus_3300022534_____Ga0224452_1000001_36/40-127
DADNDVDG-A--PYW---------HEDQ-YGQAGTRWT-Y----------N-G--KP---I--NGDVIPYIVVPPQVI-------K--------------------MT-------PEI----------VGGCIGSMYYK-----
DKEV------------AC-----------------------VVGDS--------------------------------GP------------TS----------------KIGEASPAAL
>exp_virus_3300022694_____Ga0222623_10000004_27/40-127
DADNDVDG-A--PYW---------HEDQ-YGQAGTRWT-Y----------N-G--KP---I--NGDVIPYIVVPPQVI-------K--------------------MT-------PEI----------VGGCIGSMYYK-----
DKEV------------AC-----------------------VVGDS--------------------------------GP------------TS----------------KIGEASPAAL
>exp_virus_3300009502_____Ga0114951_10000439_63/57-146
HAYIDDDG-S--DNR---------HHDP-CWQPQTSLH-P----------D-G--GA---L--DAESVPYIVVPPLIL-------L--------------------GV-------AGV----------VLGCQAHCRNTI--N-
GSEC------------DA-----------------------VVGDV--------------------------------GP------------HE----------------RIGEMSCECA
>exp_virus_3300029798_____Ga0239581_1000219_64/57-146
HAYIDDDG-S--DNR---------HHDP-CWQPQTSLH-P----------D-G--GA---L--DAESVPYIVVPPLIL-------L--------------------GV-------AGV----------VLGCQAHCRNTI--N-
GSEC------------DA-----------------------VVGDV--------------------------------GP------------HE----------------RIGEMSCECA
>exp_virus_3300022555_____Ga0212088_10005809_10/24-113
KAAIDCDG-S--DNR---------HNDP-CWQADTSLH-H----------D-G--KP---I--DAETVPGIVVPPLVI-------Q--------------------SV-------KGI----------VLGCQAWVRNLR--T-
GQET------------AA-----------------------VVFDI--------------------------------GP------------RR----------------KIGEMSCECA
>exp_virus_3300025162_____Ga0209083_1001017_31/24-113
KAAIDCDG-S--DNR---------HNDP-CWQADTSLH-H----------D-G--KP---I--DAETVPGIVVPPLVI-------Q--------------------SV-------KGI----------VLGCQAWVRNLR--T-
GQET------------AA-----------------------VVFDI--------------------------------GP------------RR----------------KIGEMSCECA
>exp_virus_3300009164_____Ga0114975_10000755_9/31-118
KAAIDCDG-G--SNP---------HHDP-CWQPDTTLR-F----------N-G--RS---I--DAEVVPFIVVPPLII-------Q--------------------RT-------RGI----------VMGCKA--RASY--R-GRSV----
--------DC-----------------------VVADI--------------------------------GP------------RR----------------KIGELSPAAA
>exp_virus_3300009184_____Ga0114976_10000585_26/31-118
KAAIDCDG-G--SNP---------HHDP-CWQPDTTLR-F----------N-G--RS---I--DAEVVPFIVVPPLII-------Q--------------------RT-------RGI----------VMGCKA--RASY--R-GRSV----
--------DC-----------------------VVADI--------------------------------GP------------RR----------------KIGELSPAAA
>exp_virus_3300027734_____Ga0209087_1000582_26/31-118
KAAIDCDG-G--SNP---------HHDP-CWQPDTTLR-F----------N-G--RS---I--DAEVVPFIVVPPLII-------Q--------------------RT-------RGI----------VMGCKA--RASY--R-GRSV----
--------DC-----------------------VVADI--------------------------------GP------------RR----------------KIGELSPAAA
>exp_virus_3300027969_____Ga0209191_1000371_28/31-118
KAAIDCDG-G--SNP---------HHDP-CWQPDTTLR-F----------N-G--RS---I--DAEVVPFIVVPPLII-------Q--------------------RT-------RGI----------VMGCKA--RASY--R-GRSV----
--------DC-----------------------VVADI--------------------------------GP------------RR----------------KIGELSPAAA
>exp_virus_3300009158_____Ga0114977_10000687_27/30-117
KAAIDCDG-G--SNP---------HHDP-CWQPDTTLR-F----------N-G--RS---I--DADVVPFIVVPPLII-------Q--------------------RT-------RGI----------VMGCKA--RASY--R-GRSV----
--------DC-----------------------VVADI--------------------------------GP------------RR----------------KIGELSPAAA
>exp_virus_3300009184_____Ga0114976_10000610_27/30-117
KAAIDCDG-G--SNP---------HHDP-CWQPDTTLR-F----------N-G--RS---I--DADVVPFIVVPPLII-------Q--------------------RT-------RGI----------VMGCKA--RASY--R-GRSV----
--------DC-----------------------VVADI--------------------------------GP------------RR----------------KIGELSPAAA
>exp_virus_3300027733_____Ga0209297_1000613_6/30-117

```
KAAIDCDG-G---------------------------------------------------------------------------SNP---------HHDP-CWQPDTTLR-F-----------N-G--RS---I----------------------------------------------------------------------------------------------------------DADVVPFIVVPPLII--------Q--------------------RT-------RGI-----------VMGCKA--RASY--R-GRSV----
--------DC--------------------------VVADI-----------------------------------GP-------------RR------------------KIGELSPAAA
>exp_virus_3300027734_____Ga0209087_1000604_27/30-117
KAAIDCDG-G---------------------------------------------------------------------------SNP---------HHDP-CWQPDTTLR-F-----------N-G--RS---I----------------------------------------------------------------------------------------------------------DADVVPFIVVPPLII--------Q--------------------RT-------RGI-----------VMGCKA--RASY--R-GRSV----
--------DC--------------------------VVADI-----------------------------------GP-------------RR------------------KIGELSPAAA
>exp_virus_3300027712_____Ga0209499_1000398_25/31-118
KAAIDCDG-G---------------------------------------------------------------------------SNP---------HHDP-CWQPDTTLR-F-----------N-G--RS---I----------------------------------------------------------------------------------------------------------DADVVPFIVVPPLII--------Q--------------------RT-------RGV-----------VMGCKA--RASY--R-GRSV---
---------DC--------------------------VVADI-----------------------------------GP-------------RR------------------KIGELSPAAA
>exp_virus_3300000439_____TBL_comb48_EPIDRAFT_1003500_8/26-115
WFRVDGDG-D-------P--------------------------------------------------------------TNH---------YHDP-CWQAETSLLGP-----------D-G--KP---I----------------------------------------------------------------------------------------------------------DAMQVPYIVANPIIA--------Q--------------------MV-------PGV-----------VLGCIGQATYR-----
GRTV-------------QA-------------------------VLGDI-----------------------------------GP-------------RR------------------KIGEGSIALA
>exp_virus_3300021142_____Ga0214192_1000169_29/26-115
WFRVDGDG-D-------P--------------------------------------------------------------TNH---------YHDP-CWQAETSLLGP-----------D-G--KP---I----------------------------------------------------------------------------------------------------------DAMQVPYIVANPIIA--------Q--------------------MV-------PGV-----------VLGCIGQATYR-----
GRTV-------------QA-------------------------VLGDI-----------------------------------GP-------------RR------------------KIGEGSIALA
>exp_virus_3300021124_____Ga0214199_1000026_7/27-116
WYRVDGDG-D-------P--------------------------------------------------------------TNH---------FHDR-CYQKDTSLHTP-----------D-G--KP---I----------------------------------------------------------------------------------------------------------DAYTLPYVVLNPYIA--------A--------------------SL-------PAV-----------FLGALALVSYK-----
GKTV-------------NA-------------------------VIGDV-----------------------------------GP-------------PH------------------KVGEGSEALA
>exp_virus_3300021139_____Ga0214166_1000301_5/27-116
WYRVDGDG-D-------P--------------------------------------------------------------TNH---------FHDR-CYQKDTSLHTP-----------D-G--KP---I----------------------------------------------------------------------------------------------------------DAYTLPYVVLNPYIA--------A--------------------SL-------PAV-----------FLGALALVSYK-----
GKTV-------------NA-------------------------VIGDV-----------------------------------GP-------------PH------------------KVGEGSEALA
>exp_virus_3300031524_____Ga0302320_10005537_8/30-119
KAAIDSDG-S---------------------------------------------------------------------------GSS---------HGDP-DQEQDTSLH-T-----------N-G--KP---L----------------------------------------------------------------------------------------------------------NADVDRYIVVPPSVI--------Q--------------------GT-------KGI-----------VLGCRAVVTNQL--T-GIQV-
-----------DC--------------------------VVGDI-----------------------------------GP-------------HH------------------KIGEISIATA
>exp_virus_3300031788_____Ga0302319_10002010_6/30-119
KAAIDSDG-S---------------------------------------------------------------------------GSS---------HGDP-DQEQDTSLH-T-----------N-G--KP---L----------------------------------------------------------------------------------------------------------NADVDRYIVVPPSVI--------Q--------------------GT-------KGI-----------VLGCRAVVTNQL--T-GIQV-
-----------DC--------------------------VVGDI-----------------------------------GP-------------HH------------------KIGEISIATA
>exp_virus_3300029922_____Ga0311363_10000523_93/27-113
EAMIDSDG-T---------------------------------------------------------------------------GSS---------HGDP-DQQAQTSYE-P-----------------Y---L--------------------------------------------------------------------------------------------------------NADLDKYIVLPPQLF--------A--------------------GV-------KPM-----------VLGCKVEVLNLT--N-GLYT-
-----------DA--------------------------VVGDQ-----------------------------------GP-------------SL------------------KIGEMSIATA
>exp_virus_3300010362_____Ga0126377_10000413_12/29-115
NLDVCTDG-T---------------------------------------------------------------------------GPA---------HGDT-SHLNKTAY-----------------T--PS---L--------------------------------------------------------------------------------------------------------NADVDFYIVLHPKMR--------T--------------------GV-------RPV-----------VLGCMGRVTNLR--T-
FKSH------------WG-------------------------VWGEV-----------------------------------GP-------------SN------------------KAGETAIVMA
>exp_virus_3300010362_____Ga0126377_10000082_45/30-120
DLDICNDG-S---------------------------------------------------------------------------GPP---------HGDP-SYQSETAYY-N-----------G-G--KY---L----------------------------------------------------------------------------------------------------------NADKDKYIVTPPQVR--------K--------------------MV-------PPV-----------VMGCQGRVTNLR--
KPAQSS------------WG-------------------------VVGDI-----------------------------------GP-------------DD------------------ITGEAAYCLA
>exp_virus_3300003911_____JGI25405J52794_10000041_18/28-117
NLDICNDG-S---------------------------------------------------------------------------GPS---------HGDP-YYQSQTAYY-N-----------G-G--KY---L----------------------------------------------------------------------------------------------------------NADVDKYIVIPPQCR--------T--------------------MV-------PPV-----------VMGCQARMTNML--T-
AKWH------------AA-------------------------VTGEI-----------------------------------GP-------------SN------------------KTGEAAYCLA
>exp_virus_3300005937_____Ga0081455_10000183_26/28-117
NLDICNDG-S---------------------------------------------------------------------------GPS---------HGDP-YYQSQTAYY-N-----------G-G--KY---L----------------------------------------------------------------------------------------------------------NADVDKYIVIPPQCR--------T--------------------MV-------PPV-----------VMGCQARMTNML--T-
AKWH------------AA-------------------------VTGEI-----------------------------------GP-------------SN------------------KTGEAAYCLA
>exp_virus_3300010376_____Ga0126381_100000306_58/39-128
DLDVCNDG-S---------------------------------------------------------------------------GPS---------HGDP-SYQSQTAYY-N-----------N-G--KF---L----------------------------------------------------------------------------------------------------------NADKDFYIVIPPQVR--------S--------------------GV-------DPV-----------VMGCQAKLTRLD--T-
MESS------------PA-------------------------VTGEI-----------------------------------GP-------------DD------------------KTGEAAYCLA
>exp_virus_3300012971_____Ga0126369_10004632_5/39-128
NLDVCTDG-T---------------------------------------------------------------------------GPH---------HGDT-SPQDETAYY-N-----------G-G--KF---L----------------------------------------------------------------------------------------------------------NADVDKYIVIPPQVR--------S--------------------MV-------APV-----------VMGCQAKMTRLD--S-
MATS------------PA-------------------------VTGEI-----------------------------------GP-------------DD------------------KTGEAAYCLA
>exp_virus_3300012971_____Ga0126369_10000399_5/44-130
NLDVCTDG-T---------------------------------------------------------------------------GDH---------HGDN-SPLDQTAY-----------------N--PY---L--------------------------------------------------------------------------------------------------------NADKDRYIVIPPQVR--------M--------------------MP-------EPV-----------VLGCQGKLTRLD--S-
MKSS------------PG-------------------------VIGEI-----------------------------------GP-------------EN------------------KTGEAAICLA
>exp_virus_3300004633_____Ga0066395_10000709_14/42-131
DLDVCTDG-T---------------------------------------------------------------------------GNH---------HGDN-TPQDETAYY-N-----------G-G--KF---L----------------------------------------------------------------------------------------------------------NADVDKYIVIPPQIR--------S--------------------MV-------PPV-----------VMGCQARLTQLD--T-
QQAS------------PA-------------------------VTGEI-----------------------------------GP-------------DD------------------KTGEAAYCLC
>exp_virus_3300005764_____Ga0066903_100000166_28/44-133
DLDVCTDG-S---------------------------------------------------------------------------GPS---------HGDP-SYQPQTAYY-N-----------G-G--KF---L----------------------------------------------------------------------------------------------------------NADVDKYIVIPPQVR--------S--------------------MV-------APV-----------VMGCQGKLTRLD--S-
ALQH------------AA-------------------------VVGEI-----------------------------------GP-------------KD------------------KTGEAAICLA
>exp_virus_3300010361_____Ga0126378_10000419_32/39-128
DLDVCTDG-T---------------------------------------------------------------------------GDD---------HGDP-YYQSQTAYY-N-----------N-G--KF---L----------------------------------------------------------------------------------------------------------NADKDKYIVVPPQIR--------S--------------------MV-------PPV-----------VMGCQGKLTRLD--S-
MAHS------------AA-------------------------VVGEI-----------------------------------GP-------------SD------------------KTGEAAICLA
>exp_virus_3300010366_____Ga0126379_10000390_18/26-115
DLDICNDG-S---------------------------------------------------------------------------GPS---------HGDD-DYQSQTAYY-N-----------G-G--KY---L----------------------------------------------------------------------------------------------------------NADVDRYIVIPPQVR--------S--------------------MV-------PPV-----------VMGSRARLTNLL--T-
METS------------AA-------------------------VTGDI-----------------------------------GP-------------DD------------------KTGEAAYCLA
>exp_virus_3300005526_____Ga0073909_10000172_25/59-148
DLDICNDG-S---------------------------------------------------------------------------GPS---------HDDS-SYQSQTAYW-N-----------G-G--KF---L----------------------------------------------------------------------------------------------------------NADVDKYIVVPPQIR--------S--------------------MT-------AGV-----------VMGCQAKLTNLD--T-
GVET------------PA-------------------------VVGDI-----------------------------------GP-------------SS------------------KTGEAAYCAA
>exp_virus_3300027821_____Ga0209811_10000270_8/59-148
DLDICNDG-S---------------------------------------------------------------------------GPS---------HDDS-SYQSQTAYW-N-----------G-G--KF---L----------------------------------------------------------------------------------------------------------NADVDKYIVVPPQIR--------S--------------------MT-------AGV-----------VMGCQAKLTNLD--T-
GVET------------PA-------------------------VVGDI-----------------------------------GP-------------SS------------------KTGEAAYCAA
>exp_virus_3300010366_____Ga0126379_10000347_19/40-129
DLDVCNDG-T---------------------------------------------------------------------------GPR---------HGDK-HYQPQTAYY-N-----------K-G--KF---L----------------------------------------------------------------------------------------------------------NADVDPYIVIPMCLR--------K--------------------LL-------KKK-----------SMGCKAQLSQVE--S-
GAIF------------PA-------------------------VCGEL-----------------------------------GP-------------DN------------------KTGEASYCLA
>exp_virus_3300010376_____Ga0126381_100000274_36/29-118
DLDVCNDG-T---------------------------------------------------------------------------GPR---------HGDK-HYQPQTAYY-N-----------K-G--KF---L----------------------------------------------------------------------------------------------------------NADEECYIVIPMCVR--------K--------------------LL-------KKK-----------SMGCKAQASNIE--T-
GAIY------------PA-------------------------VCGEL-----------------------------------GP-------------ND------------------KTGEASYCLA
>exp_virus_3300010376_____Ga0126381_100000470_39/28-117
DLDICNDG-S---------------------------------------------------------------------------GPS---------HGDP-SYQTQTAYY-N-----------G-G--KY---L----------------------------------------------------------------------------------------------------------NADEDQYIVVPPQVR--------R--------------------MI-------VPV-----------VMGCQARLTNMK--T-
ALWY------------PA-------------------------MTGDI-----------------------------------GP-------------PD------------------KTGEAAYCLA
>exp_virus_3300010048_____Ga0126373_10000281_42/30-117
NLDIDNDG-T---------------------------------------------------------------------------GPS---------HGDP-SYQSQTTY-----------------S--PY---L--------------------------------------------------------------------------------------------------------NADVDQYVVTPPQVR--------K--------------------GV-------APV-----------VIGCQARVTNLH--
KPDQWG------------WG-------------------------VTGDV-----------------------------------GP-------------SN------------------KCGEAAYCLA
>exp_virus_3300006854_____Ga0075425_100000201_58/34-121
DLDVDVDG-I---------------------------------------------------------------------------GNS---------YGDP-YYQPDTTLH-Y-----------N-G--RA---L----------------------------------------------------------------------------------------------------------NSDVDYFIVLPPPLI--------K--------------------AV-------AGK-----------VLGCKAKVF--Y--R-
GKSC------------DA-------------------------VVGDV-----------------------------------GP-------------AR------------------KVGEASRAVA
>exp_virus_3300007076_____Ga0075435_100000075_12/34-121
DLDVDVDG-I---------------------------------------------------------------------------GNS---------YGDP-YYQPDTTLH-Y-----------N-G--RA---L----------------------------------------------------------------------------------------------------------NSDVDYFIVLPPPLI--------K--------------------AV-------AGK-----------VLGCKAKVF--Y--R-
GKSC------------DA-------------------------VVGDV-----------------------------------GP-------------AR------------------KVGEASRAVA
>exp_virus_3300010233_____Ga0136235_1000189_28/33-122
DMDIDVDG-S---------------------------------------------------------------------------GNP---------HGDP-YFQPDTTLH-Y-----------Q-G--KA---L----------------------------------------------------------------------------------------------------------DSDVDKFIVLPLPCL--------Q--------------------RV-------KGI-----------VLGCQARVVNTR--N-
GKFC------------DA-------------------------VVGDV-----------------------------------GP-------------SR------------------KIGEASRAVA
>exp_virus_3300010233_____Ga0136235_1000158_39/34-124
DADICCDG-S---------------------------------------------GG---------NP--------------------------------------------------------D-N--DP------------------YFQPDTAYHY-----------------------------------QG-------QALNAYKVPFIVVPKSII-------------------------------NA------VKGI-----------VLGCKARMTYIR----
TKMT-----------VEC-------------------------IVGDI-----------------------------------GP-------------SA------------------KIGECSPRAA
>exp_virus_3300010233_____Ga0136235_1000369_4/26-125
ALQIDGDG-------------------------------------------SG-------------------------LNL------------------------------------------E-G--DPD------------------FQDDTSLHYG---------DTTPD------------------YSDKT-----KALNSRKVPFFVFPPQLI-------------------------------RA------VPQV-----------VLG------S----G-
GTATNI--------NTNAS---------------------SPLVVGDV-----------------------------------GP-------------DD------------------KLGEGSICLA
>exp_virus_3300029953_____Ga0311343_10000842_75/28-119
GMDIDGDG-------------------------------------------SG-------------------------GNL------------------------------------------E-N--DPD------------------FQPTTSLR-------------NPD------------------GS---------SLNSRAECGIVLPGKLM-------------------------------RA------FQEI-----------ALGCRAYVRS----G---
LTGL--------FTDA-------------------------VVADG-----------------------------------GP-------------DD------------------KIGEAFIATA
>exp_virus_3300009154_____Ga0114963_10000394_11/43-133
DLDLDTDG-ADDVTV----------------------------------------------------------------------------------------------------------------VH-W--EK------------------YHQSQTSLD-----------------------------------PSGKI-------ISSVGVPYVVFPGWFA-------------------------------QA-------VGI-----------ALGSVCVVLD----G-
DREP-----------VYA-------------------------VFADY-----------------------------------GP-------------RK------------------KIGEGSIALH
>exp_virus_3300022745_____Ga0228698_1000621_24/49-136
DLDLDTDG-G-------HA-----------------------------------------------------------------PGI---------IWDA-THQDDTSLRWP-----------D-G--KP---V----------------------------------------------------------------------------------------------------------DSNSIPFVVIPGKWN------------------------------------------RGI-----------KLGDMCHVQYA-----
NKVV------------AA-------------------------IVADI-----------------------------------GP-------------QR------------------KVGEGSIALH
>exp_virus_3300021478_____Ga0210402_10000026_183/40-186
EMTIDGDG-G-------PH------TYNP--------ED-----------TG----LDFLANG-------------G------------YPDDGCYGMVTDPE-----------DS-E--DPADCILQEMYH-----PAPGYFVSATSLTN------EIF-P-PSS-------------PD------------HYVNSEEIPFIAVPGGHDDPD--VPGW---------------------DA------
-FGV------------SLGDVAFVWNTA--T-DDNC------------FA-------------------------LVADV-----------------------------------GP-------------SS------------------HAGEGSIKLA
>exp_virus_3300002408_____B570J29032_109959582_20/408-498
KLRVDTDG--------AP-------------------------------G-----NP--------------------------------------------------------Y-N--DP------------------DWQPETSL-------------RLN-------------------GT------P-VDASKVPYVVV-------------------------SPDA---Q-KA-------HGI-----------KTGDWALVTN----Q-
SNKQQ----------IWA-------------------------RVADV-----------------------------------GP-------------RGA-----------------SAGEMSAAAA
>exp_virus_3300002835_____B570J40625_100000081_20/408-498
KLRVDTDG--------AP-------------------------------G-----NP--------------------------------------------------------Y-N--DP------------------DWQPETSL-------------RLN-------------------GT------P-VDASKVPYVVV-------------------------SPDA---Q-KA-------HGI-----------KTGDWALVTN----Q-
SNKQQ----------IWA-------------------------RVADV-----------------------------------GP-------------RGA-----------------SAGEMSAAAA
>exp_virus_3300020550_____Ga0208600_1000021_2/408-498
KLRVDTDG--------AP-------------------------------G-----NP--------------------------------------------------------Y-N--DP------------------DWQPETSL-------------RLN-------------------GT------P-VDASKVPYVVV-------------------------SPDA---Q-KA-------HGI-----------KTGDWALVTN----Q-
SNKQQ----------IWA-------------------------RVADV-----------------------------------GP-------------RGA-----------------SAGEMSAAAA
>exp_virus_3300009185_____Ga0114971_10000002_127/162-246
TLNIDTDG-------------------------------------------IG---------SP------------------------------------------------------TFN--DS------------------TRQNQTSVQG-------------------------------------------LNANTQSFFVLPNKPE------YYK-Y-------------------KG-------KAIYAKN-----N--------------TTGKSI--------
----MG-------------------------IVGDF-----------------------------------GP-------------TTK-----------------GFGEMSLYAA
>exp_virus_3300027746_____Ga0209597_1000003_90/162-246
TLNIDTDG-------------------------------------------IG---------SP------------------------------------------------------TFN--DS------------------TRQNQTSVQG-------------------------------------------LNANTQSFFVLPNKPE------YYK-Y-------------------KG-------KAIYAKN-----N--------------TTGKSI--------
----MG-------------------------IVGDF-----------------------------------GP-------------TTK-----------------GFGEMSLYAA
>exp_virus_3300027911_____Ga0209698_10001340_22/62-158
KMAIDADGVAAG---------------------------------------PG-RRC--------------------GTQ--------------------------------------L--DPS------------------DGQNDTSFH-------------FPD--------------------GSP-------LSSERHPFIVLP---------------------------LGVFRA---------ATGL-----------SVGDLAVVIY----R-
DLLT------------AA-------------------------ICGDL-----------------------------------GP-------------SH------------------KIGEGSIRLH
```

Supplementary dataset 1. Multiple alignment of bacterial, fungal and viral chitosanase protein sequences. Sequences with headers containing ‘ref_bact’, ‘ref_fungi’ and ‘exp_virus’ are bacterial, fungal and viral chitosanase sequences, respectively. The chitosanase sequences were aligned with a phage lysozyme (YP_006987285, ‘OutGroup’) as an outgroup sequence for the phylogenetic tree construction. ‘-’ represents the gap within the alignments.
