# Supplementary material for: Structural characterization of a soil viral auxiliary metabolic gene product – a functional chitosanase
Source: Nat Commun. 2022 Sep 19;13:5485. doi: 10.1038/s41467-022-32993-8 (PMC9485262; doi:10.1038/s41467-022-32993-8)
Supplement: Supplementary file 5 — Reporting Summary [file 41467_2022_32993_MOESM5_ESM.pdf]

nature portfolio

Corresponding author(s): Janet K. Jansson

Last updated by author(s): 08/05/2022

Reporting Summary

Nature Portfolio wishes to improve the reproducibility of the work that we publish. This form provides structure for consistency and transparency in reporting. For further information on Nature Portfolio policies, see our Editorial Policies and the Editorial Policy Checklist.

Statistics

For all statistical analyses, confirm that the following items are present in the figure legend, table legend, main text, or Methods section.

n/a	Confirmed	
☐	☒	The exact sample size (*n*) for each experimental group/condition, given as a discrete number and unit of measurement
☐	☒	A statement on whether measurements were taken from distinct samples or whether the same sample was measured repeatedly
☒	☐	The statistical test(s) used AND whether they are one- or two-sided<br>*Only common tests should be described solely by name; describe more complex techniques in the Methods section.*
☒	☐	A description of all covariates tested
☒	☐	A description of any assumptions or corrections, such as tests of normality and adjustment for multiple comparisons
☐	☒	A full description of the statistical parameters including central tendency (e.g. means) or other basic estimates (e.g. regression coefficient) AND variation (e.g. standard deviation) or associated estimates of uncertainty (e.g. confidence intervals)
☒	☐	For null hypothesis testing, the test statistic (e.g. *F*, *t*, *r*) with confidence intervals, effect sizes, degrees of freedom and *P* value noted<br>*Give P values as exact values whenever suitable.*
☒	☐	For Bayesian analysis, information on the choice of priors and Markov chain Monte Carlo settings
☒	☐	For hierarchical and complex designs, identification of the appropriate level for tests and full reporting of outcomes
☒	☐	Estimates of effect sizes (e.g. Cohen's *d*, Pearson's *r*), indicating how they were calculated

Our web collection on statistics for biologists contains articles on many of the points above.

Software and code

Policy information about availability of computer code

Data collection	No software was used for data collection.
Data analysis	No custom code or custom mathematical algorithms were applied to this study.<br>The open-source software were used including EggNOG (v4.5), CAZY (dbCAN-HMMdb-V7), FOAM (rel1), Hmmer (v3.1b2), Prodigal (v2.6.3), blastp (v2.9.0+), checkV (v0.7.0), vConTACT (v2.0.9.10),WIsH (v1.0), VirHostMatcher (v1.0.0), Prokaryotic virus Host Predictor (PHP, no version infomation available), CD-HIT (v4.8.1), MAFFT (v7), RAxML (v1.0.1), XDS (v. Feb 5 2021), AIMLESS (v0.7.7), PHENIX (v1.20.1-4487), COOT (v0.9.8.2), MOLREP (v11.9.02), AlphaFold (v2.1.0), PyMOL (v2.5.2), ICM-Pro (v3.8-6a).

For manuscripts utilizing custom algorithms or software that are central to the research but not yet described in published literature, software must be made available to editors and reviewers. We strongly encourage code deposition in a community repository (e.g. GitHub). See the Nature Portfolio guidelines for submitting code & software for further information.

Data

Policy information about availability of data

All manuscripts must include a data availability statement. This statement should provide the following information, where applicable:

- Accession codes, unique identifiers, or web links for publicly available datasets
- A description of any restrictions on data availability
- For clinical datasets or third party data, please ensure that the statement adheres to our policy

The viral sequence data used for Figure 1 are publicly available on the JGI website [https://img.jgi.doe.gov/cgi-bin/vr/main.cgi] with no use restriction according to the JGI data policy. The atomic coordinates and structure factors for the protein structures have been submitted to the Protein Data Bank as follows: V-Csn apo1, PDB code 7TVL [http://doi.org/10.2210/pdb7TVL/pdb]; V-Csn apo2, 7TVM [http://doi.org/10.2210/pdb7TVM/pdb]; V-Csn-D148N, 7TVN [http://doi.org/10.2210/pdb7TVM/pdb]; V-Csn-E157Q, 7TVO [http://doi.org/10.2210/pdb7TVO/pdb]; V-Csn-E157Q chitohexaose complex, 7TVP [http://doi.org/10.2210/pdb7TVP/pdb]. The wwPDB X-ray structure validation reports are included as Supplementary Fig. 3. The supplementary information file contains three supplementary figures (Supplementary Fig. 1, 2 and 3) and one supplementary table (Supplementary Table 1). The source data underlying Figures 2a and 2c are provided as Source Data files.

Human research participants

Policy information about studies involving human research participants and Sex and Gender in Research.

Reporting on sex and gender	NA
Population characteristics	NA
Recruitment	NA
Ethics oversight	NA

Note that full information on the approval of the study protocol must also be provided in the manuscript.

Field-specific reporting

Please select the one below that is the best fit for your research. If you are not sure, read the appropriate sections before making your selection.

☐ Life sciences ☐ Behavioural & social sciences ☒ Ecological, evolutionary & environmental sciences

For a reference copy of the document with all sections, see nature.com/documents/nr-reporting-summary-flat.pdf

Ecological, evolutionary & environmental sciences study design

All studies must disclose on these points even when the disclosure is negative.

Study description	We screened AMGs that potentially encode chitosanase enzymes from a public database and expressed one showing endo-chitosanase activity (V-Csn). V-Csn was crystalized and structurally characterized at ultra-high resolution, thus representing the first structure of a soil viral AMG product. These findings support the hypothesis that soil viruses contribute auxiliary functions to their hosts. Soil viral chitosanase AMG products that assist chitin decomposition may therefore play a previously unrecognized role in soil carbon cycling.
Research sample	Viral contigs that carried GH75 chitosanase AMGs were retrieved from the Integrated Microbial Genomes and Virome (IMG/VR) database (v3.0). A total of 142 qualified GH75 chitosanase-like AMGs were identified from viral contigs with lengths ranging from 8 to 202 kb.
Sampling strategy	We screened AMGs that potentially encode chitosanase enzymes from the Integrated Microbial Genomes and Virome (IMG/VR) database (v3.0, https://img.jgi.doe.gov/cgi-bin/vr/main.cgi). IMG/VR is the latest and largest public viral database including more than two million well-curated viral sequences identified from global metagenomes and sequenced from viral isolates. The database is sufficient for us to detect soil viruses that carry chitosanase AMGs.
Data collection	The viral sequence data were extracted from the Integrated Microbial Genomes and Virome (IMG/VR) database (v3.0, https://img.jgi.doe.gov/cgi-bin/vr/main.cgi) by Ruonan Wu and David Paez-Espino and downloaded onto the High-Performance Computing Cluster at PNNL.
Timing and spatial scale	We accessed the Integrated Microbial Genomes and Virome (IMG/VR) database (v3.0) in Feb, 2021 to collect the viral sequence data. IMG/VR (v3) is currently the latest version. All the viral sequences with chitosanase AMGs, regardless of the habitats, were included and thus selection on spatial scale was not applied in this study.
Data exclusions	We excluded the data that had use-restriction according to the JGI data policy.

Reproducibility	The experiment shown in Figure 2 was replicated three times. The structural characterization was performed on individual samples.
Randomization	Randomization is not applicable to this study because we are dealing with a small number of samples.
Blinding	Blinding is not applicable to this study because it was necessary to track the individual sample ids during the experiments.

Did the study involve field work? ☐ Yes ☒ No

Reporting for specific materials, systems and methods

We require information from authors about some types of materials, experimental systems and methods used in many studies. Here, indicate whether each material, system or method listed is relevant to your study. If you are not sure if a list item applies to your research, read the appropriate section before selecting a response.

Materials & experimental systems

n/a	Involved in the study	
☒	☐	Antibodies
☒	☐	Eukaryotic cell lines
☒	☐	Palaeontology and archaeology
☒	☐	Animals and other organisms
☒	☐	Clinical data
☒	☐	Dual use research of concern

Methods

n/a	Involved in the study	
☒	☐	ChIP-seq
☒	☐	Flow cytometry
☒	☐	MRI-based neuroimaging
